# Microwave-irradiated ultrafast synthesis of NiSnPdPtRh high-entropy alloy nanorods for high-efficiency electrocatalytic hydrogen evolution reaction

**DOI:** 10.1039/d6sc06688g

**Published:** 2026-10-22

**Authors:** Yuan Fang, Siyuan Lai, Mairui Long, Keying Xing, Yanfang Wu, Junli Ren, Zhan Zhang, Wei Keat Ng, Tao Huang, Xiongwu Kang

**Affiliations:** a Key Laboratory of Catalysis and Energy Materials Chemistry of Ministry of Education, College of Chemistry and Materials Science, South-Central Minzu University Wuhan 430074 China huangt208@mail.scuec.edu.cn; b School of Environment and Energy, South China University of Technology Guangzhou 510006 China esxkang@scut.edu.cn; c State Key Laboratory of Advanced Papermaking and Paper-based Materials, School of Light Industry and Engineering, South China University of Technology Guangzhou 510640 China

## Abstract

Uniform and well-defined NiSnPdPtRh high-entropy alloy nanorods (HEA NRs) are successfully prepared by a one-pot microwave-irradiated strategy within 180 s. The growth mechanism involves simultaneous reduction of metal precursors, linear assembly of nanoclusters, and Ostwald ripening, with Sn and Pd playing critical roles in directing the anisotropic growth. After carbon-supported thermal treatment at 200 °C, the as-prepared NiSnPdPtRh@C-200 NRs exhibit exceptional electrocatalytic performance for acidic HER, achieving an ultra-low overpotential of 8.39 mV at 10 mA cm^−2^, a very low Tafel slope of 7.92 mV dec^−1^, and a high noble metal mass activity of 2564 A g^−1^. Even at an industrial-level current density of 2000 mA cm^−2^, an overpotential of only 196.5 mV is required. When deployed in a proton exchange membrane water electrolyzer (PEMWE), NiSnPdPtRh@C-200 NRs exhibit a low cell voltage of merely 1.85 and 1.98 V at 1.0 and 1.5 A cm^−2^, respectively, and display an extremely low voltage decay of just 12.19 µV h^−1^ after operating for 2000 h at 500 mA cm^−2^. DFT calculations reveal that Pt–Pt–Rh and Ni–Pt–Pd hollow sites serve as synergistic active centres, facilitating efficient HER kinetics *via* a Tafel mechanism. This work provides a simple, rapid synthesis strategy for HEA nanocrystals as high-efficiency HER electrocatalysts.

## Abstract 2

NiSnPdPtRh high-entropy alloy nanorods (HEA NRs) are rapidly synthesized in 180 s *via* microwave irradiation. After carbon-supported heat treatment, the as-prepared NiSnPdPtRh@C NRs display exceptional HER performance and electrochemical durability.

## Graphical abstract

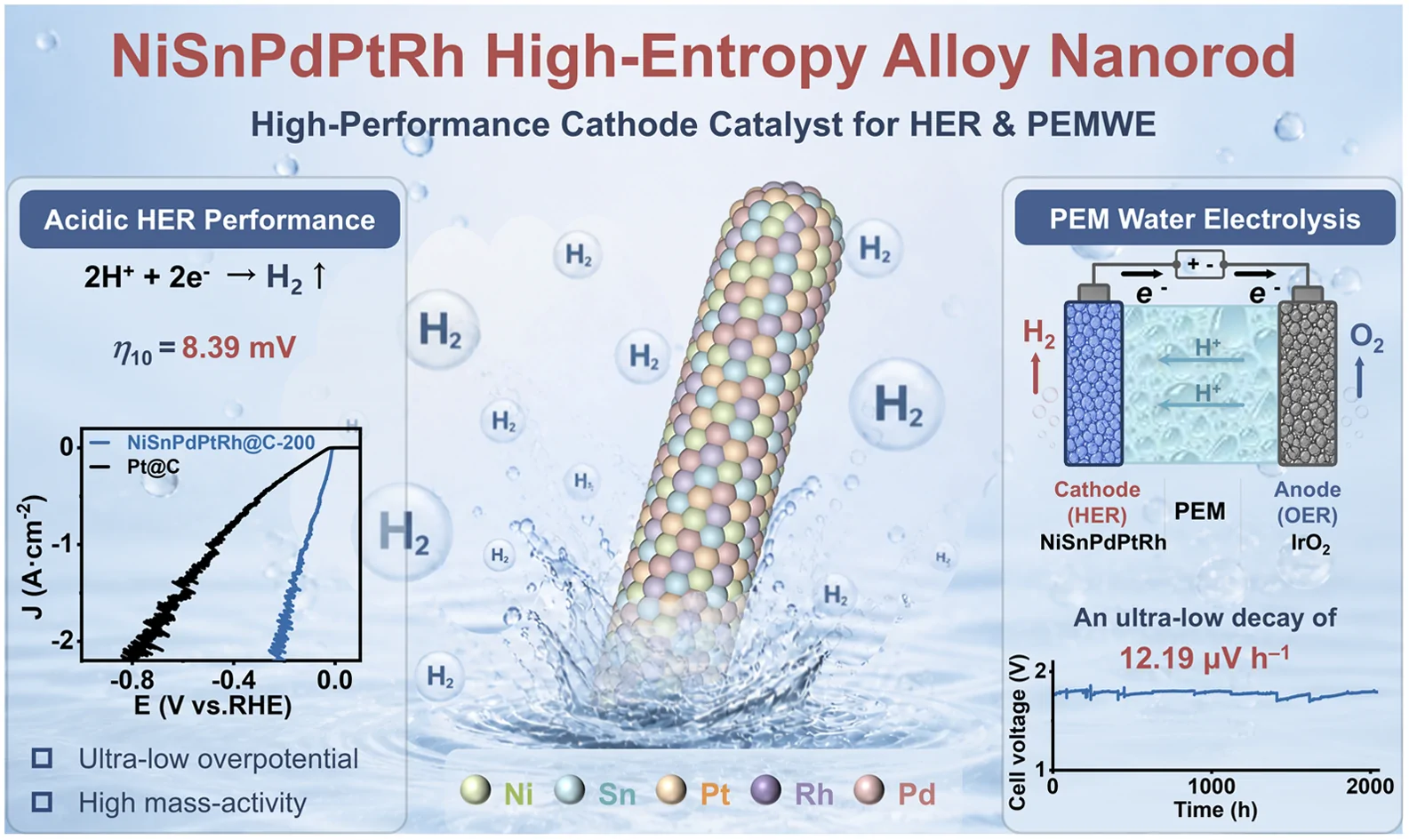

## Introduction

As one of the important technologies for realizing a sustainable hydrogen economy, water electrolysis demonstrates tremendous potential due to its environmental friendliness and process cleanliness as well as suitability for large-scale hydrogen production. However, its efficiency depends on the catalytic performance of electrocatalysts for the hydrogen evolution reaction (HER) through water splitting. Though Pt-based catalysts exhibit excellent HER performance, their widespread applications are severely restricted due to cost-effectiveness.^1–3^ Consequently, the development of highly efficient, durable, and economically viable HER catalysts remains an urgent demand in the field.^4^

Recently, high-entropy alloys (HEAs), consisting of five or more principal metals, each comprising an atomic percentage of at least 5%, have attracted considerable attention for the electrocatalytic HER.^5–9^ The potential advantages of HEAs in electrocatalysis should be ascribed to their high entropy effect, sluggish diffusion effect, lattice distortion effect, and the “cocktail effect”.^10,11^ Despite this promise, synthesizing HEA nanomaterials with uniform size, well-defined morphology, and controlled surface structure remains a challenge. Existing methods, such as carbothermal shock,^12^ electrochemical deposition,^13^ fast moving bed pyrolysis,^14^ continuous-flow reactors,^15^ sputtering deposition and electrospinning,^16^ spark plasma sintering,^17^ dealloying,^18^ liquid metal-assisted,^19^ wet chemical synthesis,^20^ microwave heating,^21–25^ a liquid-phase method,^26^ a strain engineering methodology^27^ and a high-pressure, high temperature approach,^28^ have been used to synthesize HEA nanomaterials, but a precise control over the HEA composition, morphology, and surface structure remains elusive due to the significant differences in reduction, nucleation, and growth kinetics of the multi-principal elements.^29–35^ Furthermore, research on the structure–activity relationships and catalytic mechanisms, as well as the identification of active sites of HEAs, is still insufficient, impeding the rational design of high-performance HEA catalysts.

Microwave-irradiated synthesis has been proven to be one of the rapid, facile and effective strategies for achieving metallic and alloy nanocrystals with different morphologies and surface structures due to its advantages such as fast, uniform and deep heating as well as the non-thermal effect, low cost and high energy efficiency.^36,37^ It provides a promising fast path to synthesize polymetallic HEA nanostructures with uniform morphologies by promoting simultaneous co-reduction of mixed metallic precursors within minutes. Despite recent reports on microwave-assisted syntheses of HEAs, most of them are limited to the preparation of amorphous nanoparticles by microwave heating or calcinating, while the precise control over composition and morphology such as anisotropic growth of rod-like nanostructures remains unexplored.^21–25^ Additionally, the growth mechanism of shaped HEA nanocrystals under one-step microwave irradiation has not been clarified.

Herein, we report a microwave-assisted one-pot rapid synthesis of NiSnPdPtRh HEA nanorods (NRs) with a domestic microwave oven within 180 s. The interval sampling revealed that NiSnPdPtRh NRs form *via* directional assembly of pre-formed metallic nanoclusters, followed by atomistic fusion and diffusion. After being supported on carbon and annealed at 200 °C, the resulting NiSnPdPtRh@C-200 NRs exhibited exceptional acidic HER performance: an ultra-low overpotential of 8.39 mV at 10 mA cm^−2^ as well as a very small Tafel slope of 7.92 mV dec^−1^. In proton exchange membrane water electrolysis (PEMWE) testing, the as-prepared NiSnPdPtRh@C-200 NRs display an ultra-low voltage decay of just 12.19 µV h^−1^ after operation for over 2000 h at 500 mA cm^−2^. This work not only establishes a simple, rapid and scalable synthetic strategy for shape-controlled syntheses of HEA by clarifying the microwave-irradiated growth mechanism of HEA NRs, but also lays the foundation for designing high-efficiency multielement HEA nanocatalysts with exceptional durability for industrial water electrolysis.

## Results and discussion

### Material syntheses and characterization

In a typical synthesis, a mixed solution of Ni, Sn, Pd, Pt, and Rh chloride precursors dissolved in tetraethylene glycol (TEG), in the presence an appropriate amount of polyvinylpyrrolidone (PVP) and KBr, was irradiated for 180 sec by using a modified household microwave oven (Galanz, 900 W), as shown in Fig. 1a. In the reaction system, the molar ratio of Ni/Sn/Pd/Pt/Rh/PVP/KBr was 1/2/1/1/1/20/40 and TEG acted as a reducing agent as well as a solvent. This facile microwave-enabled protocol achieves uniform co-reduction of all five metal ions in a single step.

**Fig. 1:**
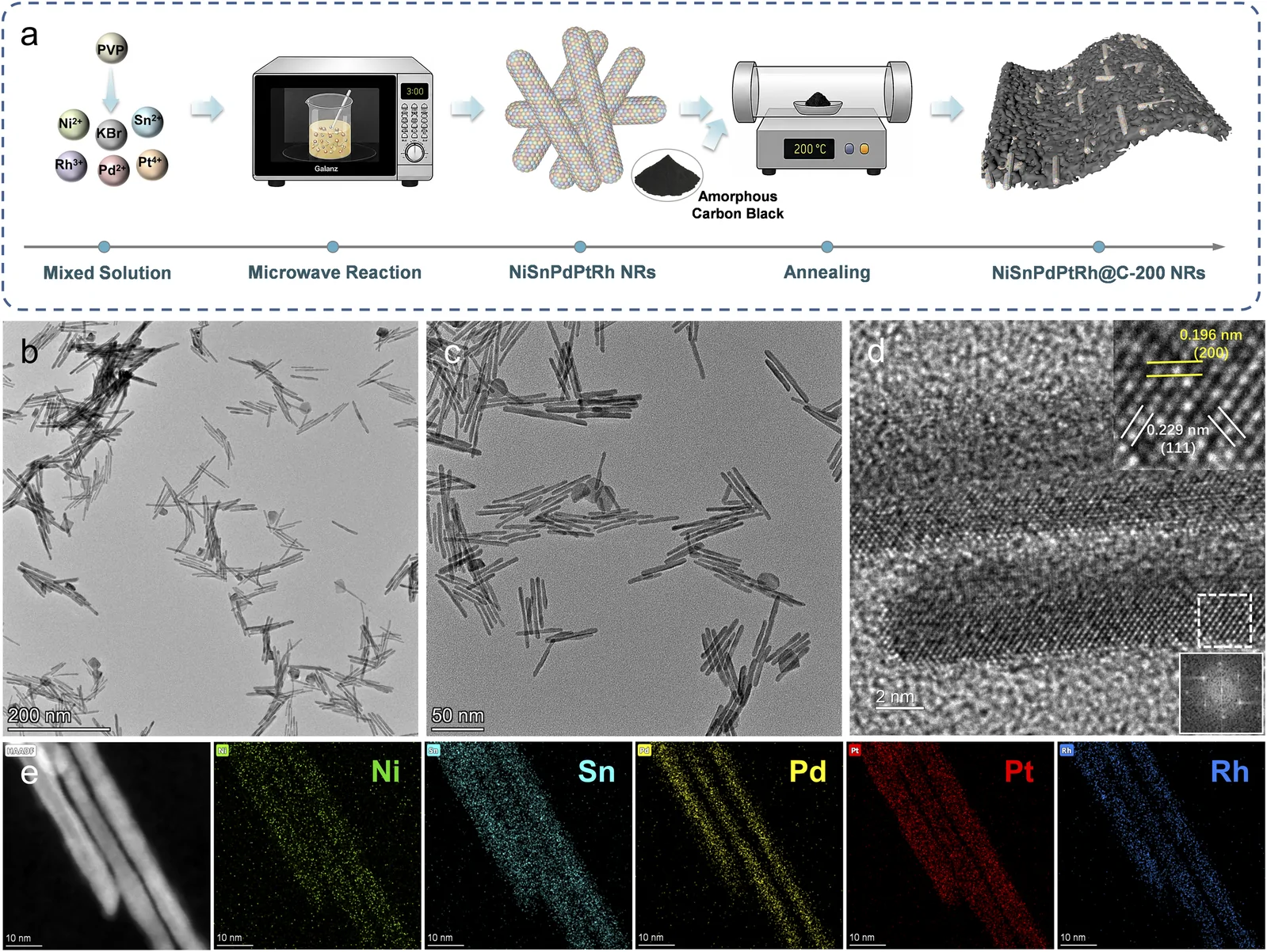
(a) Schematic of the synthesis process of NiSnPdPtRh NRs. (b and c) TEM images of the sample obtained with 900 W microwave irradiation for 180 s. (d) HRTEM and the corresponding FFT pattern of NiSnPdPtRh NRs. (e) HAADF-STEM image and the corresponding EDS mapping images of NiSnPdPtRh NRs.

The transmission electron microscopy (TEM) images (Fig. 1b and c) show that the as-synthesized HEA nanocrystals under microwave irradiation for 180 s at 900 W exhibited uniform and well-defined nanorods (NRs) with an average length of ∼42 nm, a diameter of ∼4.5 nm, and an aspect ratio of 9.3 (Fig. S1). The scanning electron microscopy (SEM) image further confirmed their nanorod feature (Fig. S2). The high-resolution TEM (HRTEM) image (Fig. 1d) shows well-resolved continuous fringes with interplanar spacings of 0.229 and 0.196 nm, which can be attributed to the (111) and (200) facets of the face-centered cubic (fcc) crystalline phase, respectively. The corresponding fast Fourier transform (FFT) image (the inset in Fig. 1d) demonstrated a single-crystalline structure with a [110] growth direction precisely along the rod axis. High-angle annular dark-field scanning transmission electron microscopy (HAADF-STEM) and the corresponding energy dispersive X-ray spectroscopy (EDS) mapping (Fig. 1e) revealed that Ni, Sn, Pd, Pt, and Rh elements were uniformly distributed throughout the whole NiSnPdPtRh NRs without phase segregation. The atomic percentages of Ni, Sn, Pd, Pt and Rh were determined to be 6.8%, 31.34%, 24.34%, 23.6% and 13.92% (Table S1) by ICP-OES measurements, which were consistent with EDS line scanning results (Fig. S3), confirming the HEA characteristics of NiSnPdPtRh NRs.

To explore the growth dynamics of NiSnPdPtRh NRs, the impact of the microwave irradiation power at 100%, 80% and 60% output (900 W, 720 W, and 540 W) at a fixed irradiation time of 180 s was first investigated. As power decreased from 900 W to 540 W, the yield gradually reduced (Table S2) and the diameter of the as-prepared NRs became significantly thinner (∼4.5 nm at 900 W *vs.* 2.96 nm at 540 W), with an increased mean length (∼42 nm at 900 W *vs.* 57.6 nm at 540 W) (Fig. S4), which in turn raised the average aspect ratio (from ∼9.3 at 900 W to ∼19.5 at 540 W). This behaviour is attributed to the power-dependent heating rate on nucleation and growth of nanorods: higher power accelerates precursor reduction and promotes faster isotropic deposition, whereas lower power slows down these processes, leading to fewer but slender nanorods. Considering both yield and morphology uniformity, 900 W was selected as the optimal power.

The time-resolved growth dynamics were then explored at 540 W. At 120 s, the products comprised sparse slender wires (∼70 nm of the length) alongside many metallic nanoclusters and some irregular particles (Fig. 2a); no product could be obtained at a shorter time. At 180 s, the number of nanowires significantly increased, while the number of metallic nanoclusters obviously decreased (Fig. 2b). With increasing irradiation time from 240 to 360 s, a large number of uniform nanowires were formed, accompanied by an increase in the yield (Table S3), while nearly no metallic nanoclusters were detected (Fig. 2c–e). These observations suggested that the formation of NiSnPdPtRh nanowires or nanorods was derived from the assembly of preformed metallic nanoclusters, in agreement with the earlier report.^38^ The mean diameter increased while mean length gradually decreased, causing the aspect ratio to fall steadily, which was consistent with Ostwald ripening at extended irradiation time (Fig. 2f). The smallest average aspect ratio (∼9.7) was observed at 360 s, which was approximately equivalent to the value observed with 900 W irradiation at 180 s, suggesting a shared endpoint driven by the same ripening mechanism. Elemental mapping and aspect ratio statistics from time-resolved sampling (Fig. S5–S9) revealed that Pd and Sn atoms dominated the rod-shaped distribution at the early stage. Over time, Rh and Pt were gradually incorporated and integrated into the alloy nanorods, followed by a decreased aspect ratio. Similar morphology evolutions with time dependence were also observed under 900 W microwave irradiation (Fig. S10). At the early reaction time (90 s), a number of slender nanowires generated along with aggregated metallic nanoclusters and occasional irregular nanoparticles (Fig. S10a), and the lower temperature and the lower yield (Table S4) implied incomplete nucleation and growth of metal nanocrystals. As the irradiation time progressed to 120 or 150 s, the uniformity and yield of the NiSnPdPtRh NRs significantly improved, followed by disappearance of nanoclusters, except for a few irregular nanocrystals (Fig. S10b, c and Table S4). However, excessive time (210 s) degraded uniformity due to over-ripening (Fig. S10d).

**Fig. 2:**
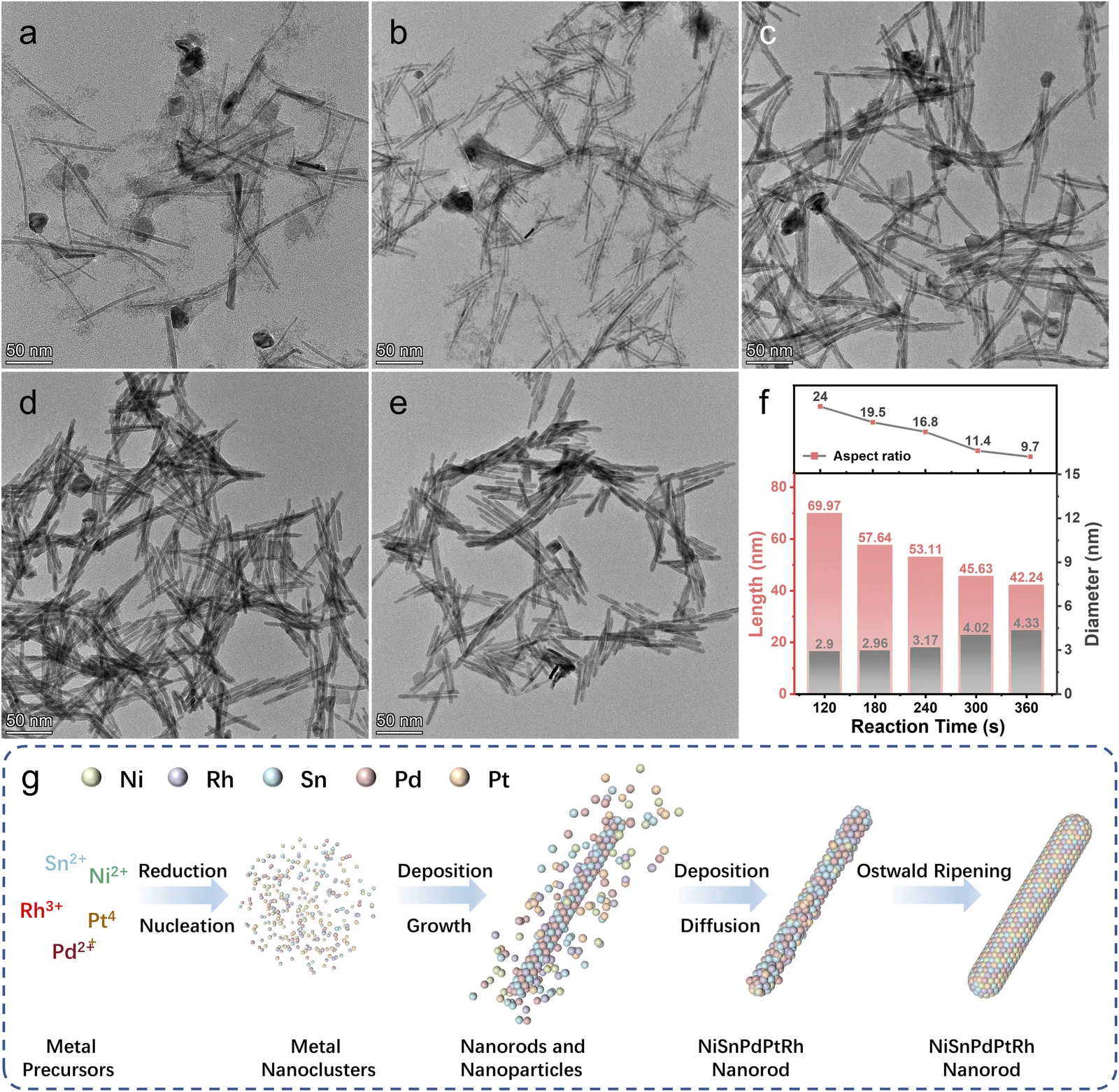
TEM images of NiSnPdPtRh NRs synthesized under microwave irradiation at 540 W for (a) 120 s, (b) 180 s, (c) 240 s, (d) 300 s and (e) 360 s, respectively. (f) The variation of the length, diameter and aspect ratio of the as-prepared NiSnPdPtRh NRs with reaction time. (g) Schematic illustration of the formation mechanism of NiSnPdPtRh NRs.

Based on these observations across various irradiation powers and reaction times, it is speculated that the nanowires likely originated from the linear assembly and growth of metallic nanoclusters obtained from preferentially reduced metal precursors, followed by atomic deposition, fusion and diffusion, as shown in the schematic illustration of nanorod formation in Fig. 2g. As the reaction time prolonged, metallic nanoclusters continually deposit, fuse and diffuse uniformly onto the nanowires, gradually thickening to form nanorods. Concurrently, Ostwald ripening may minimize the total interfacial energy, resulting in a slight shortening of the NiSnPdPtRh HEA NRs and a gradual decreasing in the total aspect ratio.

To further elucidate the role of each metal precursor in the formation of NiSnPdPtRh HEA NRs, their quaternary alloy counterparts missing one metal component were synthesized. It was found that the nanorods still formed for the quaternary counterpart alloys in the absence of Ni, Pt, or Rh (Fig. 3a–c), respectively. However, only irregularly aggregated nanoparticles without any nanorods were observed when Sn or Pd was absent (Fig. 3d and e). These results indicated that Sn and Pd played a crucial role in guiding the growth of the nanorod structure. Ternary alloy control experiments further confirmed that only SnPdRh showed well-defined and uniformly distributed slim nanorods, whereas NiSnPd or SnPdPt as well as NiPtRh alloys presented no nanorod structure except amorphous nanoparticles (Fig. S11). These observations suggest that Sn and Pd may be indispensable for directing the formation of nanorod structures, while Ni and Pt can be integrated later without disrupting the morphology. These results are consistent with those reflected in Fig. S5–S9, further supporting the proposed formation mechanism.

**Fig. 3:**
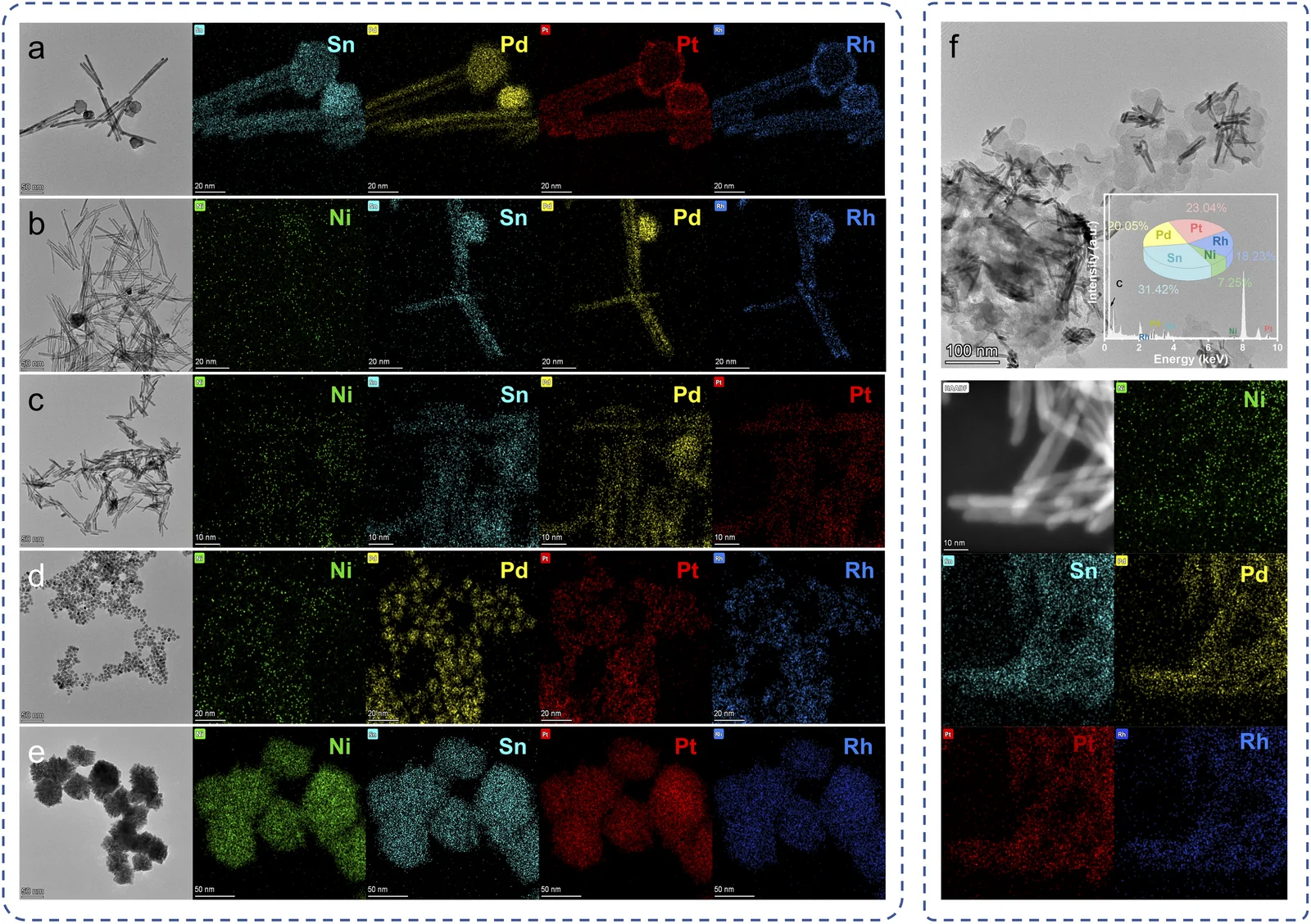
TEM and HAADF-STEM images and the corresponding EDS mapping of quaternary alloys in the absence of (a) Ni, (b) Pt, (c) Rh, (d) Sn, or (e) Pd. (f) TEM and HAADF-STEM images and the corresponding EDS mapping of NiSnPdPtRh@C-200 (the inset shows the atomic percentage of NiSnPdPtRh@C-200 NRs measured by EDS mapping).

Furthermore, control experiments clarified the effect of KBr on NiSnPdPtRh NR formation (Fig. S12). In the absence of KBr (0 mmol), only irregular nanoparticles formed (Fig. S12a). With 0.1 mmol KBr, longer and thicker NRs with rough surfaces accompanied by a few square nanosheets were generated (Fig. S12b), indicating an incompletely controlled growth. In the presence of optimal 0.2 mmol KBr as shown in the typical experiment, well-defined NiSnPdPtRh NRs with a minimum amount of irregularly nanoparticles were produced (Fig. S12c and Fig. 1a). Excess KBr (0.3 mmol) resulted in slimmer NRs and rectangle nanosheets (Fig. S12d). Thus, an appropriate amount of KBr is essential for the generation of uniform NiSnPdPtRh NRs. Equimolar KI, KCl or NaBr instead of KBr produced mostly irregular nanoparticles (Fig. S13), suggesting a pivotal role of Br^−^ anions in directing the linear assembly of metallic nanoclusters into nanorods by regulating the selective adsorption of Br^−^ ions and the deposition of metal atoms. Possibly, the preferential adsorption of Br^−^ ions onto the (111) and (100) facets directs the [110] oriented growth of rod-like structures, while the ionic radius and moderate electronegativity of Br^−^ ions further enhance the formation of uniform nanorod through a capping effect. In contrast, I^−^ ions further suppress the deposition of metal atoms on the (111) and (100) facets due to their stronger adsorption affinity and larger ionic radius, leading to the formation of higher aspect ratio nanorods or even thinner nanowires, whereas the weaker adsorption of Cl^−^ ions facilitates more convenient deposition of metal atoms, resulting in thicker rods with lower aspect ratios and less pronounced anisotropy.

Additionally, PVP serves as both a stabilizing agent and a shape-directing agent of HEA NRs. As shown in Fig. S14a, in the absence of PVP, no nanorods were formed; instead, severely agglomerated irregular particles were obtained. With a small amount of PVP (0.05 mmol), uniform, high aspect ratio nanorods were produced (Fig. S14b), though with a lower yield compared with the optimum case (0.1 mmol). Nevertheless, excessive PVP (0.15 mmol) resulted in poor uniformity and more irregular nanoparticles (Fig. S14c). Thus, the optimal dosage of PVP is critical for the creation of high-yield uniform NiSnPdPtRh NRs.

The solvent had a significant impact on the formation of NiSnPdPtRh NRs under microwave irradiation. As shown in Fig. S15, no nanorods but irregular nanoparticles were produced using ethylene glycol (EG) or glycerol (GLY) instead of TEG, while a small amount of nonuniform nanorods were obtained in triethylene glycol (TriEG). These polyols served as both solvents and reducing agents and demonstrated stronger reducibility and faster nucleation kinetics than TEG in this reaction system. The faster reduction rate resulting from EG, GLY and TriEG may lead to a rapid generation of crystal nuclei, so that it is unfavourable for the directional growth of NiSnPdPtRh NRs.

Moreover, the influence of metal precursor molar ratios on the morphology of the HEAs was also investigated. It was found that an increase in the feeding ratio of the Pt or Rh precursor resulted in a decrease of NiSnPdPtRh NRs but a significant increase of irregular nanoparticles, though with a higher atomic percentage of Pt or Rh in the product (Fig. S16, S17 and Table S5), while a higher feeding ratio of the Pd precursor brought about elongated NiSnPdPtRh NRs along with a significant increase of Pd atomic percentage and an obvious decrease of atomic percentages of Pt and Rh (Fig. S18 and Table S5). Notably, an increase in the molar ratio of the Ni precursor led to a decrease in the Ni percentage in the final product, but the nanorod structure was still maintained (Fig. S19 and Table S5). In addition, whether increasing or reducing the dosage of the Sn precursor relatively to the optimal case, the uniformity of NiSnPdPtRh NRs declined (Fig. S20 and Table S5). Obviously, a precise control of the metal precursor molar ratio was essential for the generation of uniform and well-defined NiSnPdPtRh HEA NRs.

To evaluate the electrochemical performance, NiSnPdPtRh NRs were loaded onto carbon powder and thermally annealed at 180, 200, 300 and 400 °C in an air atmosphere to remove surface PVP, and the as-prepared samples were denoted as NiSnPdPtRh@C-*T* NRs (in which *T* represented the annealing temperature). It was found that direct calcination of the unloaded NiSnPdPtRh NRs at 200 °C for 2 h resulted in severe fracture and agglomeration (NiSnPdPtRh-200 NRs, Fig. S21a). The carbon-supported NiSnPdPtRh NRs remained well dispersed (NiSnPdPtRh@C NRs, Fig. S21b). Moreover, the morphologies of the NiSnPdPtRh NRs were well maintained in the carbon-supported samples annealed at 180 and 200 °C (NiSnPdPtRh@C-180 NRs and NiSnPdPtRh@C-200 NRs, respectively; Fig. S21c and d), while obvious breakage and aggregation were observed for those annealed at 300 and 400 °C (NiSnPdPtRh@C-300 NRs and NiSnPdPtRh@C-400 NRs, respectively; Fig. S21e and f). Additionally, EDS mapping (Fig. 3f) and ICP-OES (Table S6) analyses revealed that the atomic percentages of all the elements in NiSnPdPtRh@C-200 NRs were highly consistent with those in NiSnPdPtRh NRs, and the total metal loading in NiSnPdPtRh@C-200 NRs was ∼39%.

Fig. 4a shows the XRD patterns of the typical NiSnPdPtRh NRs and NiSnPdPtRh@C-200 NRs. The characteristic peaks at 39.50°, 45.19°, 66.51° and 80.205° can be well assigned to the (111), (200), (220) and (311) facets, respectively, further confirming their fcc alloy structure. After being loaded onto carbon powder and thermally treated at 200 °C, the XRD diffraction pattern remained unchanged, but the diffraction peaks became sharper due to the improved crystallinity, implying an exfoliation of PVP. The surface elemental composition and chemical states of NiSnPdPtRh NRs were identified by XPS analysis. Pd 3d XPS spectra (Fig. 4b) could be mainly assigned to Pd^0^ (Pd^0^ 3d_5/2_ and Pd^0^ 3d_3/2_ at 335.67 and 340.98 eV, respectively). Sn 3d XPS spectra (Fig. 4c) showed Sn^0^ 3d_5/2_ (485.1 eV) and Sn^0^ 3d_3/2_ (493.56 eV) as well as Sn^2+^ 3d_5/2_ (486.47 eV) and Sn^2+^ 3d_3/2_ (494.90 eV), indicating the presence of Sn^0^ and Sn^2+^ species. The Ni 2p spectrum (Fig. S22a) revealed the existence of Ni^0^ (852.42 and 872.72 eV for Ni^0^ 2P_3/2_ and Ni^0^ 2P_1/2_, respectively) and Ni^2+^ (855.64 and 873.69 eV for Ni^2+^ 2P_3/2_ and Ni^2+^ 2P_1/2_, respectively). The oxidation of surface Ni following exposure to air should be ascribed to its strong reducibility. Pt 4f orbitals were discerned at binding energies of 71.17 (Pt^0^ 4f_7/2_) and 74.48 (Pt^0^ 4f_5/2_) eV (Fig. S22b), indicating its metallic state. The Rh 3d XPS spectrum (Fig. S22c) showed two strong peaks at 306.52 eV (Rh^0^ 3d_5/2_) and 311.26 eV (Rh^0^ 3d_3/2_), respectively, along with Rh^3+^ electronic states at 307.51 (Rh^3+^ 3d_5/2_) and 312.36 eV (Rh^3+^ 3d_3/2_), while the two additional peaks at 313.86 and 315.62 eV in the Rh 3d region were identified as Pt^0^ 4d_5/2_ and Pt^2+^ 4d_5/2_, respectively. The XPS observations for NiSnPdPtRh@C-200 NRs (Fig. S22d–h and Table S7) showed that all the binding energies of Ni^0^ 2p, Pt^0^ 4f, Rh^0^ 3d, Pd^0^ 3d and Sn^0^ 3d exhibited positive shifts of 0.5 to 0.74 eV compared with those of the pristine NiSnPdPtRh NRs, respectively, which might be caused by the surface oxidation or the interface interaction between metal atoms and carbon supports.^39–41^

**Fig. 4:**
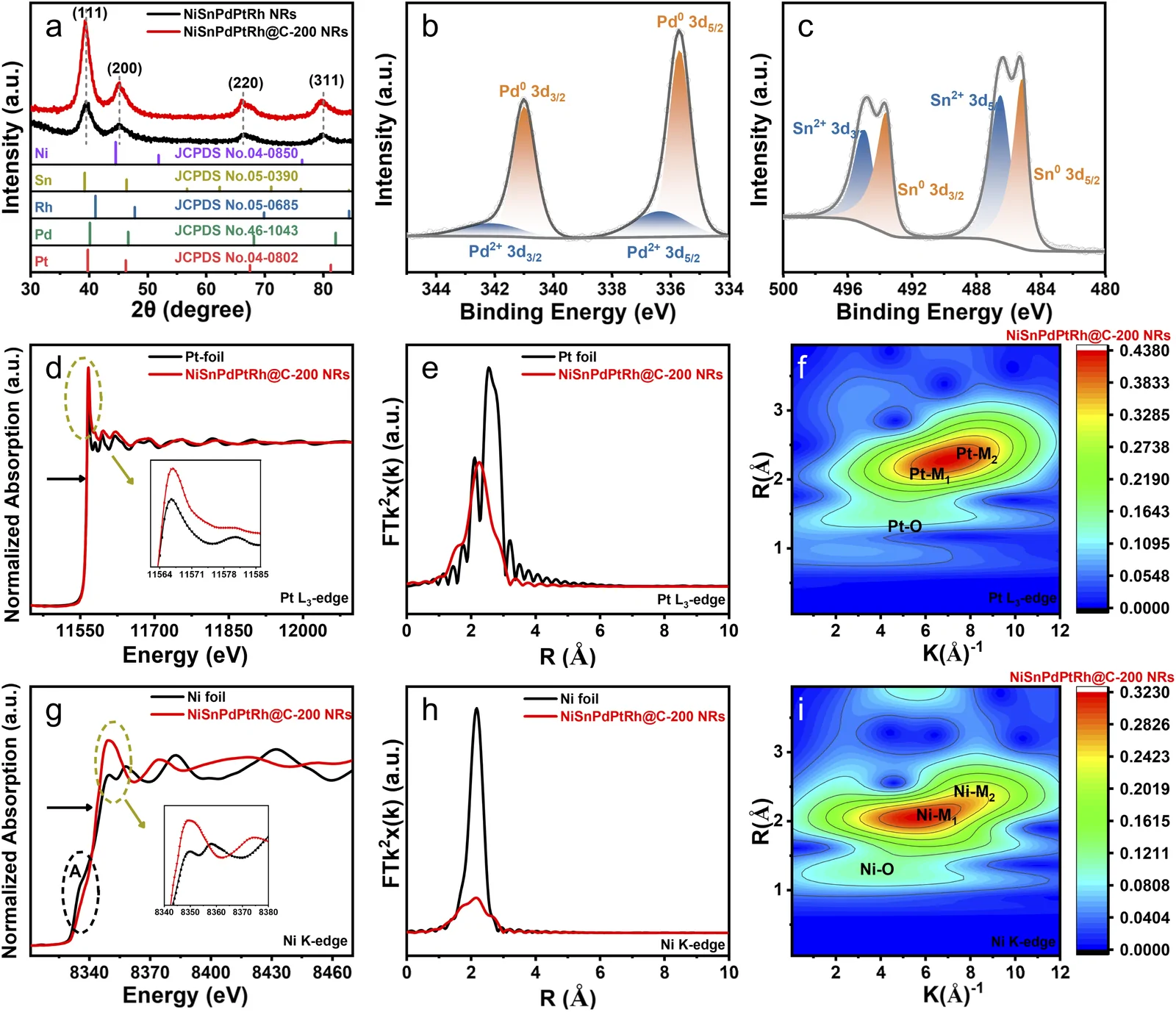
Composition and structure analyses. (a) XRD patterns of NiSnPdPtRh NRs and NiSnPdPtRh@C-200 NRs. XPS spectra of (b) Pd 3d and (c) Sn 3d regions of NiSnPdPtRh NRs. XANES spectra of the (d) Pt L_3_-edge and (g) Ni K-edge of NiSnPdPtRh@C-200 NRs. The insets in panels (d) and (g) are enlarged views of the white lines. Fourier-transformed EXAFS spectra of the (e) Pt L_3_-edge in R space of Pt-foil and NiSnPdPtRh@C-200 NRs and (h) Ni K-edge of Ni foil and NiSnPdPtRh@C-200 NRs. The wavelet transform (WT) plots for the (f) Pt L_3_-edge and (i) Ni K-edge of NiSnPdPtRh@C-200 NRs.

The atomic coordination environment of NiSnPdPtRh@C-200 NRs was inspected using X-ray absorption near-edge structure (XANES) and extended X-ray absorption fine structure (EXAFS) measurements at the synchrotron radiation facility (Fig. 4d–i, S23 and S24). The XANES spectrum of NiSnPdPtRh@C-200 NRs showed that the Pt L_3_-edge inflection point of NiSnPdPtRh@C-200 NRs was coincident with that of Pt foil, as shown in Fig. 4d (where the black arrow is pointing), indicating that Pt primarily existed in a metallic state, while the higher white-line peak than that of Pt foil reflected significant changes of the Pt 5d orbitals and the d-orbital energy level splitting, suggesting strong d–d electronic interactions between Pt and other component metals.^42–44^ Similarly, the Ni K-edge displayed a pre-edge positive shift (labelled by A) and a significantly higher white-line peak than that of Ni foil, indicating its partial oxidation (Ni^*δ*+^) (Fig. 4g), which should be ascribed to the strong intermetallic interactions as well as electron transfer from Ni to other component metals in NiSnPdPtRh@C-200 NRs.^45^

Fourier transformed EXAFS (FT-EXAFS) and wavelet transform (WT) analyses can provide more detailed local structural information. The FT-EXAFS spectra for the Pt L_3_-edge (Fig. 4e) and the corresponding fitting results demonstrated that the local coordination of Pt was predominantly composed of Pt–M_1_ and Pt–M_2_, as well as small amount of Pt–O (Fig. 4f and Table S8), indicating two kinds of bonding environments. The coordination numbers of Pt–Ni and Pt–Pt were 3.1 and 5.8, respectively, and the corresponding bond lengths were 2.60 and 2.73 Å (Fig. S23 and Table S8), which were shorter than that of Pt–Pt in metallic Pt foil (2.76 Å). The reduced bond distances should be ascribed to the strong electron localization between Pt and other metal atoms as well as the changes in the coordination environment resulting from alloying, which induced compressive strain in Pt atoms in the as-formed NiSnPdPtRh HEA NRs. WT-EXAFS analyses for the Pt L_3_-edge of NiSnPdPtRh@C-200 NRs showed obvious multi-coordination features of Pt atoms, presenting the disordered local structure of the HEAs and the complex electronic couplings among multiple metals.

Similarly, the FT-EXAFS spectra for the Ni K-edge (Fig. 4h and Table S9) and the corresponding WT analysis (Fig. 4i) showed the coordination shell of Ni–M_1_, Ni–M_2_, and Ni–O, in accordance with the EXAFS results at the Pt L_3_-edge. The bond lengths of Ni–M(Pd/Rh) and Ni–Pt, with the same coordination number of 4.3, were 2.59 and 2.64 Å (Fig. S24 and Table S9), respectively. These Ni–Ni and Ni–Pt bond lengths were slightly longer than the Ni–Ni bond length in Ni foil (2.48 Å) due to the larger atomic radii of the other metal atoms, which induced extensive strain at Ni sites within the NiSnPdPtRh HEA NRs. The two coordination bonds of Ni shown by WT analyses confirmed the complex coordination structure of Ni formed in alloy phases with other component metal elements, with a lower Ni–O coordination number, revealing the predominant metallic states of Ni in NiSnPdPtRh@C-200 NRs.

### Performance of electrocatalytic HER

The electrochemical HER performances were evaluated using a typical three-electrode system in 0.5 M H_2_SO_4_ solution at room temperature. It was found that NiSnPdPtRh@C-200 NRs required an ultra-low overpotential (*η*_10_) of only 8.39 mV at a current density of 10 mA cm^−2^ (Fig. 5a), which was remarkably lower than those of control samples such as NiSnPdPtRh-200 NRs (12.68 mV), NiSnPdPtRh@C NRs (25.79 mV), NiSnPdPtRh@C-180 NRs (15.41 mV), and NiSnPdPtRh@C-300 NRs (10.15 mV), while the pure carbon supports did not exhibit HER catalytic activity under the same conditions. In addition, NiSnPdPtRh@C-200 NRs demonstrated the lowest Tafel slope (7.92 mV dec^−1^) (Fig. 5b and c) and the lowest interfacial charge transfer resistance (0.446 Ω) among all the control samples (Fig. S25), indicating superior intrinsic catalytic kinetics to those of all other samples. Thus, it can be seen that NiSnPdPtRh@C-200 NRs exhibit excellent performance in acidic HER.

**Fig. 5:**
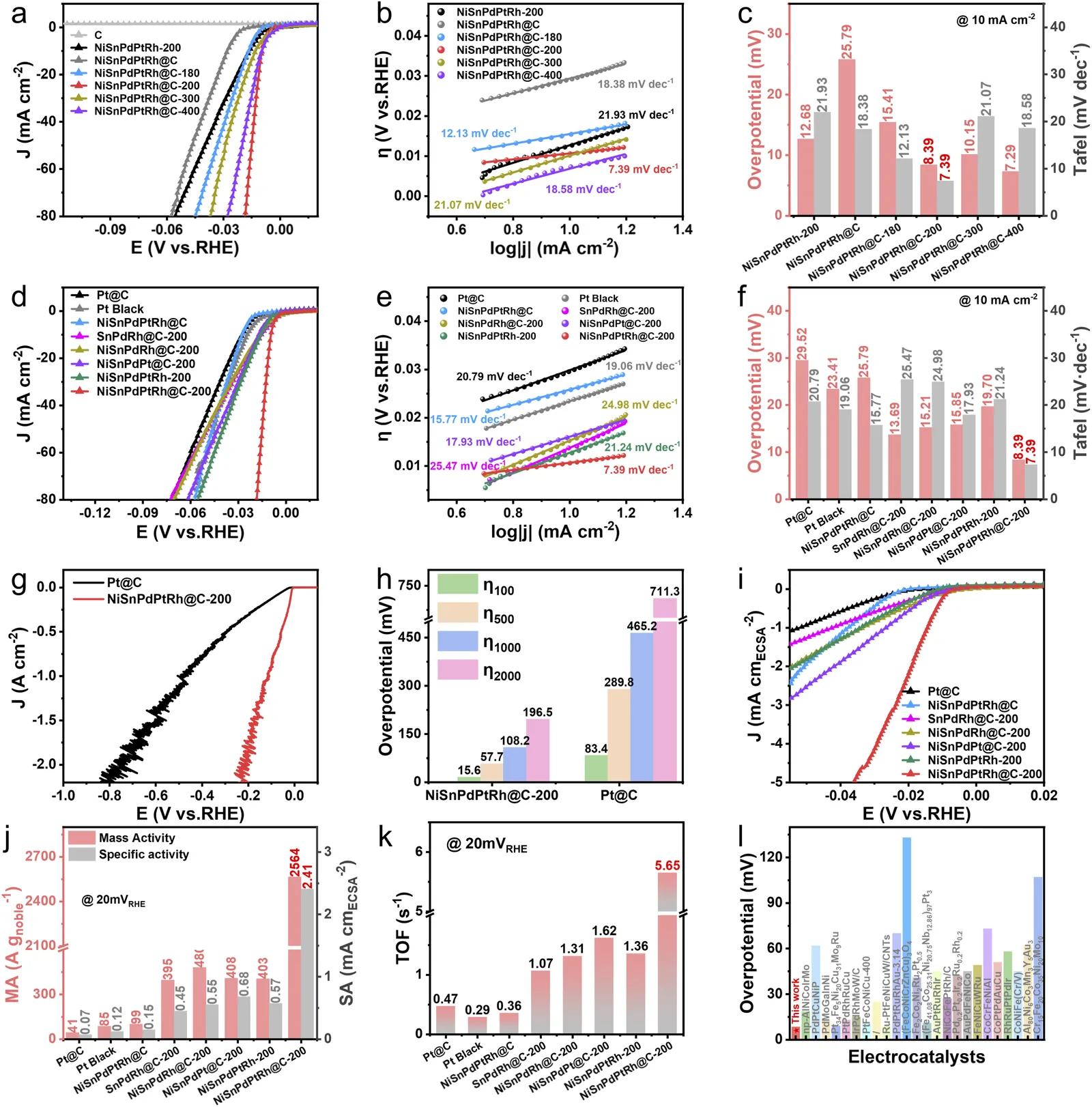
HER performances of NiSnPdPtRh@C-*T* (*T* = 180, 200, 300, 400) NRs and the control samples in 0.5 M H_2_SO_4_: (a) polarization curves, (b) Tafel slope curves, and (c) overpotential (*η*_10_) and Tafel slopes. HER performance of NiSnPdPtRh@C-200 and the control samples in 0.5 M H_2_SO_4_: (d) polarization curves, (e) Tafel slope curves, (f) overpotential (*η*_10_) and Tafel slopes, (g) polarization curves of NiSnPdPtRh@C-200 NRs and commercial Pt@C at high current densities, (h) comparisons of overpotentials at 100, 500, 1000 and 2000 mA cm^−2^ between NiSnPdPtRh@C-200 NRs and Pt@C, (i) ECSA-normalized HER polarization curves, (j) the mass activity and specific activity at 20 mV *vs.* RHE, (k) TOF values at 20 mV *vs.* RHE, (l) comparison of the overpotentials (*η*_10_) of NiSnPdPtRh@C-200 NRs with most of the reported HEA catalysts.

To explore the role of individual metal components in the HER performance of the NiSnPdPtRh NRs, a series of quaternary and ternary alloy nanorods with carbon-supported thermal treatment such as SnPdRh@C-200 NRs, NiSnPdPt@C-200 NRs and NiSnPdRh@C-200 NRs were prepared (Fig. S26a–c), respectively, and their HER performances were evaluated equally (Fig. 5d–f and S27). Among all the tested catalysts, NiSnPdPtRh@C-200 NRs exhibited the lowest overpotential (*η*_10_ = 8.39 mV) and Tafel slope (7.92 mV dec^−1^), outperforming NiSnPdRh@C-200 NRs (15.21 mV), NiSnPdPt@C-200 NRs (15.85 mV), SnPdRh@C-180 NRs (13.69 mV), and even commercial Pt black (23.41 mV) and Pt@C (29.52 mV), demonstrating their exceptional HER activity in acidic medium.

Notably, the NiSnPdPtRh@C-200 NRs exhibited superior catalytic performance at elevated current densities (Fig. 5g). As the current density increased, the required overpotential also demonstrated its distinct advantage (Fig. 5h). As can be seen, an overpotential of only 15.6 mV was required even at 100 mA cm^−2^, which was obviously lower than that of commercial Pt@C (83.4 mV) at the same current density. In particular, NiSnPdPtRh@C-200 NRs can achieve 1000 and 2000 mA cm^−2^ with overpotentials of merely 108.2 and 196.5 mV, respectively, which were remarkably lower than that of Pt@C (465.2 and 711.3 mV, respectively) under the identical conditions, displaying far superior performance to commercial Pt@C at industrial-level current densities. Compared with Pt@C, the substantial decrease in overpotential underscores the exceptional capability of the NiSnPdPtRh HEA NRs to drastically enhance reaction kinetics under high mass-transport conditions due to their unique electronic structure and multi-site synergy.

Furthermore, the electrochemical active surface area (ECSA) and turnover frequency (TOF) values of all the samples were determined *via* the hydrogen adsorption–desorption method. The ECSA of NiSnPdPtRh@C-200 NRs reached 68.38 m^2^ g_metal_^−1^ (Fig. S28 and S29). After ECSA normalization, NiSnPdPtRh@C-200 NRs displayed the best catalytic performance (Fig. 5i). At 20 mV, NiSnPdPtRh@C-200 NRs exhibited the highest mass activity (normalized by noble metal mass) of 2564 A g_noble_^−1^ and a specific activity of 2.41 mA cm_ECSA_^−2^ (Fig. 5j), corresponding to the largest TOF value of 5.65 s^−1^ (Fig. 5k and S30). When compared with all the control multi-metallic nanorods, NiSnPdPtRh@C-200 NRs also showed the lowest electrochemical impedance of only 0.45 Ω (Fig. S31), indicating superior electron-transport capability, which should be attributed to their unique multi-elemental composition and nanorod structure. Compared with the reported HEA catalysts, NiSnPdPtRh@C-200 NRs exhibited superior performance owing to their ultra-low overpotential and Tafel slope, ranking among the high-performance HER electrocatalysts to date (Fig. 5l and Table S10).

The long-term HER stability of NiSnPdPtRh@C-200 NRs was evaluated using chronoamperometry (CA) and an accelerated durability test (ADT) in 0.5 M H_2_SO_4_. The CA curve for 50 h showed almost negligible current decay at an overpotential (*η*_20_) of 10.57 mV at 20 mA cm^−2^ (Fig. S32a). Meanwhile, after a 50 h long-term stability test, the overpotential (*η*_20_) at 20 mA cm^−2^ with a negative shift of only 0.1 mV relative to the initial value was observed (Fig. S32b). Moreover, a similar CA result for an overpotential (*η*_500_) of 57.7 mV at 500 mA cm^−2^ was also observed for 100 h with a negative shift of only 8.4 mV relative to the initial value, as shown in Fig. 6a, and the structure and elemental contents of NiSnPdPtRh HEA NRs remained unchanged (as shown in the insets of Fig. 6a and S33). The faradaic efficiency (FE) for H_2_ evolution on NiSnPdPtRh@C-200 NRs was calculated to be 99.15% (Fig. S34), confirming that the measured current was entirely derived from the HER without interference from side reactions. These results demonstrated that NiSnPdPtRh@C-200 NRs possessed superior durability and excellent stability under acidic HER conditions, which may be attributed to the sluggish atomic diffusion effect in NiSnPdPtRh HEA NRs.

**Fig. 6:**
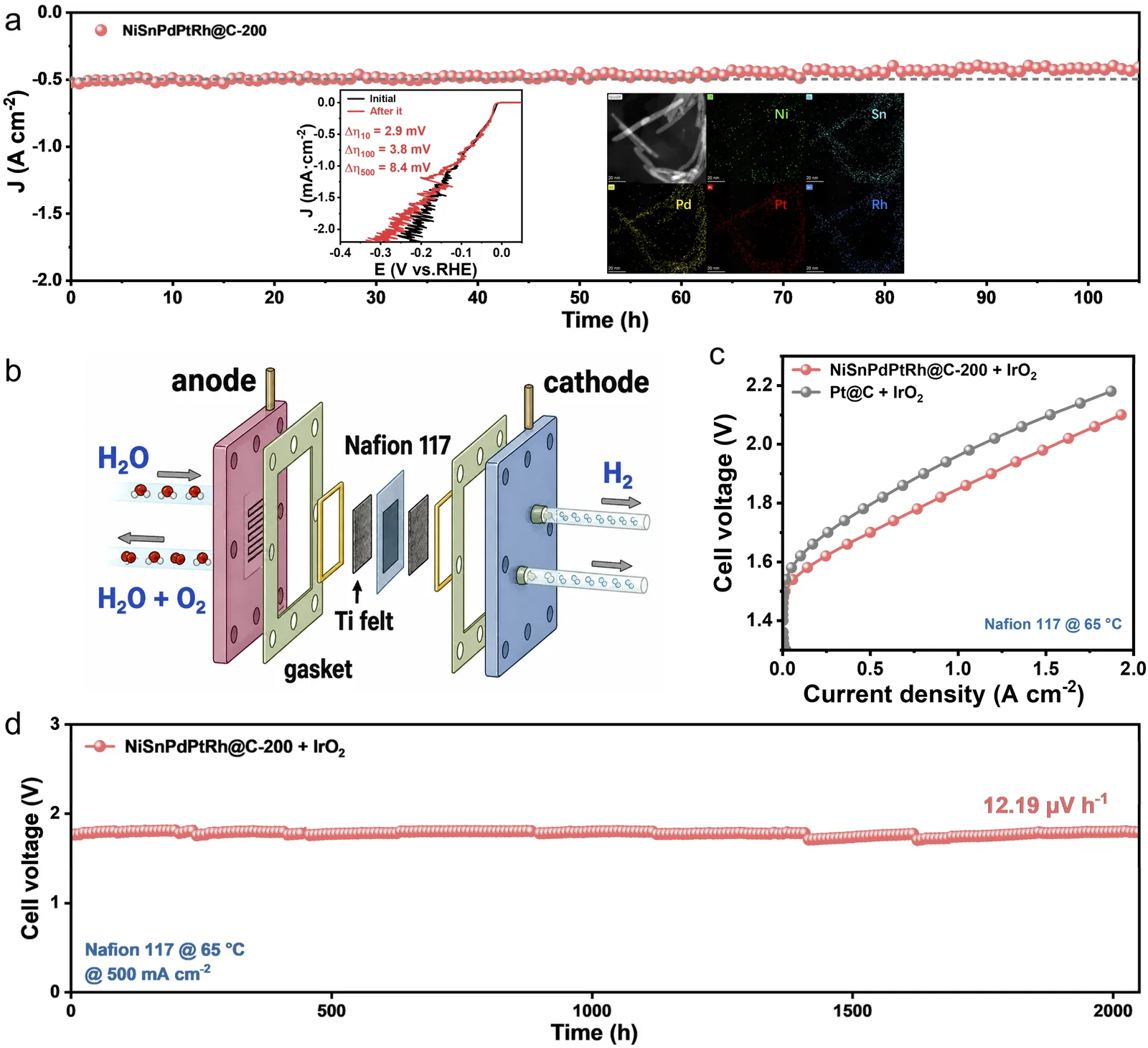
Electrochemical stability and PEMWE performance. (a) Long-term stability test of NiSnPdPtRh@C-200 NRs at −0.057 V *vs.* RHE. The insets in (a) show the polarization curves for ADT before and after the 100 hour CA test (left) as well as HAADF-STEM images and EDS mapping after the 100 hour test (right), respectively. (b) Schematic diagram of a PEMWE. (c) Polarization curves of PEMWE at 65 °C. (d) Chronopotentiometry (CP) curve of PEMWE at 500 mA cm^−2^@65 °C.

To further assess the practical application potential of NiSnPdPtRh@C-200 NRs as an acidic HER catalyst, its electrochemical performance was investigated in a PEMWE setup (Fig. 6b) by loading 0.3 mg_Pt_ cm^−2^ of the catalyst on Nafion 117, benchmarked against commercial Pt@C at the same loading. The polarization curves (Fig. 6c) revealed that NiSnPdPtRh@C-200 NRs exhibited a low cell voltage of merely 1.85 and 1.98 V at 1.0 and 1.5 A cm^−2^, respectively, surpassing Pt@C under the same conditions. When employed in a PEMWE at a constant current density of 500 mA cm^−2^, NiSnPdPtRh@C-200 NRs demonstrated an extremely low voltage decay of just 12.19 µV h^−1^ after a continuous and stable operation of 2000 h (Fig. 6d). These findings confirm the excellent catalytic activity and electrochemical durability of NiSnPdPtRh@C-200 NRs as a high-performance PEMWE cathode catalyst, suggesting their great potential for large-scale hydrogen production *via* direct water electrolysis.

### Insights into the mechanism of the HER

Furthermore, density functional theory (DFT) calculations were carried out to get insight into the catalytic mechanism for the excellent acidic HER performance of NiSnPdPtRh@C-200 NRs by constructing a substrate-free alloy surface model, as the carbon support makes no contribution to catalytic activity, followed by a systematic analysis of its electronic structure and hydrogen adsorption behaviour (Fig. 7a and S35). The total density of states (TDOS) and the projected density of states (PDOS) of NiSnPdPtRh@C-200 NRs extend beyond the Fermi level (*E*_f_), indicating their prominent metallic nature (Fig. 7b). This electronic structure suggests that NiSnPdPtRh NRs possess strong electronic conductivity, which facilitates charge transfer during the HER process. The TDOS is predominantly contributed by the d orbitals of Ni, Pd, Pt and Rh. PDOS further reveals that Sn, Pd-4d and Rh-4d orbitals reside deeper away from *E*_f_, while Ni-3d and Pt-5d orbitals are located closer to *E*_f_, which facilitate rapid electron excitation or transfer with low energy requirements for active involvement in the HER. These results highlight the critical role of Ni and Pt in improving catalytic kinetics.

**Fig. 7:**
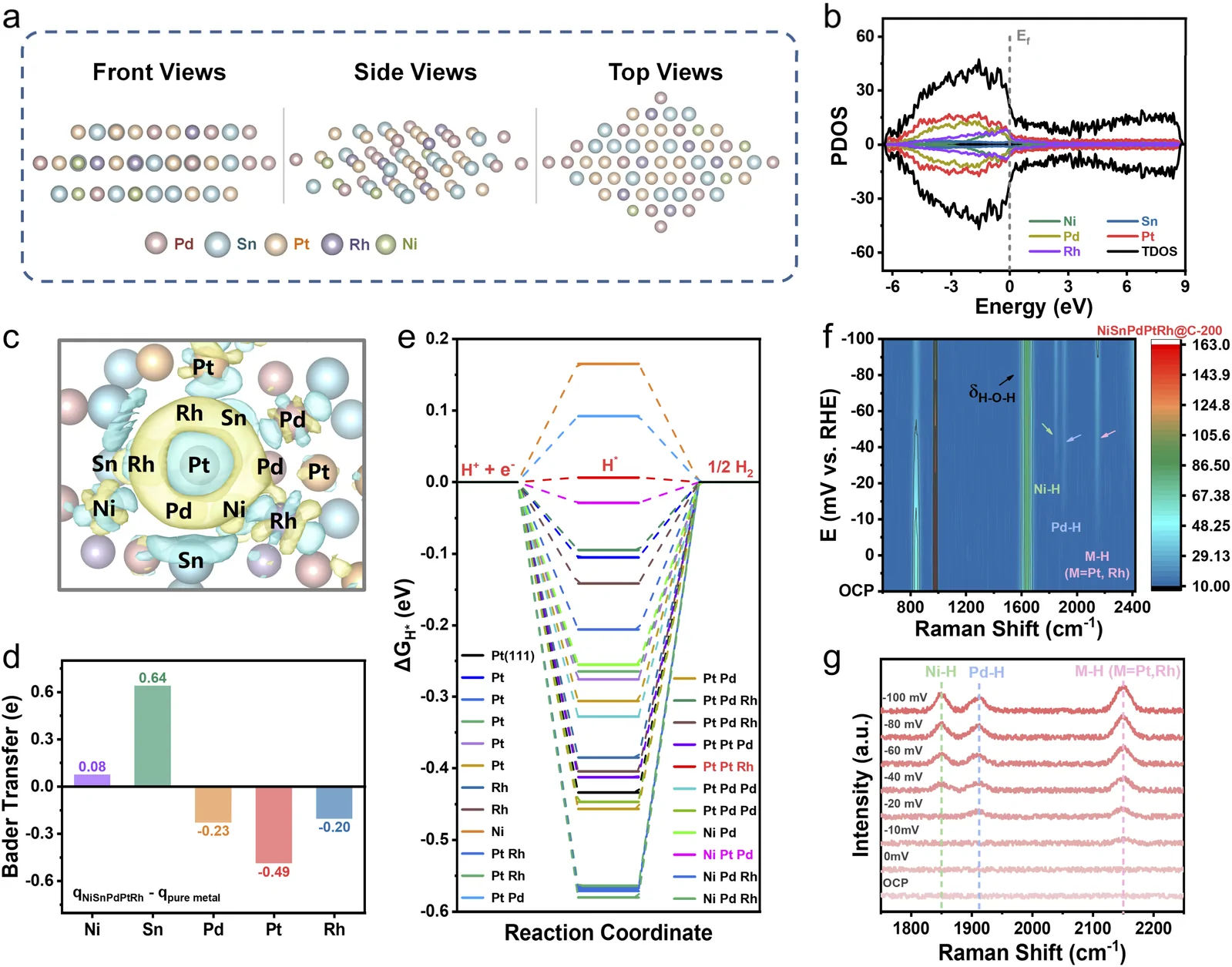
DFT calculations and SERS tests. (a) The structural model of NiSnPdPtRh NRs. (b) TDOS and PDOS spectra. (c) Local differential charge density map at the Pt sites. (d) Quantitative Bader charge analysis results for NiSnPdPtRh NRs. (e) Gibbs free energy profile of H* adsorption on NiSnPdPtRh HEA NRs. (f) and (g) *In situ* SERS spectra of NiSnPdPtRh@C-200 NRs during acidic HER at different applied potentials.

The qualitative and quantitative analyses were conducted using differential charge density and Bader charge calculations to further elucidate the local electronic interactions among multiple metallic elements. The differential charge density shown in Fig. 7c visually reveals the electron redistribution phenomenon on the alloy surface, showing distinct spatial polarization of electron clouds between different elements. Quantitative Bader charge analyses (Fig. 7d) further confirm significant charge transfer within NiSnPdPtRh HEA NRs. As it can be seen, Sn exhibits a significant positive charge (+0.64 e), indicating its primary role as an electron donor, while Pt displays the strongest negative charges (−0.49 e), along with negatively charged Rh (−0.20 e) and Pd (−0.23 e), acting as the dominant electron acceptors. Additionally, the net charge of Ni (+0.08 e) is close to zero. As a result, a large number of electrons are enriched towards Pt and other metals, which would serve as active centres. These prominent local charge transfers in NiSnPdPtRh HEA NRs effectively regulate the surface electronic structures of the multi-metallic components, thereby directly determining the thermodynamics of hydrogen adsorption on the catalyst surfaces. Therefore, as a key parameter for HER activity, the hydrogen adsorption Gibbs free energy (Δ*G*_H*_) was investigated. As shown in Fig. 7e, the representative hollow sites of Pt–Pt–Rh and Ni–Pt–Pd in NiSnPdPtRh HEA NRs exhibit Δ*G*_H*_ values close to 0 eV (Table S11), indicating thermodynamically favourable H* adsorption/desorption behaviour. These results suggest that these specific multi-metallic hollow sites act as the synergistic active centres for the HER, effectively coupling the optimized hydrogen binding energy with rapid charge transfer to realize enhanced catalytic efficiency.^45^

Additionally, *in situ* surface-enhanced Raman spectroscopy (SERS) was used to further clarify the catalytic mechanism of NiSnPdPtRh@C-200 NRs for the acidic HER at various potentials. The spectral sequences in Fig. 7f, g and S36 reveal a dynamic surface activation. The prominent feature at approximately 1640 cm^−1^ can be assigned to the typical H–O–H bending vibration of water molecules in the electrolyte, whereas the characteristic D band (∼1350 cm^−1^) and G band (∼1580 cm^−1^) of the carbon material were not detected. This may be attributed to the following reasons: (1) an extremely low laser power was adopted during *in situ* SERS measurements to avoid local heating; (2) the *in situ* SERS enhancement effect is confined to the plasmonic hot spots generated on the high-entropy alloy surfaces and not imposed on the carbon substrates; (3) carbon black is covered by nanorods and separated by a layer of electrolyte, resulting in further attenuation of its inherent weak signal.

Initially, at OCP and 0 mV, no vibrational signals were detected in the range of 1800–2200 cm^−1^, signifying no hydrogen adsorption. At −20 mV, the appearance of a broad band near 2150 cm^−1^ corresponds to H adsorbed on Pt/Rh top sites, identifying them as the initial active centres.^44^ With the potential driven further to −40 mV and more negative values, a synchronous intensification of signals from Ni–H, Pd–H, and Pt/Rh–H species was observed.^46^ The synergistic adsorption and activation of H on multiple sites detected by SERS further confirmed that these specific multi-metallic hollow sites in NiSnPdPtRh@C-200 NRs act as the synergistic active centres for the HER. The SERS detection results were highly consistent with the theoretical calculations.

## Conclusions

In summary, uniform and well-defined NiSnPdPtRh HEA NRs were rapidly synthesized *via* a one-pot microwave-assisted strategy. A possible growth mechanism of the HEA NRs, based on time-resolved TEM characterization, is speculated as follows: directional assembly of pre-formed metal nanoclusters, followed by atomic co-deposition, fusion, diffusion, and final Ostwald ripening. The formation of the rod-like nanostructure was attributed to the structure-directing effect of Sn and Pd as well as the selective adsorption of Br^−^ ions, which collectively facilitated anisotropic growth of uniform NiSnPdPtRh NRs. Upon thermal annealing on the carbon support, the as-prepared NiSnPdPtRh@C-200 NRs exhibited excellent catalytic performance for acidic HER, requiring an ultra-low overpotential of 8.39 mV at 10 mA cm^−2^, accompanied by a very small Tafel slope (7.92 mV dec^−1^) and a remarkably high mass activity (2564 A g_noble_^−1^), significantly superior to the most reported HEA catalysts. Even at an industrial-level current density of 2000 mA cm^−2^, an overpotential of merely 196.5 mV was required. As a cathodic catalyst in PEMWE, NiSnPdPtRh@C-200 NRs delivered a lower cell voltage of 1.85 V and 1.98 V at 1.0 and 1.5 A cm^−2^, respectively, and operated steadily for up to 2000 h with an extremely low voltage decay of only 12.19 µV h^−1^ at 500 mA cm^−2^ under full water splitting conditions in acidic electrolyte. DFT calculations revealed that the Pt–Pt–Rh and Ni–Pt–Pd hollow sites represent most active sites for the HER, owing to their favourable hydrogen adsorption energetics and efficient charge transfer. SERS detection further confirmed these specific multi-metallic hollow sites in NiSnPdPtRh@C-200 NRs as the synergistic active centres for the HER. This work not only establishes a simple and rapid strategy for shape-controlled synthesis of HEA nanocrystals but also provides a deep insight for the design of high-efficiency multi-element electrocatalysts for industrial water electrolysis.

## Author contributions

Y. F. performed the main experiments and analyzed the data. S. L. conducted PEMWE. M. L. performed DFT calculations. K. X., Y. W., J. R. and W. K. N. performed some of the experiments. Z. Z. contributed to the discussion and provided suggestions. T. H. and X. K. supervised the work, directed the project and revised the manuscript. All authors discussed the results and commented on the manuscript.

## Conflicts of interest

There are no conflicts to declare.

## Supplementary Material

SC-OLF-D6SC06688G-s001

## Data Availability

All data needed to evaluate the conclusions are present in the article and/or the supplementary information (SI). Supplementary information: experimental procedures, computational details, and other characterization data. See DOI: https://doi.org/10.1039/d6sc06688g.
