# Supplementary material for: Microwave-irradiated ultrafast synthesis of NiSnPdPtRh high-entropy alloy nanorods for high-efficiency electrocatalytic hydrogen evolution reaction
Source: Chem Sci. 2026 Oct 22. Online ahead of print. doi: 10.1039/d6sc06688g (PMC13717512; doi:10.1039/d6sc06688g)
Supplement: Supplementary file 1 [file SC-OLF-D6SC06688G-s001.pdf]

Supplementary Information (SI) for Chemical Science.
This journal is © The Royal Society of Chemistry

Supplementary information (SI)

Microwave-Irradiated Ultrafast Synthesis of NiSnPdPtRh High-Entropy Alloy Nanorods for High-Efficiency Electrocatalytic Hydrogen Evolution Reaction

Yuan Fang,[†a] Siyuan Lai,[†b] Mairui Long,[†b] Keying Xing,[a] Yanfang Wu,[a] Junli Ren,[c] Zhan Zhang,[a] Wei Keat Ng,[b] Tao Huang[*a] and Xiongwu Kang[*b]

[a]Key Laboratory of Catalysis and Energy Materials Chemistry of Ministry of Education, College of Chemistry and Materials Science, South-Central Minzu University, Wuhan 430074, China

[b]School of Environment and Energy, South China University of Technology, Guangzhou 510006, China

[c]State Key Laboratory of Advanced Papermaking and Paper-based Materials, School of Light Industry and Engineering, South China University of Technology, Guangzhou 510640, China

*Corresponding author. E-mail: huangt208@mail.scuec.edu.cn (T. H.); esxkang@scut.edu.cn (X. K.)

[†]These authors contributed equally to this work.

Experimental section

Chemicals and materials: Hexachloroplatinic acid (H_2PtCl_6, 99%), palladium(II) chloride ($PdCl_2$, 99%) and rhodium(III) chloride ($RhCl_3$, 99%) was purchased from Yunnan Precious Metals Laboratory Co., Ltd. Nickel(II) chloride hexahydrate ($NiCl_2{\cdot}6H_2O$), triethylene glycol (TriEG) and commercial Pt Black was supplied from Aladdin Industrial Co. Ltd. Tin(II) chloride ($SnCl_2$) and 5 wt% Nafion were bought from Shanghai Macklin Biochemical Co., Ltd. Tetraethylene glycol (TEG) was purchased from J&K Scientific Co., Ltd. Polyvinylpyrrolidone (PVP, K30) was purchased from Beijing Vocation Biotech Co., Ltd. Potassium bromide (KBr) was purchased from Tianjin Kemiou Chemical Reagent Co., Ltd. Carbon black (C) was purchased from Shanghai Yien Chemical Technology Co., Ltd. Sodium bromide (NaBr), potassium chloride (KCl), potassium iodide (KI), hexadecyltrimethylammonium bromide (CTAB), tetrabutylammonium bromide (TBAB), ethylene glycol (EG) and glycerol (GLY) were bought from Sinopharm Chemical Reagent Shanghai Co. Ltd., China. All reagents were used directly without further purification.

Preparation of NiSnPdPtRh HEA NRs: Typically, 0.005 mmol of $NiCl_2$, 0.01 mmol of $SnCl_2$, 0.005 mmol of H_2PtCl_6, 0.005 mmol of $PdCl_2$, 0.005 mmol of $RhCl_3$, and 0.2 mmol of KBr were dissolved in 10 mL of TEG, stirred for 30 min, followed by the addition of 0.1 mmol PVP (K30) with continuous stirring for another 30 min. The molar ratio of $NiCl_2$/$SnCl_2$/$PdCl_2$/H_2PtCl_6/$RhCl_3$/PVP/KBr was 1/2/1/1/1/20/40. The solution was then put into a modified domestic microwave oven (Galanz, 900 W) and irradiated for 3 min with 100% output of the power under rigorous stirring. After cooling to room temperature, the precipitate was

collected by centrifugation at 10,000 rpm and washed 3–4 times with a mixed solvent of ethanol and water (5:3, v/v). Finally, the sample was dried under vacuum at 60 °C for 12 h, and NiSnPdPtRh NRs were obtained.

Under the same other conditions, quaternary alloys such as NiSnPdRh NRs or NiSnPdPt NRs were prepared in the absence of Pt or Rh precursor with the same molar ratio of other elements. Similarly, in addition, ternary SnPdRh NRs were also prepared in the absence of Ni and Pt precursors with the same molar ratio of other elements.

Loading of NiSnPdPtRh NRs on carbon powder followed by thermal treatment: 4 mg of NiSnPdPtRh NRs and 5 mg of commercial carbon (Vulcan XC-72, Carbon) were dispersed in 20 mL of ethanol. After ultrasonication for 30 min, carbon supported NiSnPdPtRh NRs (denoted as NiSnPdPtRh@C) was collected by centrifugation at 10000 rpm followed by drying under vacuum at 60 °C for 12 h. Then, NiSnPdPtRh@C was calcined at 180, 200, 300, or 400 °C for 2 h, and the corresponding samples were denoted as NiSnPdPtRh@C-180, NiSnPdPtRh@C-200, NiSnPdPtRh@C-300, and NiSnPdPtRh@C-400, respectively.

Under the same other conditions, similar carbon supported samples such as NiSnPdPt@C-200, NiSnPdRh@C-200 and SnPdRh@C-200 NRs were also prepared by loading of the corresponding quaternary and ternary alloys on carbon powder followed by thermal treatment at 200 °C, respectively.

Characterization: XRD was measured on an X-ray diffraction spectrometer (Bruker D8) with Cu K_α radiation under an operating condition at 40 kV and 50 mA. TEM, HRTEM and energy dispersive spectroscopy (EDS) were operated by a field emission transmission electron microscopy (FEI Talos F200X G2). XPS tests were conducted on an X-ray photoelectron spectrometer (ESCALAB 250Xi). The metal loadings and alloy compositions were quantified by using ICP-OES (Agilent 5110). Prior to ICP-OES analysis, the samples were pretreated with microwave digestion to ensure accurate quantification of all components. Synchrotron radiation-based EXAFS measurements for Pt L3-edge in transmission mode and Ni K-edge in fluorescence mode were performed with Shanghai Synchrotron Radiation Facility. Wavelet transform (WT) analyses were carried out by using Hama Fortran program.

Synchrotron radiation-based X-ray absorption spectroscopy: XAFS measurements at Pt L3-edge in transmission mode and Ni K-edge in fluorescence mode were performed at the Shanghai Synchrotron Radiation Facility (SSRF) (Shanghai, China). The obtained XAFS data were processed in Athena (version 0.9.26) for background, pre-edge line and post-edge line calibrations. Then Fourier transformed fitting was carried out in Artemis (version 0.9.26). The k^2 weighting, k-range of 3 - ~12 Å^{-1} and R range of 1 - 3 Å were used for the fitting of Pt foil and Ni foil; k-range of 3 - ~12 Å^{-1} and R range of ~1 – ~3 Å were used for the fitting of NiSnPdPtRh@C-200 NRs. The four parameters, coordination number, bond length, Debye-Waller factor, and E_0 shift (CN, R, σ^2, ΔE_0) were fixed. For wavelet transform analysis, the $\chi(k)$ exported from Athena was imported into the Hama Fortran code. The parameters were listed as follow: R range, 1 - 4 Å, k range, 0 - 12 Å^{-1}; k

weight, 2; and Morlet function with κ=10, σ=1 was used as the mother wavelet to provide the overall distribution.

Electrochemical measurements: Electrochemical measurements were performed on a CHI660E electrochemical workstation (Chenhua Instruments, Shanghai, China) using a standard three-electrode configuration. The performances of the as-prepared alloy catalysts in the hydrogen evolution reaction (HER) were evaluated by electrochemical measurements with the modified glassy carbon electrode (GCE) as the working electrode, a carbon rod as the counter electrode, and a saturated calomel electrode (SCE) as the reference (for acidic electrolytes). 5.0 mg of the catalyst was dispersed in a 2 mL solution containing 0.18 mL water, 1.8 mL ethanol, and 0.02 mL of 5 wt% Nafion, followed by ultrasonication for over 1 h to obtain a homogeneous catalyst ink (2.5 $mg{\cdot}mL^{-1}$). For each test, 8 μL of the catalyst ink was dropped onto the cleaned GCE surface (area: 0.1256 cm^2, corresponding to a catalyst loading of 0.1591 mg·cm⁻^{2}) and allowed to dry naturally for further use.

Linear sweep voltammetry (LSV) measurements were conducted in a 0.5 M H_2SO_4 solution (N_2-saturated, pH = 0.37) at a scan rate of 5 $mV{\cdot}s^{-1}$ in a potential range of 0.1 to –0.2 V vs. RHE. Electrochemical impedance spectroscopy (EIS) was measured over a frequency range of 10 kHz to 0.01 Hz, and the series resistance was determined from the x-intercept in the high-frequency region of the Nyquist plot, used for 90% iR correction.

The electrochemical active surface area (ECSA) was determined using the hydrogen adsorption method. The measurement was conducted using cyclic voltammetry (CV) in N₂-saturated 0.5 M

H_2SO_4 electrolyte, with a scan rate of 100 mV·s⁻¹ and a potential range of –0.3 to 0.3 V (vs. SCE). The ECSA calculation formula is as follows:

$$ECSA = \frac{S_H}{V_{scan} \times 0.21 \times 10^{-3} \times m_{metal}}$$

where ECSA ($m^2 \cdot g_{metal}^{-1}$) represents the electrochemical active surface area of the catalyst, S_H (A·V) is the integral area of hydrogen adsorption/desorption within the range of –0.3∼0.3V vs. SCE, V_{scan} (0.1 $V \cdot s^{-1}$) is the scan rate, 0.21 ($mC \cdot cm^{-2}$) is the theoretical charge density of unit area Pt, and m_{metal} (mg) is the total metal loading mass on the working electrode.

The turnover frequency (TOF) was calculated through the hydrogen adsorption charge (S_H). The TOF calculation formula is as follows:

$$N_{site} = \frac{S_H}{V_{scan} \times F}$$

$$TOF = \frac{Rate}{N_{site}} = \frac{i}{2 \times F \times N_{site}}$$

where TOF (s^{-1}) is the turnover frequency of the catalyst, N_{site} (mol) is the number of active sites on the catalyst surface, Rate ($mol \cdot s^{-1}$) is the reaction rate, i (A or $C \cdot s^{-1}$) is the reaction current after 90% IR correction, the number 2 represents the transfer of two electrons to produce each hydrogen molecule, and F (96485 $C \cdot mol^{-1}$) is the Faraday constant.

Faradaic efficiency (FE) was obtained by comparing the experimental hydrogen (H_2) yield with the theoretical hydrogen production, tested under constant current density of 50 $mA \cdot cm^{-2}$. The calculation formula is as follows:

$$EF = \frac{V_1(\text{measured})}{V_2(\text{theoretical})} = \frac{V_1 \times n \times F}{i \times t \times V_m}$$

where V_1 is the volume of H_2 collected experimentally, V_m (24 L·mol⁻¹) is the molar volume of gas at 293.15 K, n is the number of transferred electrons, F (96485 C·mol⁻¹) is the Faraday constant, and t (s) is the reaction time.

The mass activity (MA) and specific activity (SA) are typically calculated using the following formulas:

$$MA = \frac{i}{m_{noble}}$$

where MA is the mass activity of the catalyst ($A \cdot mg_{noble}^{-1}$), i(A) is the reaction current after 90% IR correction, and m_{noble} is the loading mass of noble metal on the working electrode.

$$SA = \frac{i}{ECSA \times m_{metal}}$$

where SA is the specific activity of the catalyst ($mA \cdot cm^{-2} \cdot mg^{-1}$), i(A) is the reaction current after 90% IR correction, ECSA is the electrochemical active surface area of the catalyst, and m_{metal} (mg) is the total metal loading mass on the working electrode.

In-situ Surface-Enhanced Raman Spectroscopy (SERS) test: SERS measurements were carried out using a HORIBA Scientific LabRAM HR Evolution spectrometer coupled with a three-electrode electrochemical cell, utilizing a 532 nm laser (10 mW) for excitation. The working electrode was prepared by depositing the NiSnPdPtRh@C-200 catalyst onto a carbon substrate (0.1 × 0.1 cm). A carbon rod and a SCE served as the counter electrode and the reference, respectively. Electrochemical control was maintained using a DH7001D workstation, with spectra collected after 300 s stabilization at each potential to ensure steady-state conditions. During in situ Raman

spectroscopy measurements, N_2-saturated 0.5 M H_2SO_4 was employed as the electrolyte to minimize bubble-induced signal interference in electrochemical HER.

Proton exchange membrane water electrolysis (PEMWE): The electrochemical measurement for PEMWE was evaluated using a CT2001A battery testing system (Wuhan Land Electronics Co., Ltd., China). Commercial IrO_2 was employed as the anodic catalyst with a relative Ir loading of 0.25 $mg_{Ir}·cm^{-2}$, while the as-synthesized NiSnPdPtRh@C-200 NRs served as the cathodic catalyst with a relative Pt loading of 0.3 $mg_{Pt}·cm^{-2}$. The catalyst samples were uniformly spray-coated onto both sides of a Nafion 117 proton exchange membrane, yielding an effective electrode area of 1.96 cm^2 (1.4 cm×1.4 cm). The electrolyzer was operated at a constant temperature of 65 °C, with ultrapure water being continuously supplied via a peristaltic pump at a flow rate of 25 $mL·min^{-1}$. Polarization curves were recorded from 1.0 to 2.2 V to assess the electrolyzer performance, while chronopotentiometric measurements were performed with a continuous operation for 2000 h at a current density of 500 $mA·cm^{-2}$ to evaluate the catalyst stability.

DFT calculations: Vienna Ab initio Simulation Package (VASP) was used for all first-principle calculations. The exchange-correlation interactions were described using Perdew-Burke-Ernzerhof (PBE) functional, and a cutoff energy of 400 eV was set for the plane-wave basis. The convergence criterions for electronic self-consistent field calculation and geometry optimization was set at 1×10^{-4} eV and 0.02 $eV·Å^{-1}$, respectively. The surface model was constructed along the <111> direction of a face-centered cubic (FCC) structure, consisting of 3 atomic layers with 48 atoms in total. For

geometric optimization, a 15 Å vacuum layer was set perpendicular to the surface to eliminate periodic boundary effects. Brillouin zone integration was performed using a 3×3×1 Monkhorst-Pack k-point grid sampling, and dispersion interactions were described using Grimme's DFT-D3 method. The Gibbs free energy diagram of H* adsorption of NiSnPdPtRh HEA NRs for HER was constructed using the computational hydrogen electrode (CHE) model, and the free energy (G) of adsorbed species was calculated using the following equation:

$$G = E + ZPE - T \, ∙ \, S$$

where E is the total energy obtained from DFT calculations, ZPE is the zero-point energy, and T∙S is the entropy contribution term.

Supplementary Figures

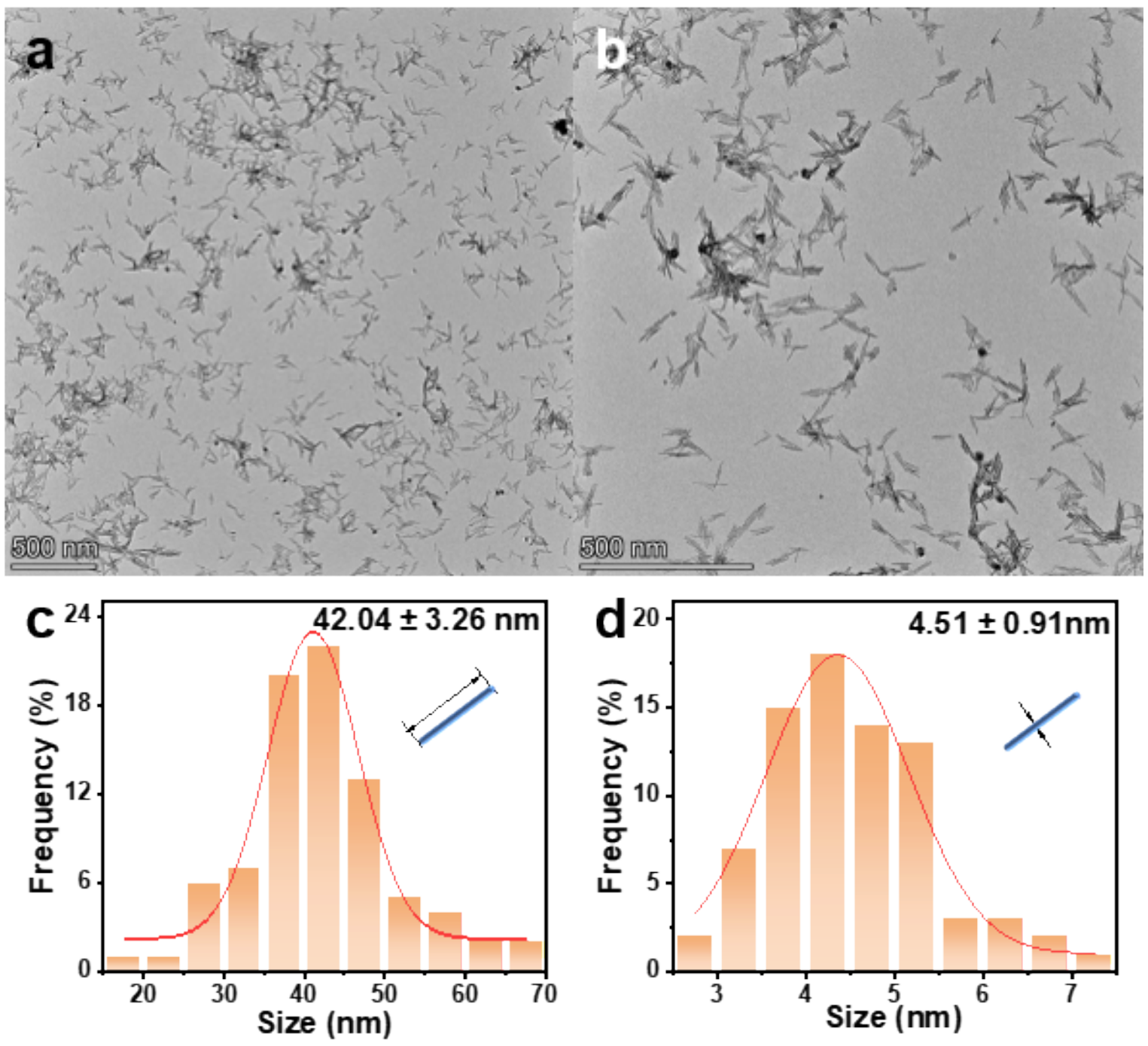

Fig. S1. The dimensional statistics of the NiSnPdPtRh NRs sample obtained under microwave irradiation for 180 s at 900 W. (a) and (b) TEM images of NiSnPdPtRh NRs. The statistical histograms of the average length (c) and diameter (d).

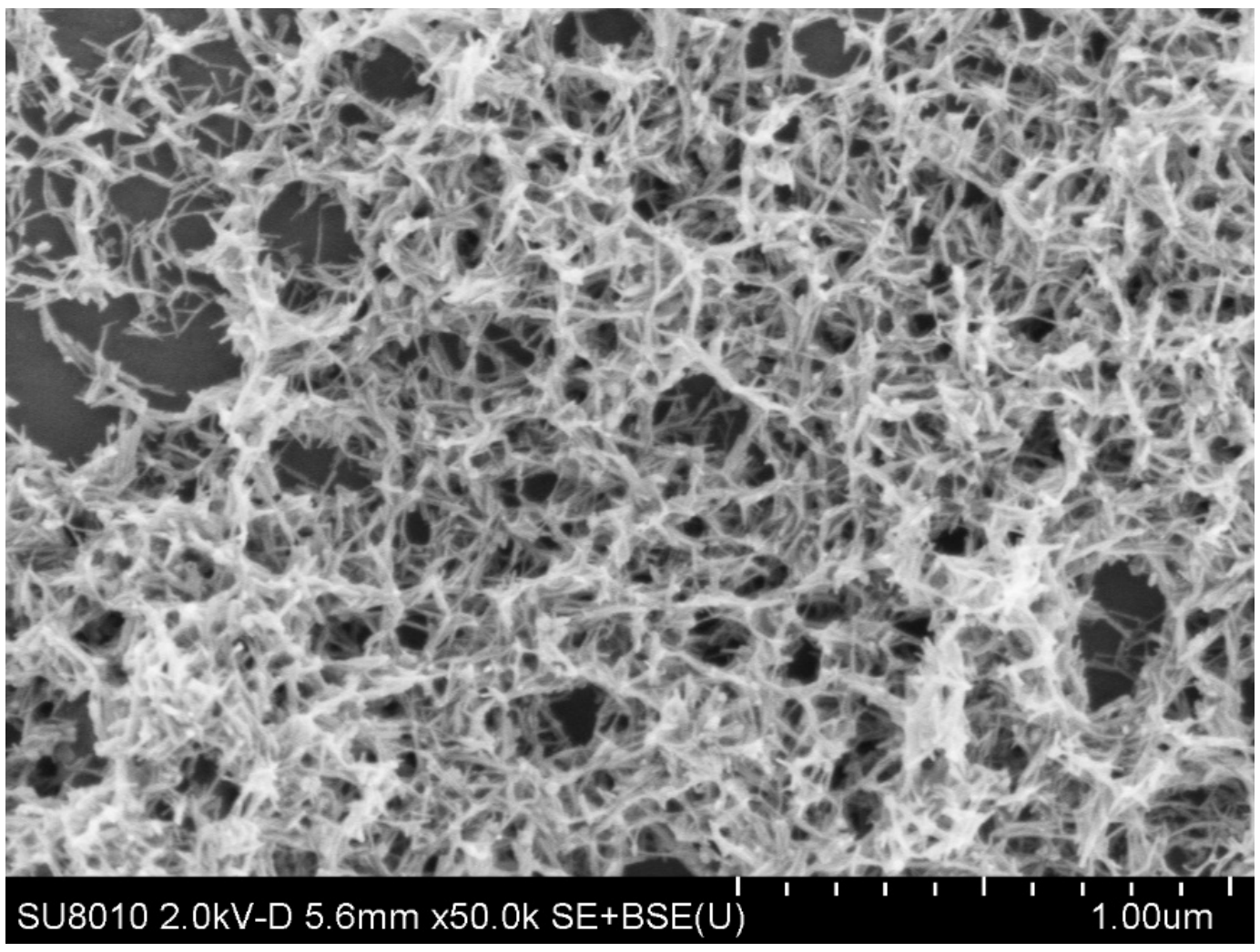

Fig. S2. SEM image of NiSnPdPtRh HEA NRs. The sample obtained under 900 W microwave irradiation.

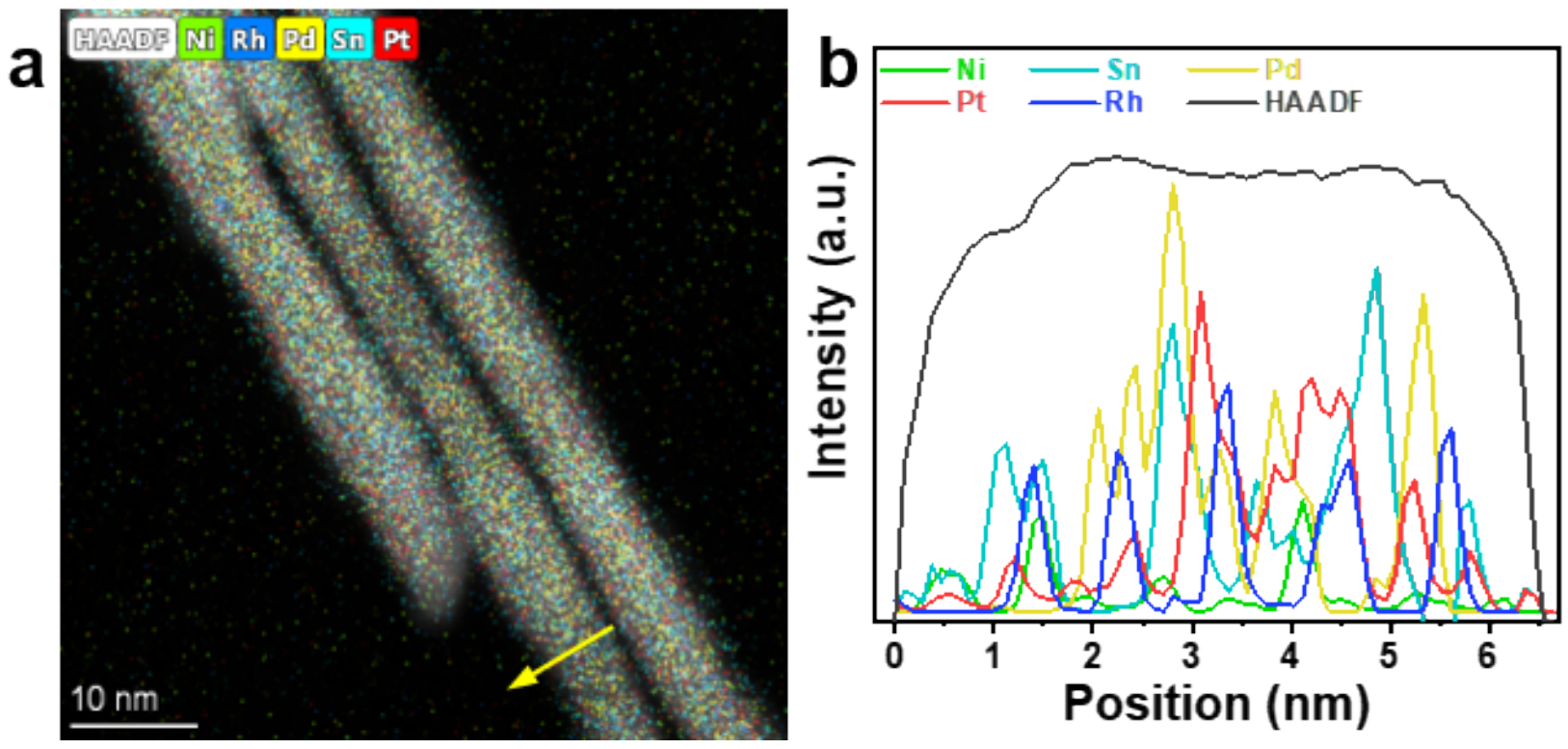

Fig. S3. The line-scan EDS analysis of the as-prepared NiSnPdPtRh HEA NRs obtained under 900 W microwave irradiation. (a) HAADF-STEM image. (b) Line scanning profile along the yellow arrow.

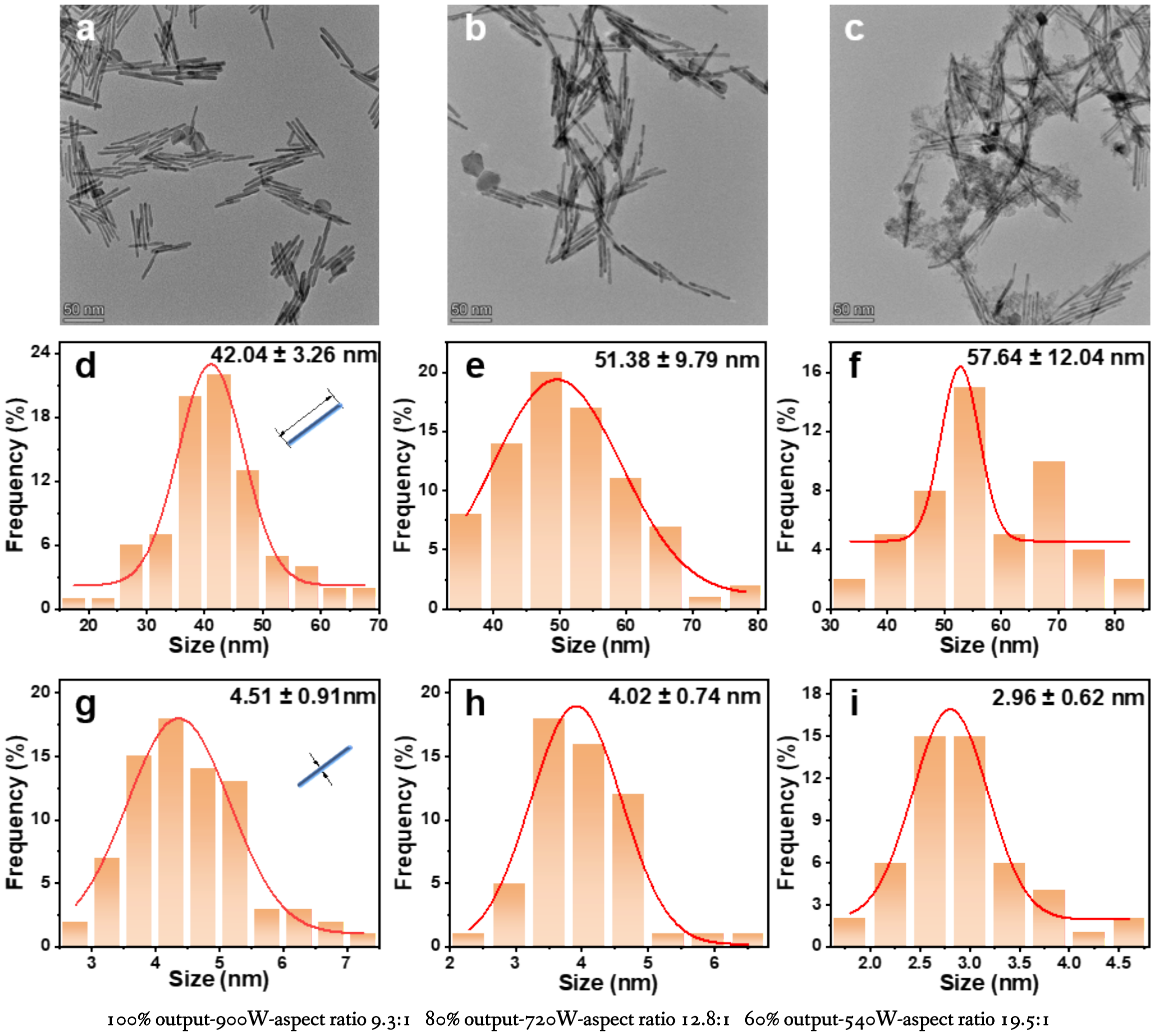

Fig. S4. TEM images and statistical histograms of length and diameter of NiSnPdPtRh NRs synthesized under irradiation for 180 s with microwave power of 900 W(a, d, g), 720 W(b, e, h), 540 W(c, f, i).

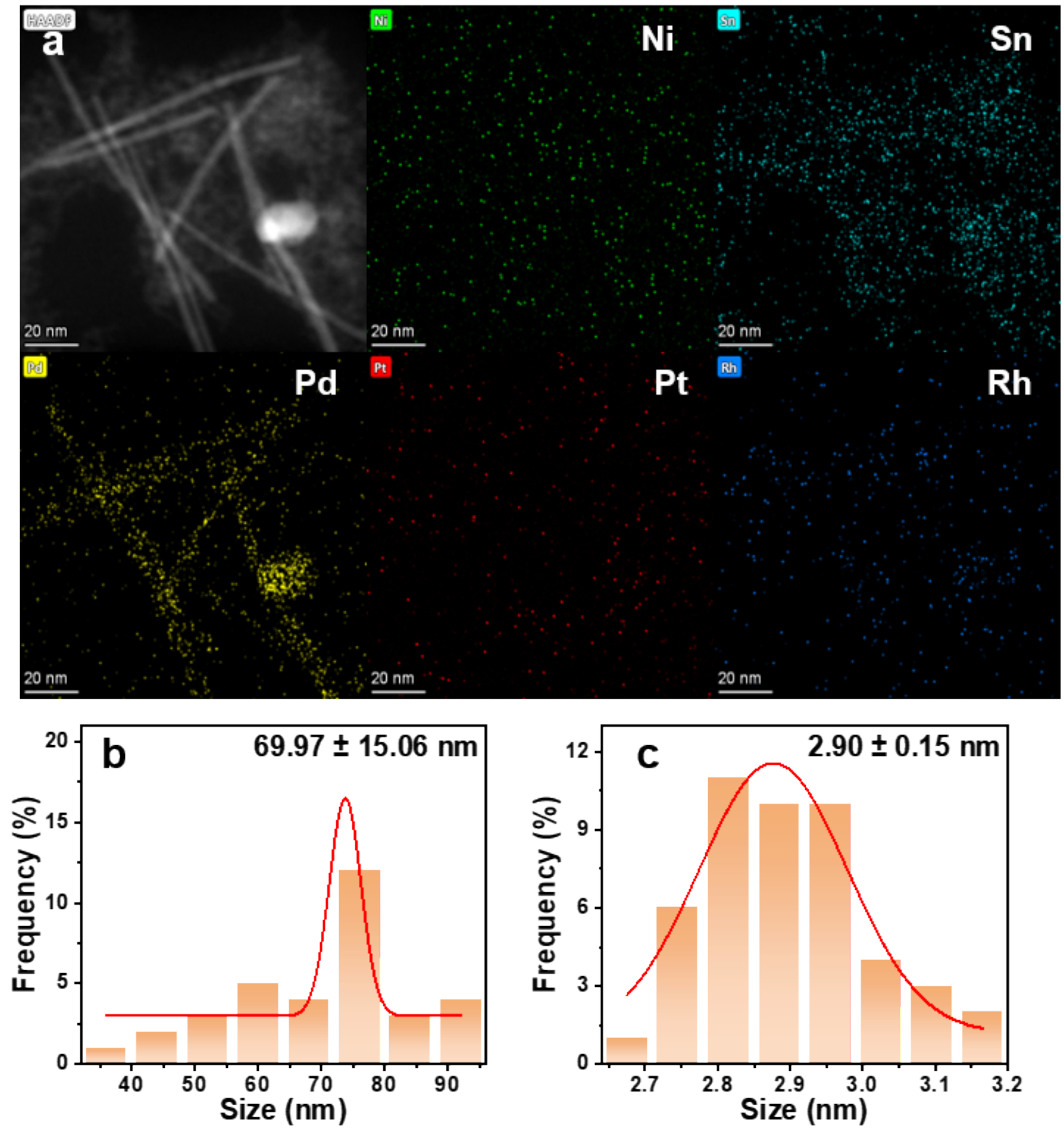

Fig. S5. EDS analyses and the size of NiSnPdPtRh NRs prepared under 540 W irradiation for 120 s. (a) HAADF-STEM image and the corresponding EDS mapping images, and the histograms of (b) length and (c) diameter.

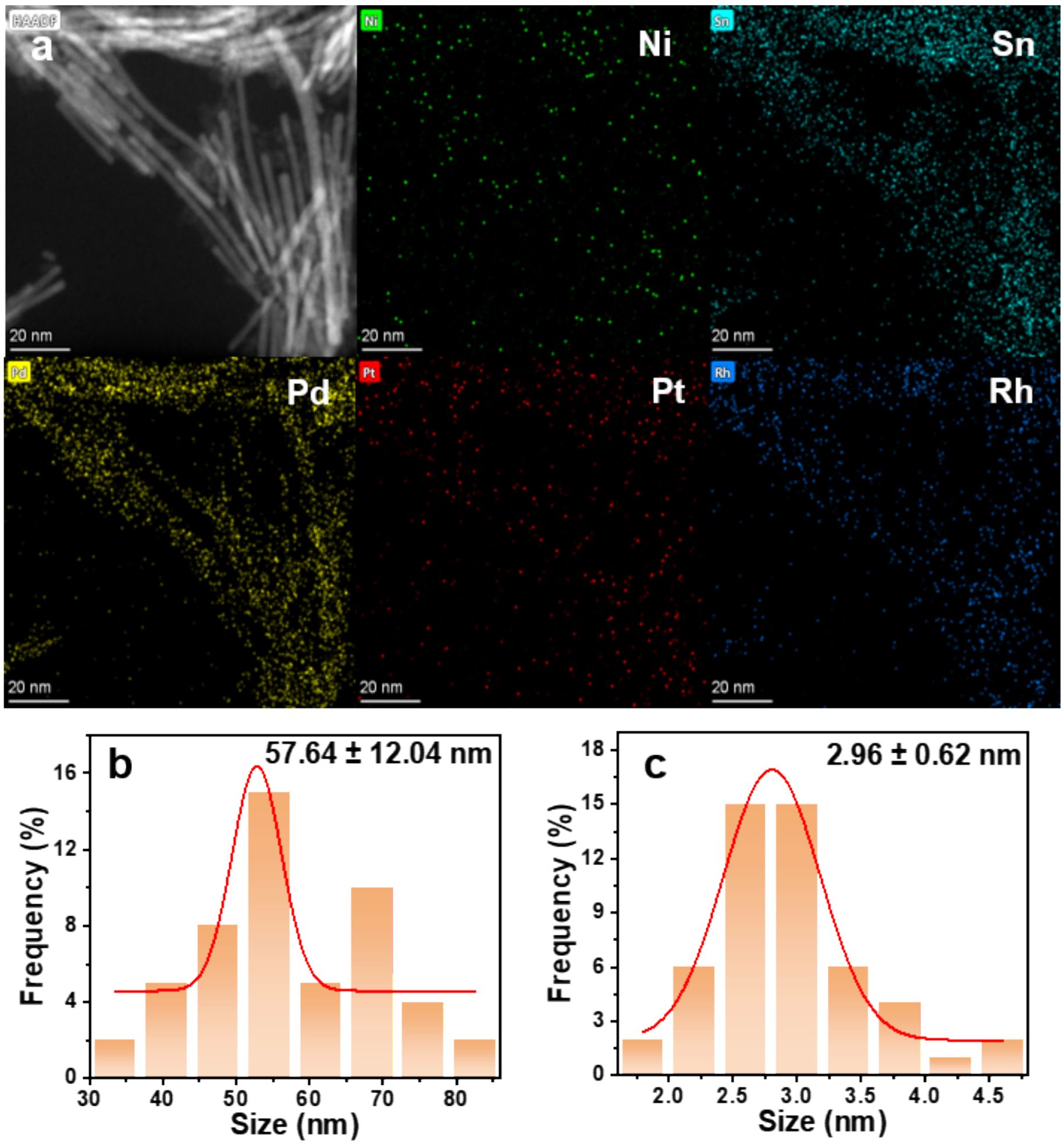

Fig. S6. EDS analyses and the size of NiSnPdPtRh NRs prepared under 540 W irradiation for 180 s. (a) HAADF-STEM image and the corresponding EDS mapping images, and the histograms of (b) length and (c) diameter.

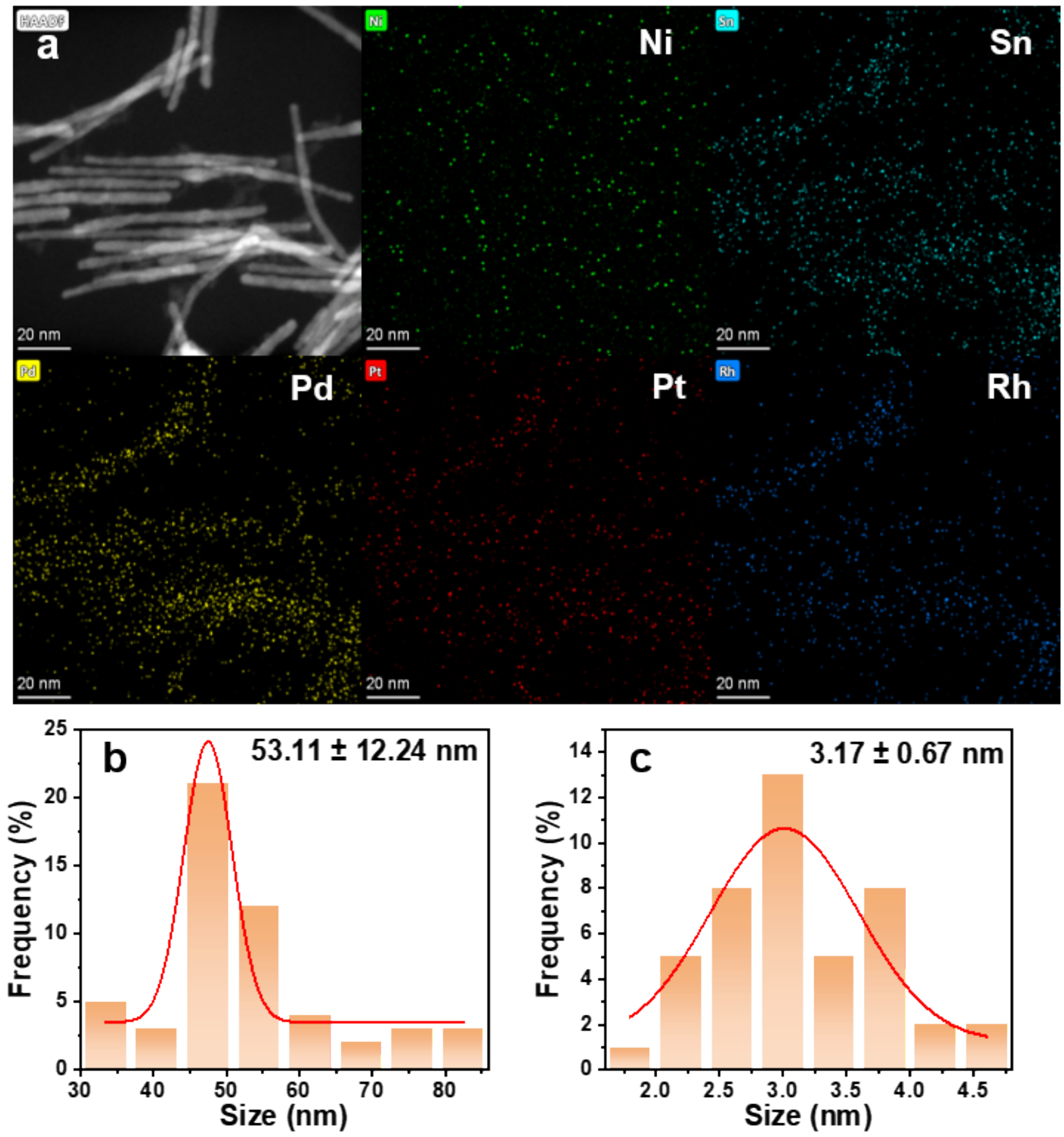

Fig. S7. EDS analyses and the size of NiSnPdPtRh NRs prepared under 540 W irradiation for 240 s. (a) HAADF-STEM image and the corresponding EDS mapping images, and the histograms of (b) length and (c) diameter.

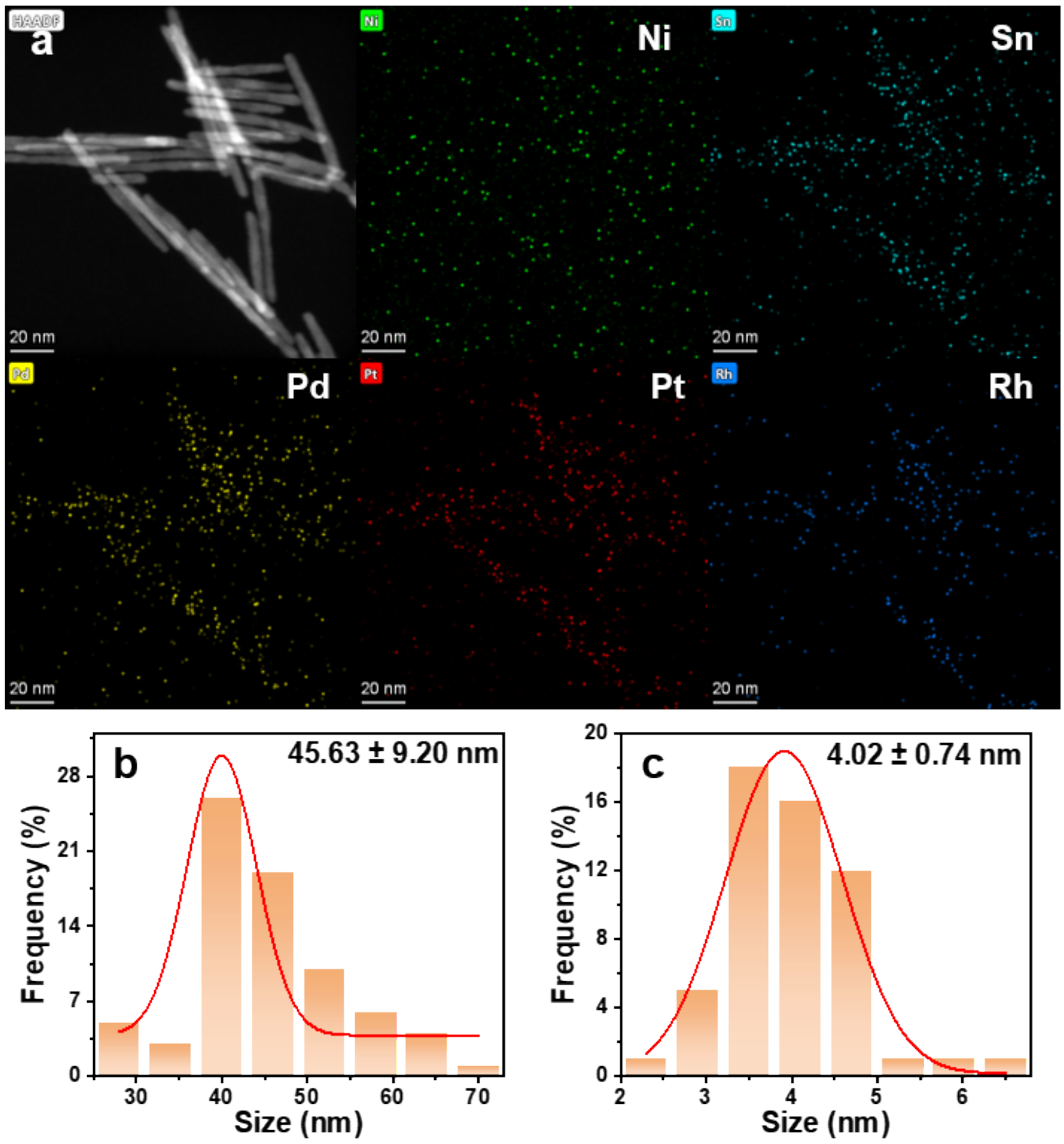

Fig. S8. EDS analyses and the size of NiSnPdPtRh NRs prepared under 540 W irradiation for 300 s. (a) HAADF-STEM image and the corresponding EDS mapping images, and the histograms of (b) length and (c) diameter.

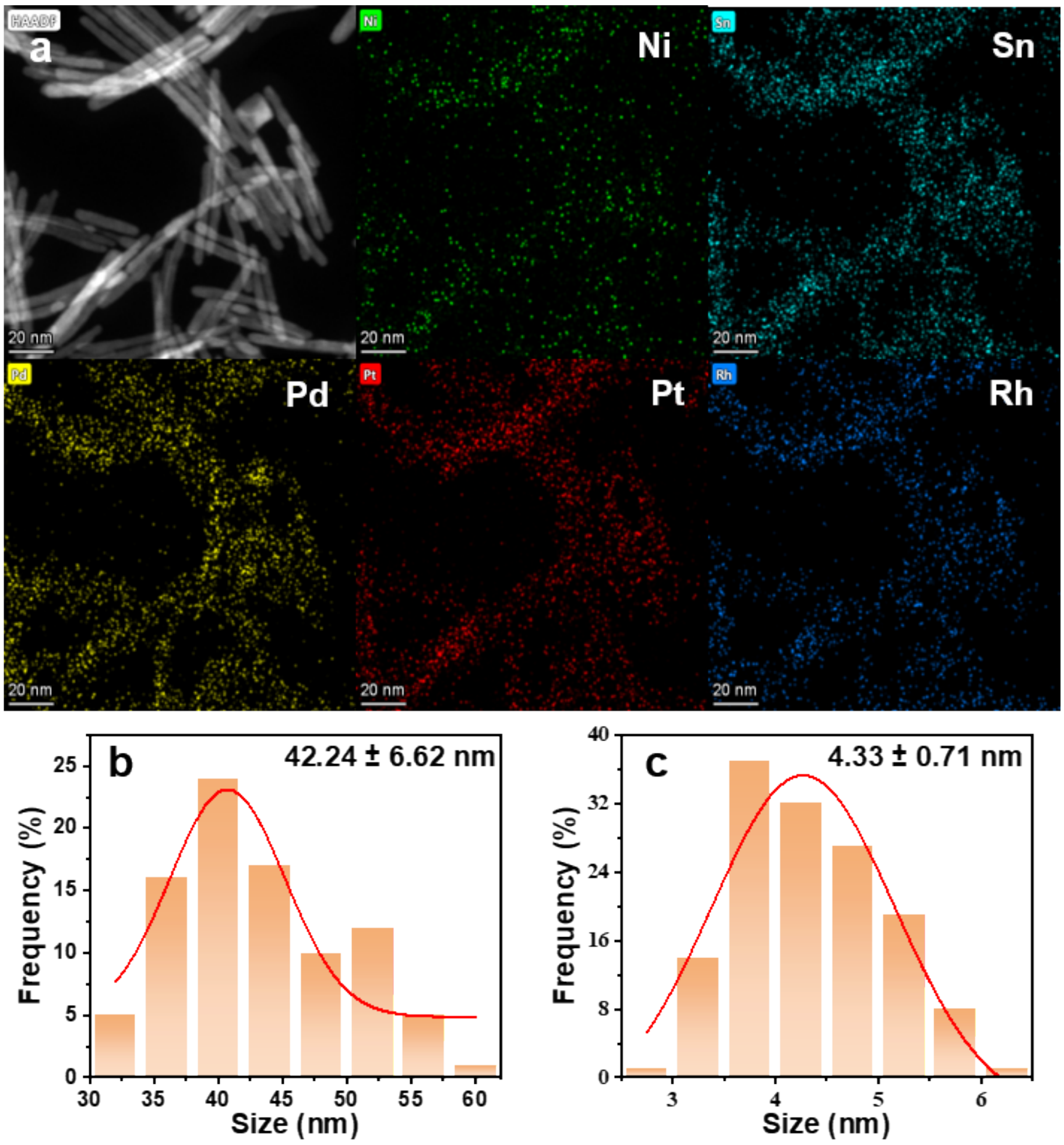

Fig. S9. EDS analyses and the size of NiSnPdPtRh NRs prepared under 540 W irradiation for 360 s. (a) HAADF-STEM image and the corresponding EDS mapping images, and the histograms of (b) length and (c) diameter.

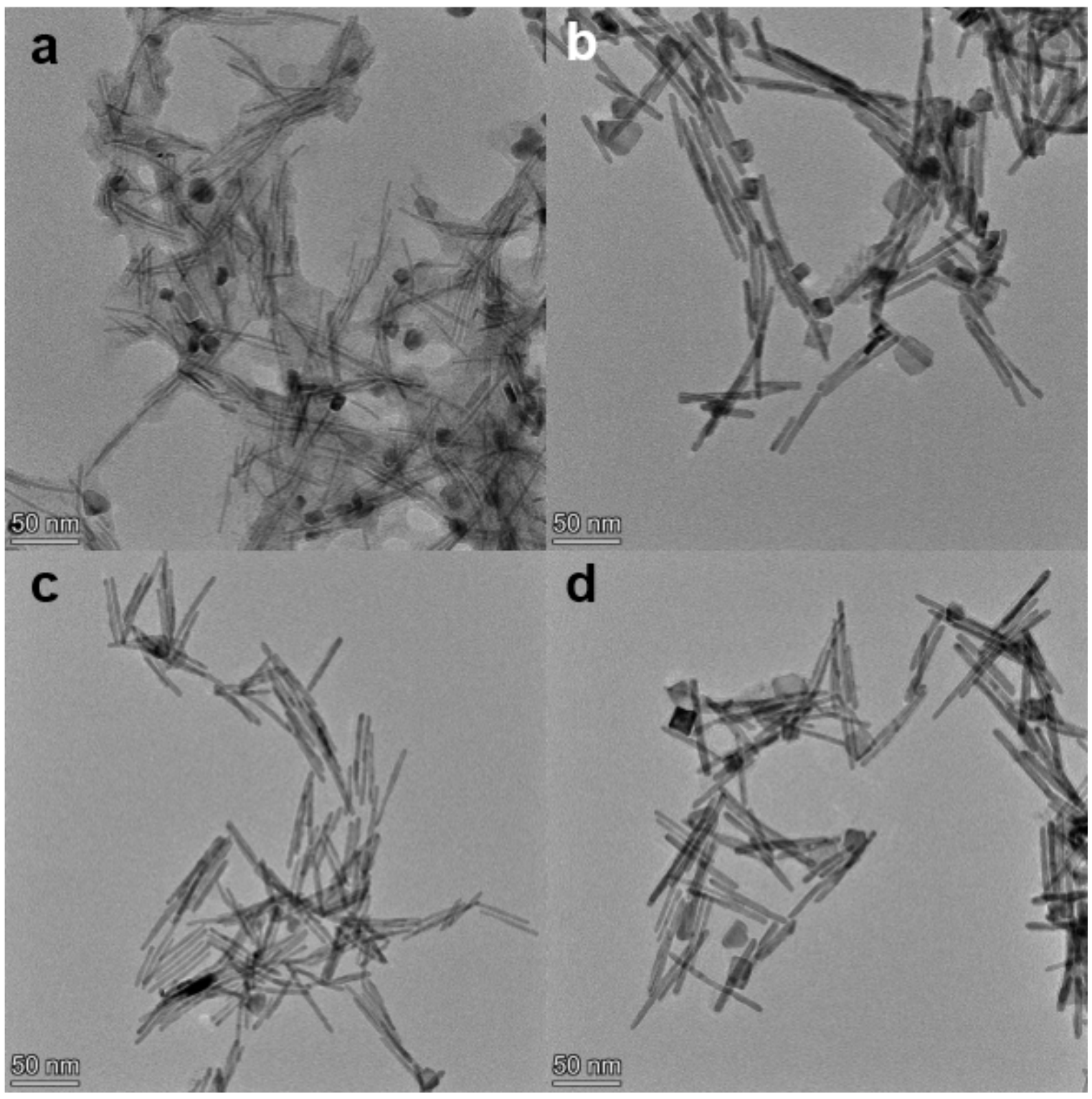

Fig. S10. TEM images of NiSnPdPtRh NRs synthesized with different microwave reaction times under 900 W irradiation. (a) 90 s. (b) 120 s. (c) 150 s. (d) 210 s.

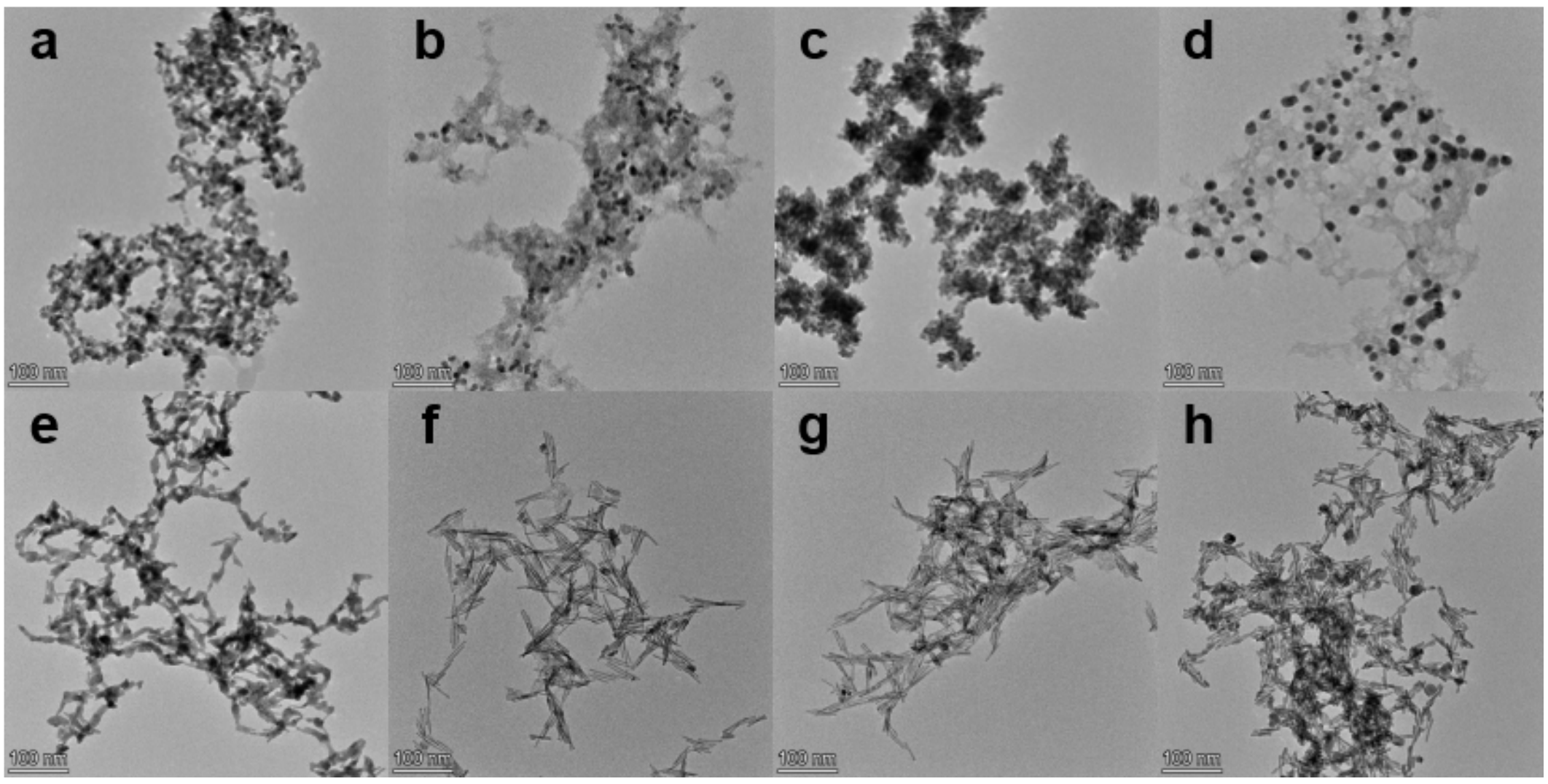

Fig. S11. TEM images of products with different compositions. (a) Pd. (b) SnPd. (c) NiPtRh. (d) NiSnPd. (e) SnPdPt. (f) SnPdRh. (g) NiSnPdRh. (h) NiSnPdPt.

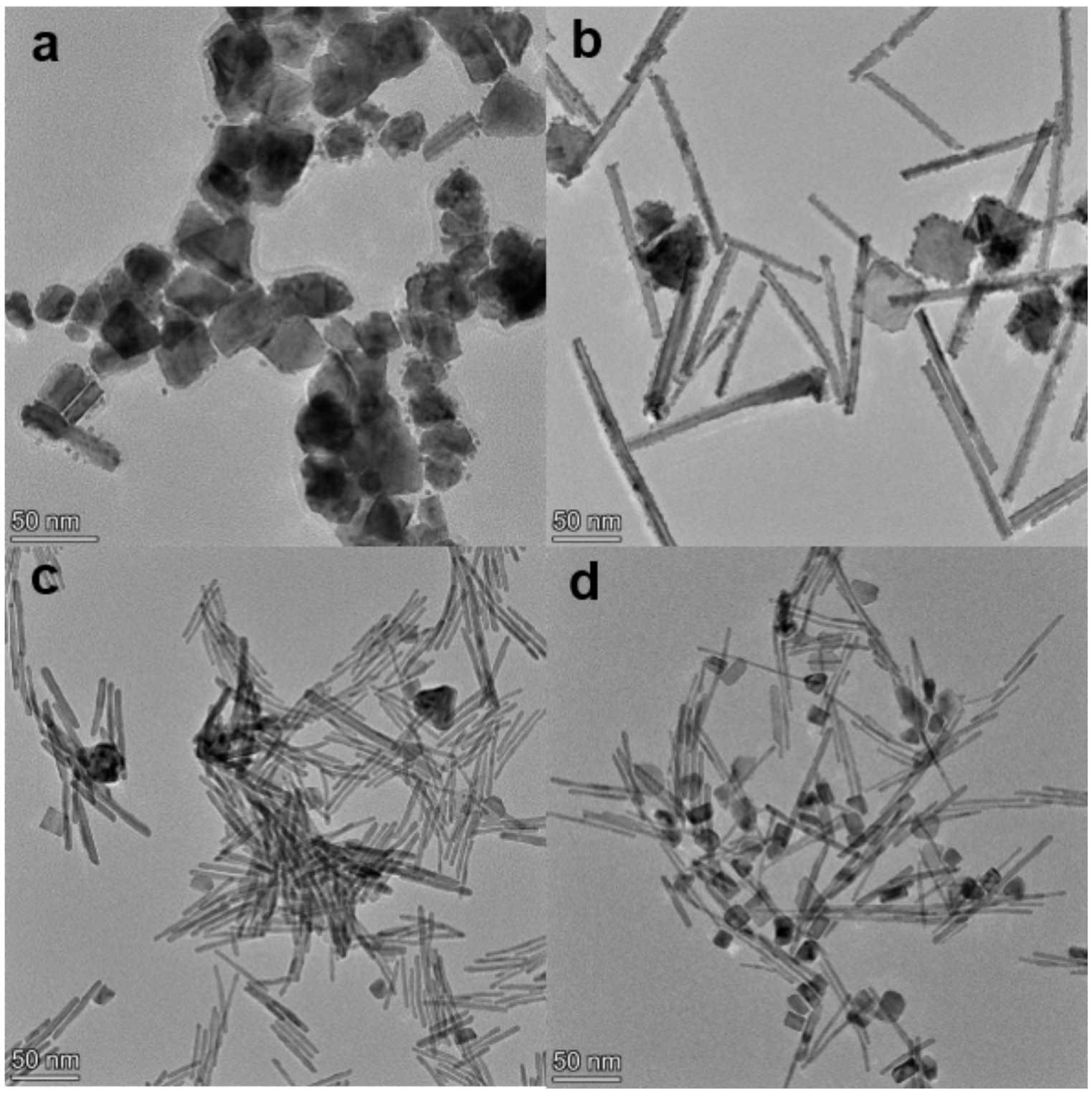

Fig. S12. The effect of different amounts of KBr on the formation of NiSnPdPtRh NRs under 900 W irradiation for 180 s. (a) 0 mmol. (b) 0.1 mmol. (c) 0.2 mmol. (d) 0.3 mmol.

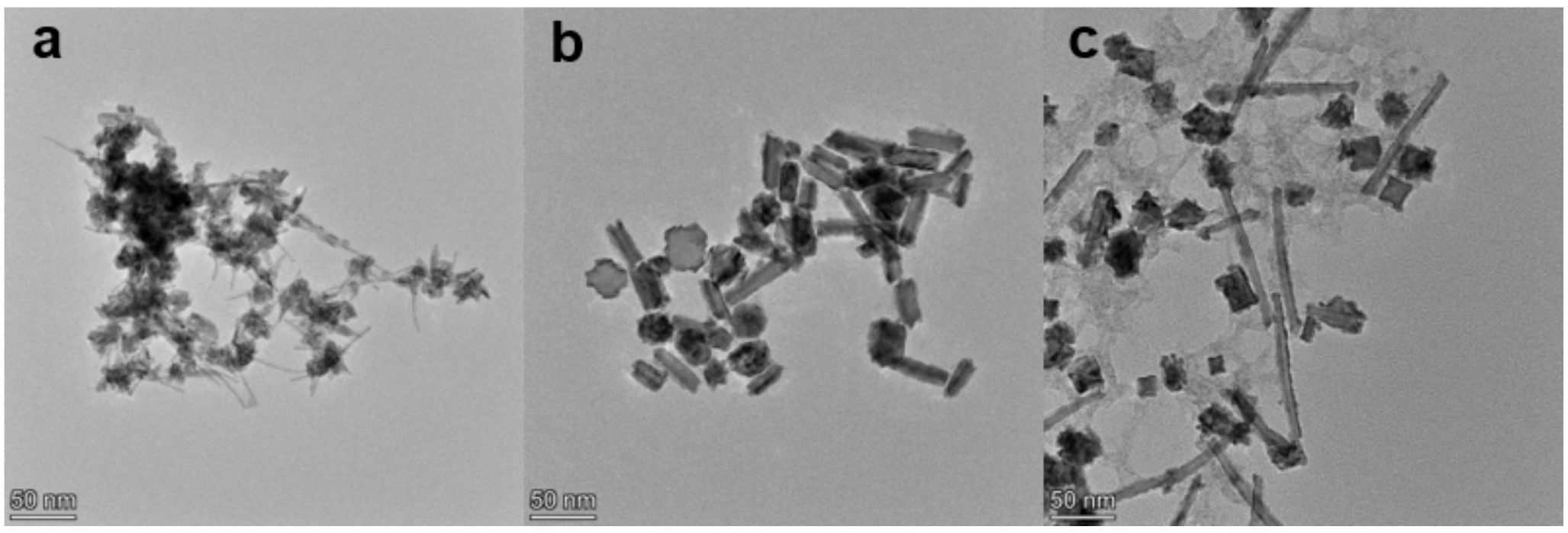

Fig. S13. TEM images of NiSnPdPtRh NRs prepared in the presence of 0.2 mmol halides. (a) KI; (b) KCl; (c) NaBr.

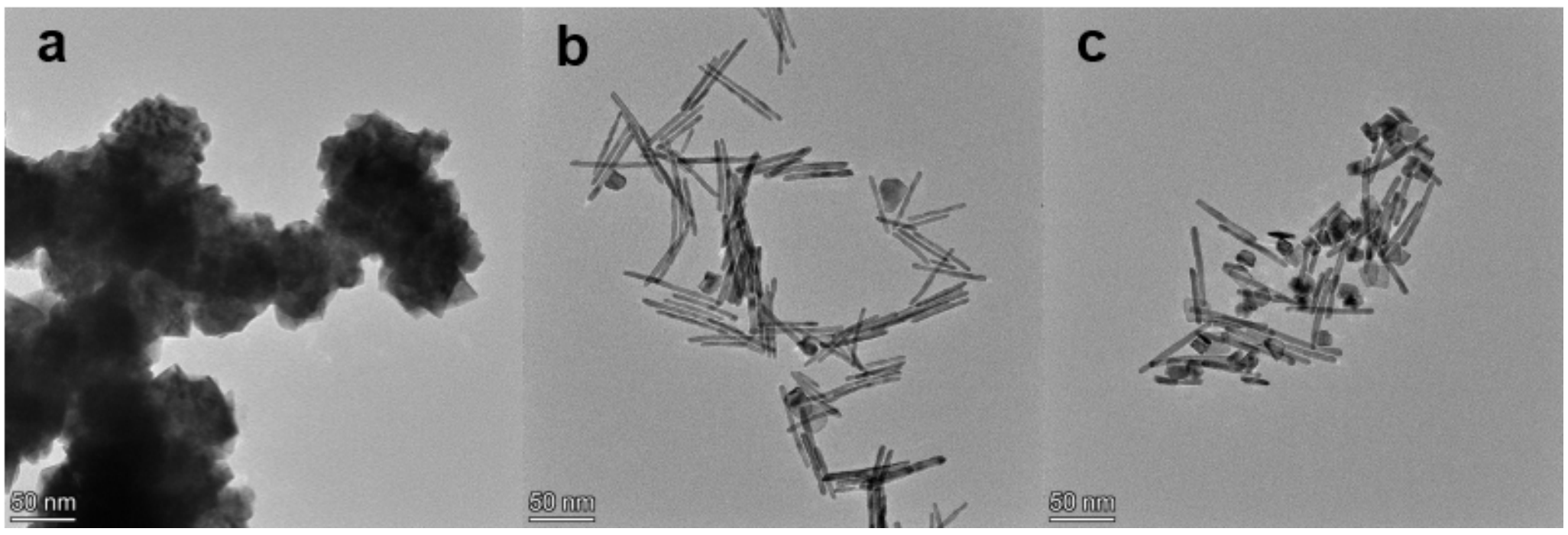

Fig. S14. The TEM image of NiSnPdPtRh NRs prepared in the presence of different amount of PVP. (a) 0 mmol; (b) 0.05 mmol; (c) 0.15 mmol.

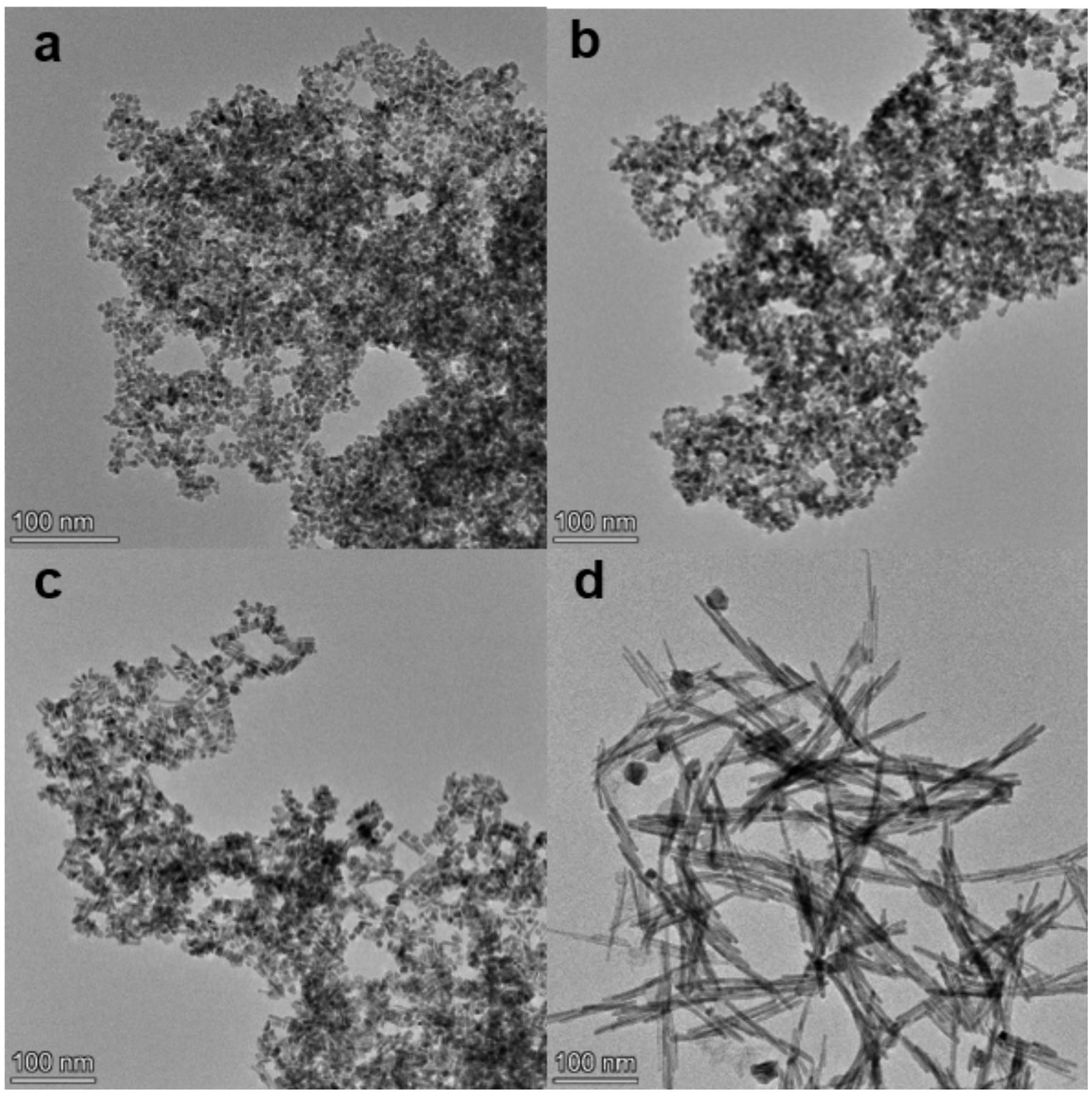

Fig. S15. TEM images of NiSnPdPtRh HEA nanomaterials prepared with different solvents. (a) EG. (b) GLY. (c) TriEG. (d) TEG.

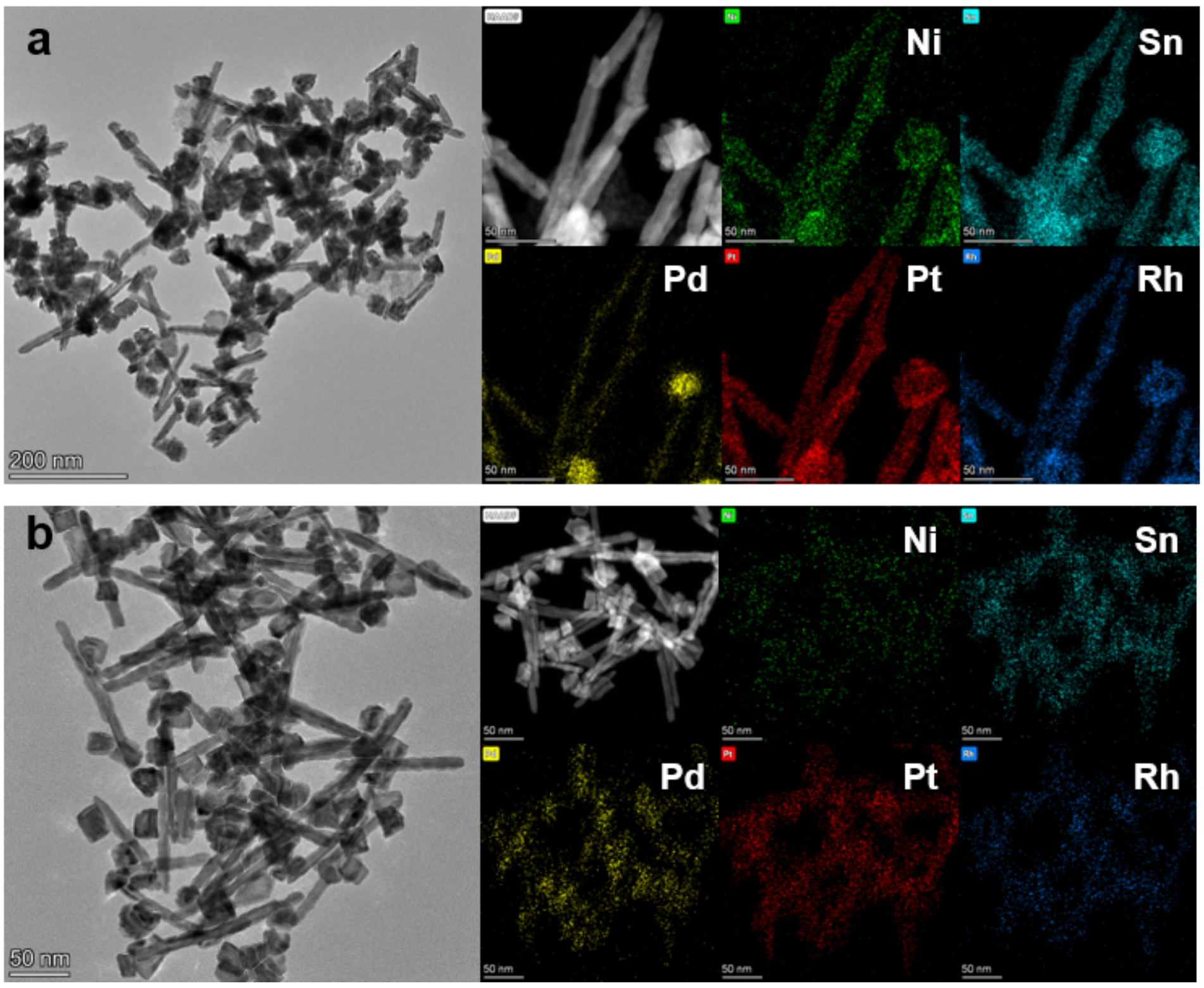

Fig. S16. The effect of H_2PtCl_6 dosage on the formation of NiSnPdPtRh NRs. (a) 0.01 mmol. (b) 0.015 mmol.

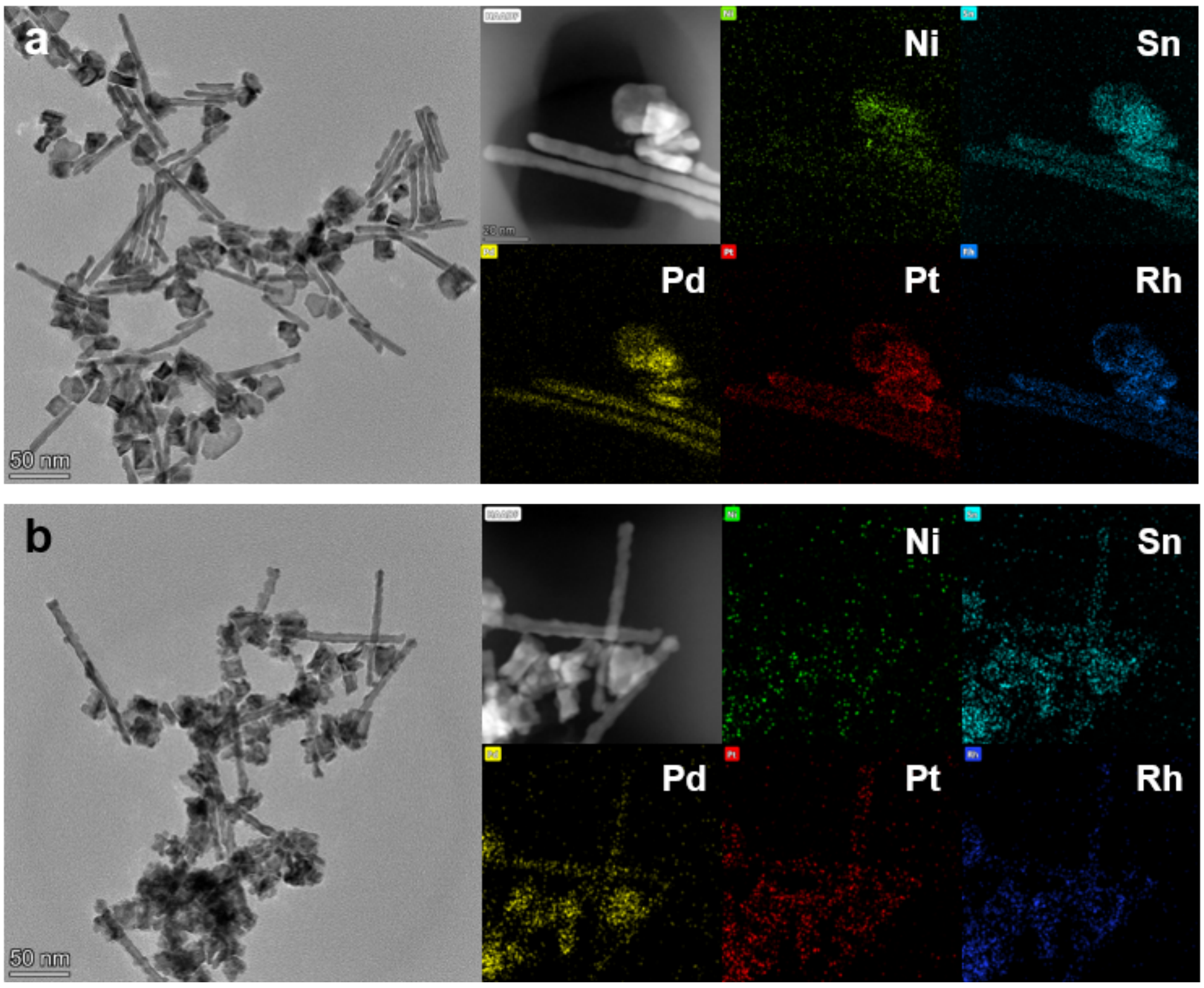

Fig. S17. The effect of $RhCl_3$ dosage on the formation of NiSnPdPtRh NRs. (a) 0.01 mmol. (b) 0.015 mmol.

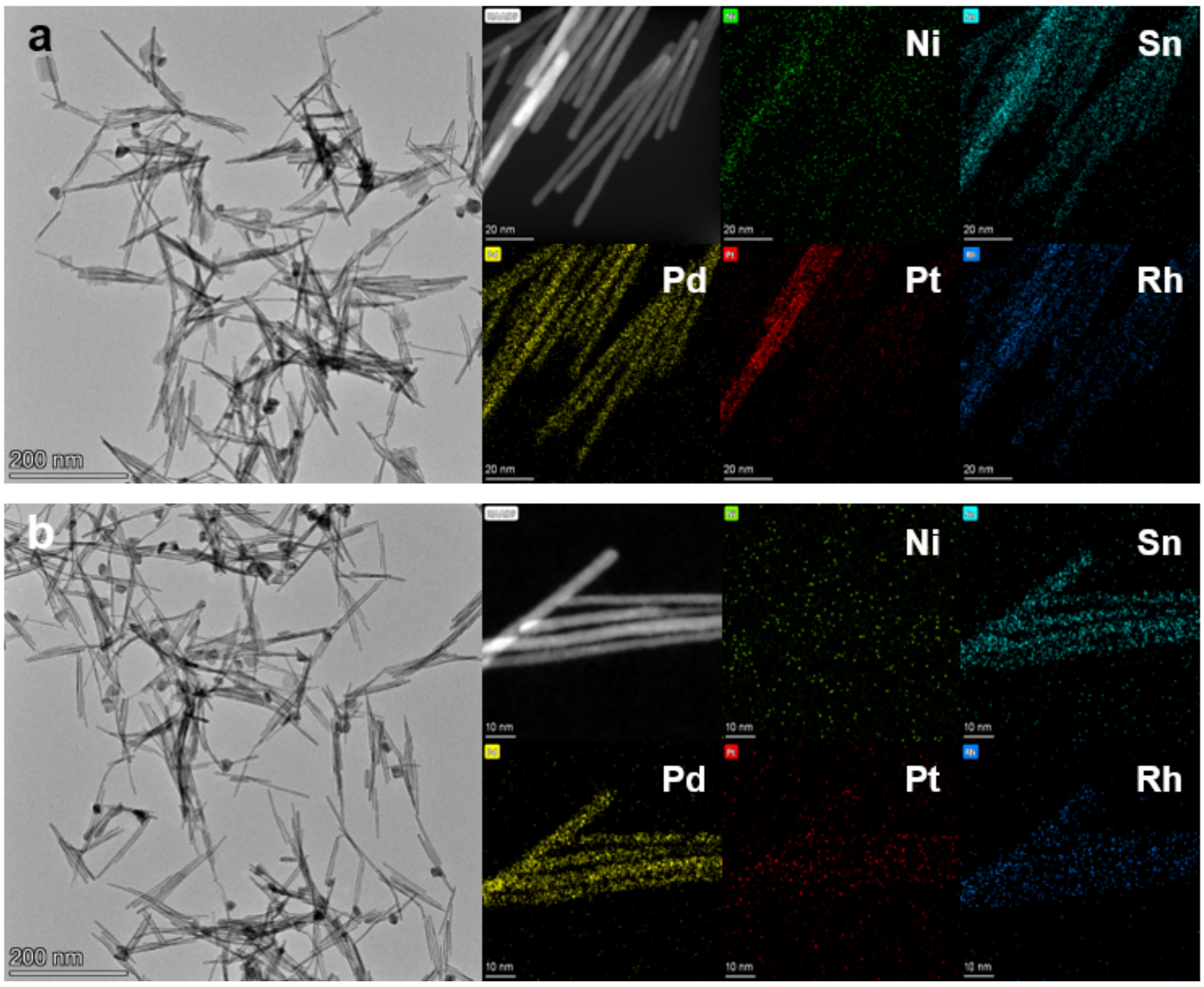

Fig. S18. The effect of $PdCl_2$ dosage on the formation of NiSnPdPtRh NRs. (a) 0.01 mmol. (b) 0.015 mmol.

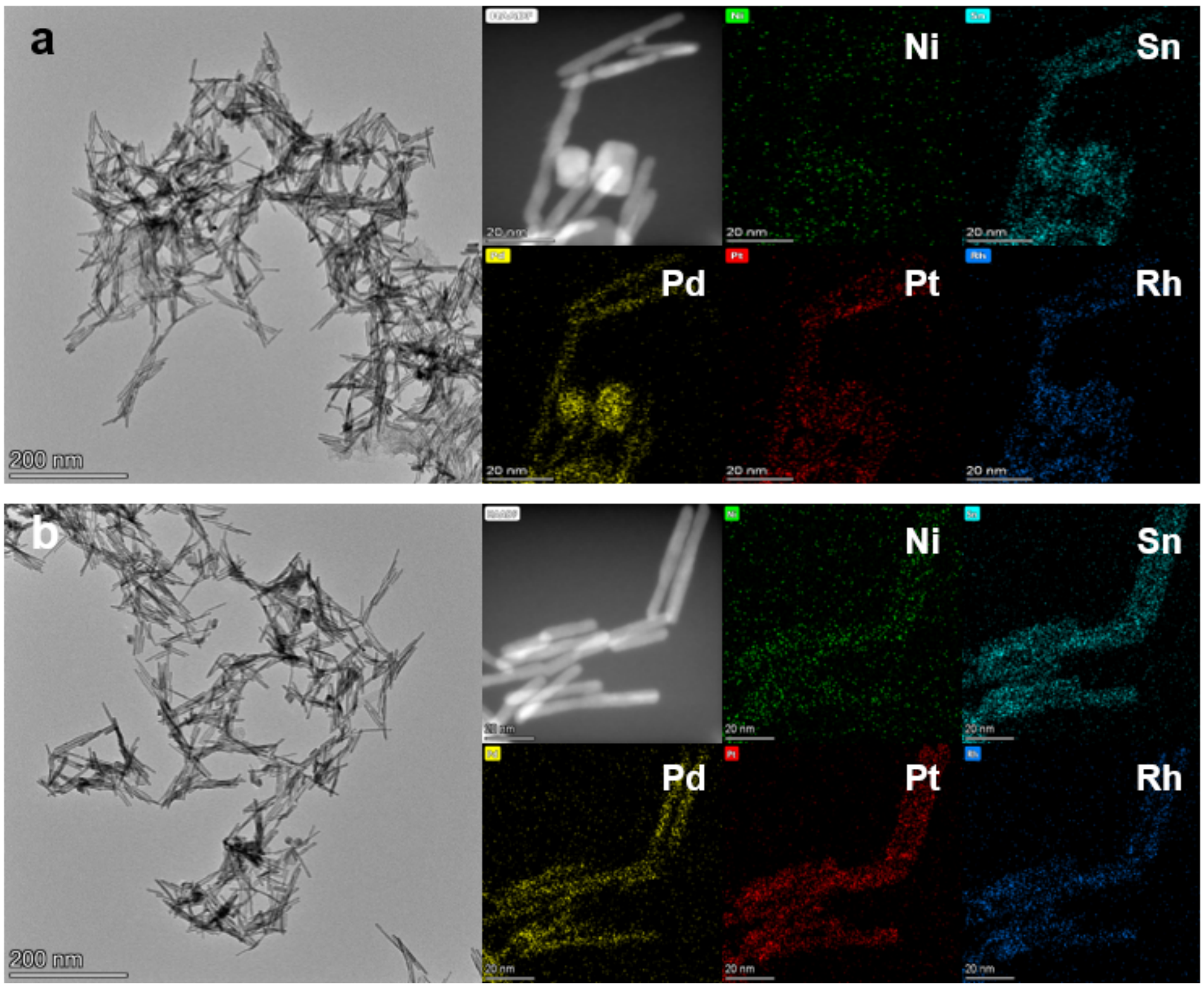

Fig. S19. The effect of $NiCl_2$ dosage on the formation of NiSnPdPtRh NRs. (a) 0.01 mmol. (b) 0.015 mmol.

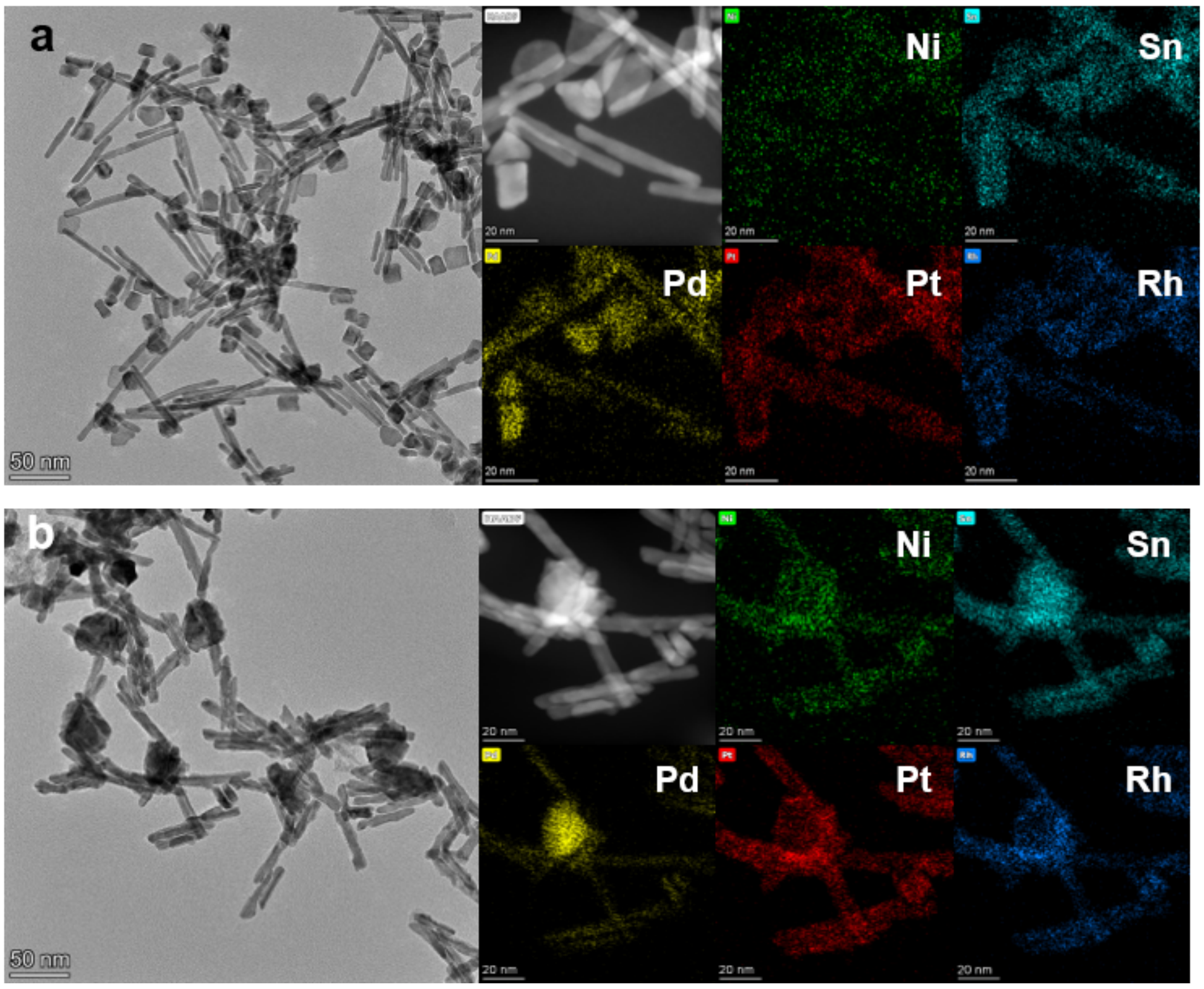

Fig. S20. The effect of $SnCl_2$ dosage on the formation of NiSnPdPtRh NRs. (a) 0.005 mmol; (b) 0.015 mmol.

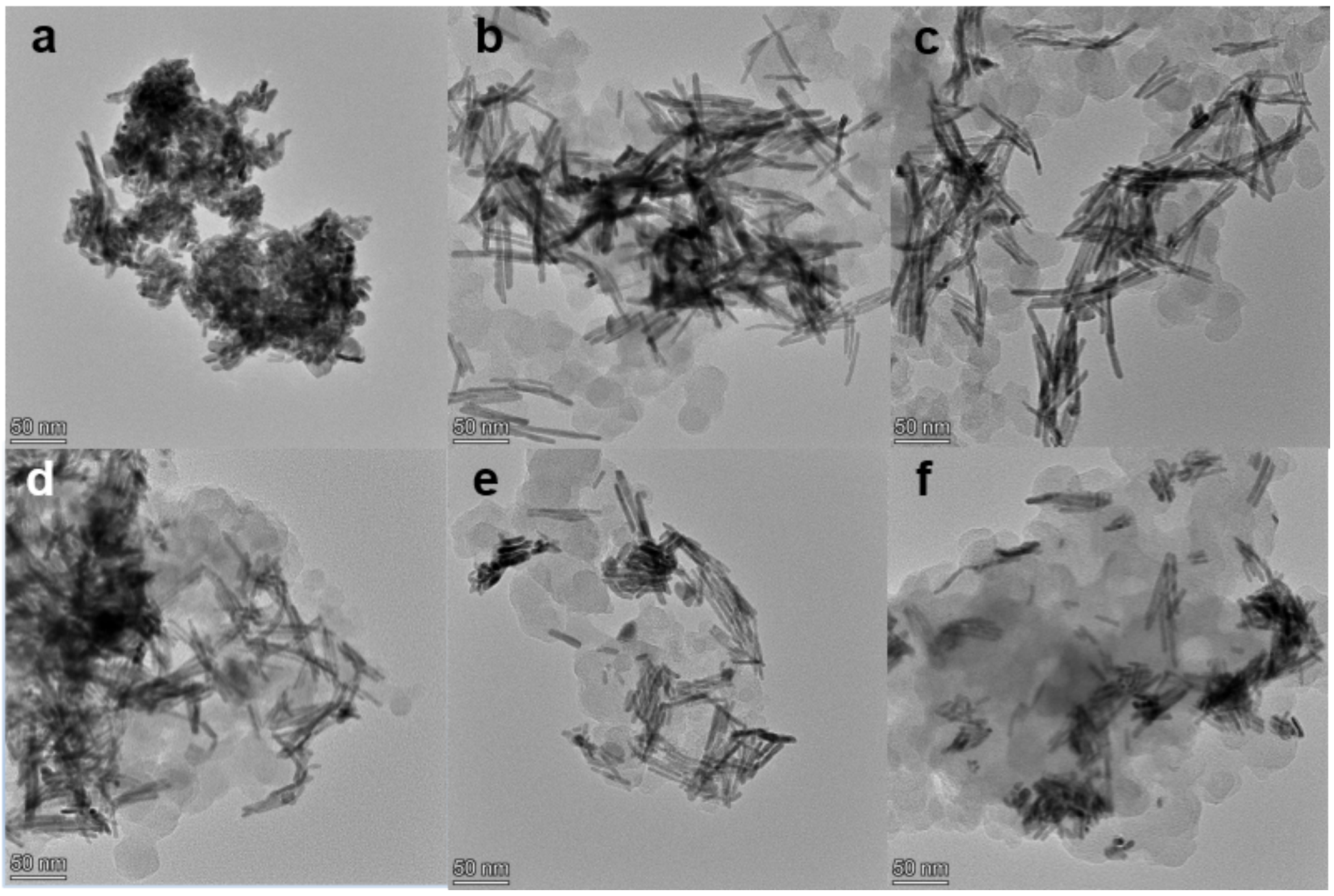

Fig. S21. TEM images of different samples under different calcinated temperatures. (a) NiSnPdPtRh-200; (b) NiSnPdPtRh@C; (c) NiSnPdPtRh@C-180; (d) NiSnPdPtRh@C-200; (e) NiSnPdPtRh@C-300; (f) NiSnPdPtRh@C-400.

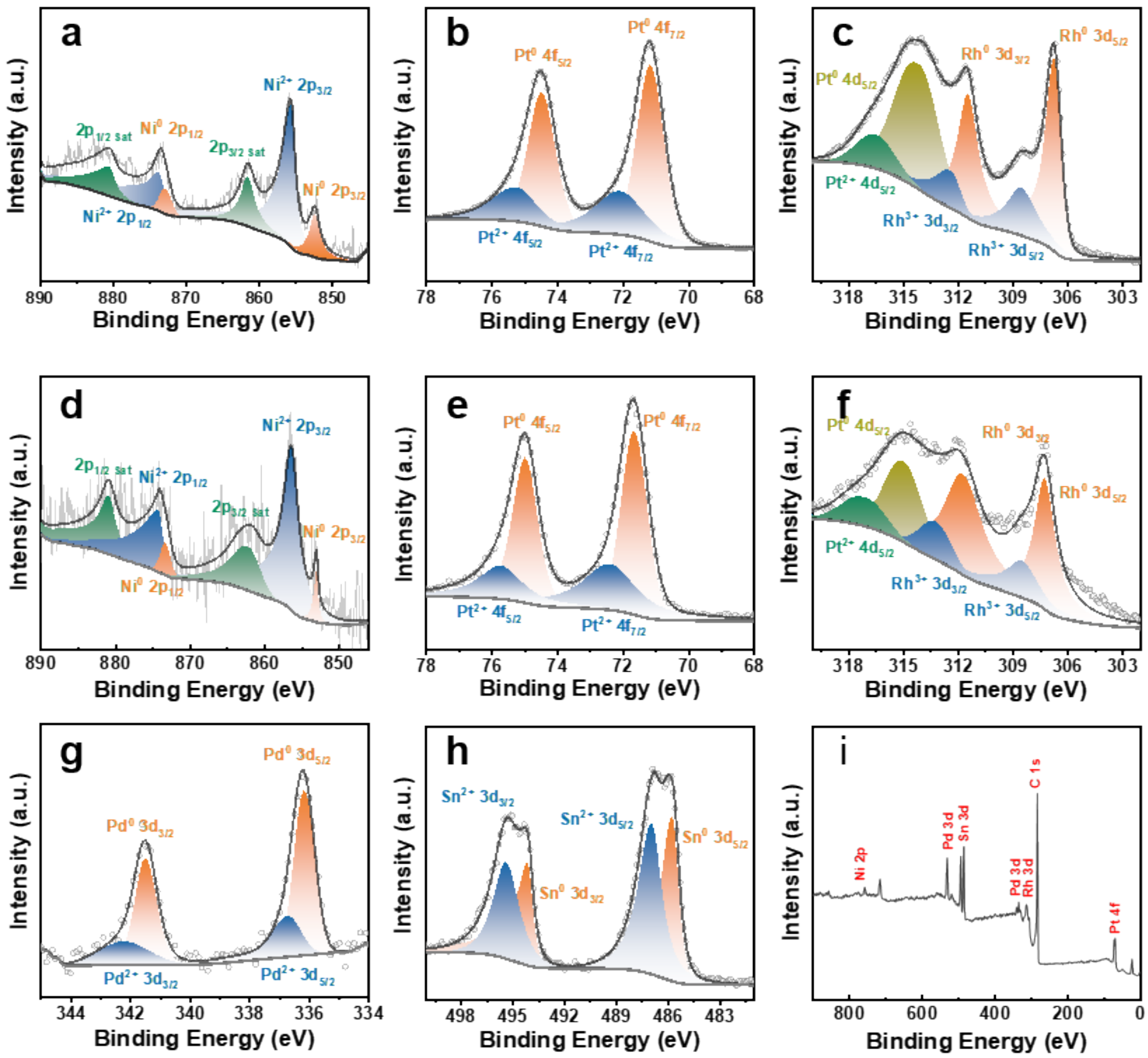

Fig. S22. XPS spectra. (a) Ni 2p, (b) Pt 4f and (c) Rh 3d regions of NiSnPdPtRh NRs; (d) Ni 2p, (e) Pt 4f, (f) Rh 3d, (g) Pd 3d, (h) Sn 3d regions and (i) the full XPS spectrum of NiSnPdPtRh@C-200 NRs.

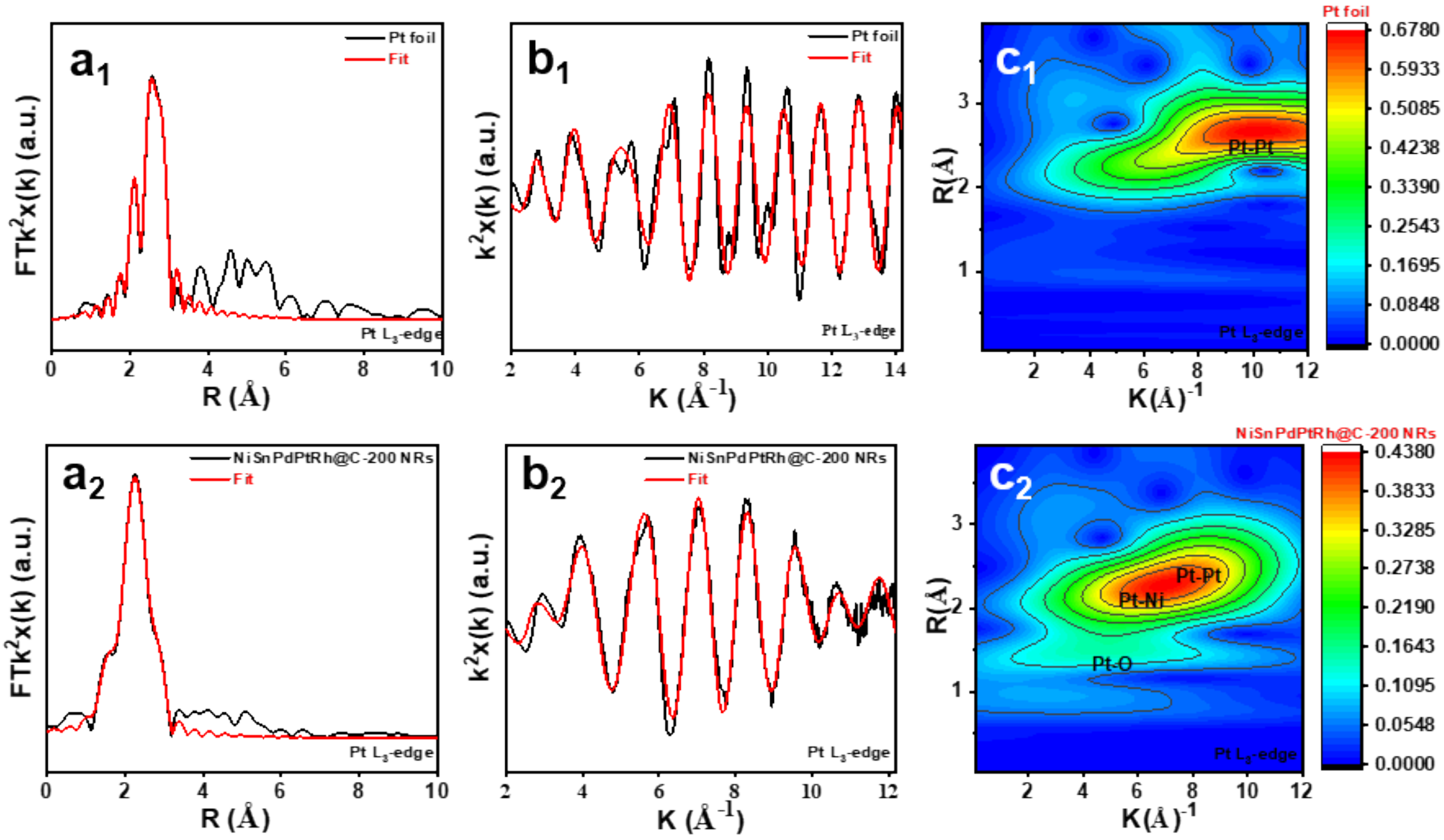

Fig. S23. Fourier-transformed EXAFS of Pt L_3-edge in R space (a_1 and a_2) and k space (b_1 and b_2), and the corresponding wavelet transform (WT) (c_1 and c_2) of Pt foil and NiSnPdPtRh@c-200 NRs.

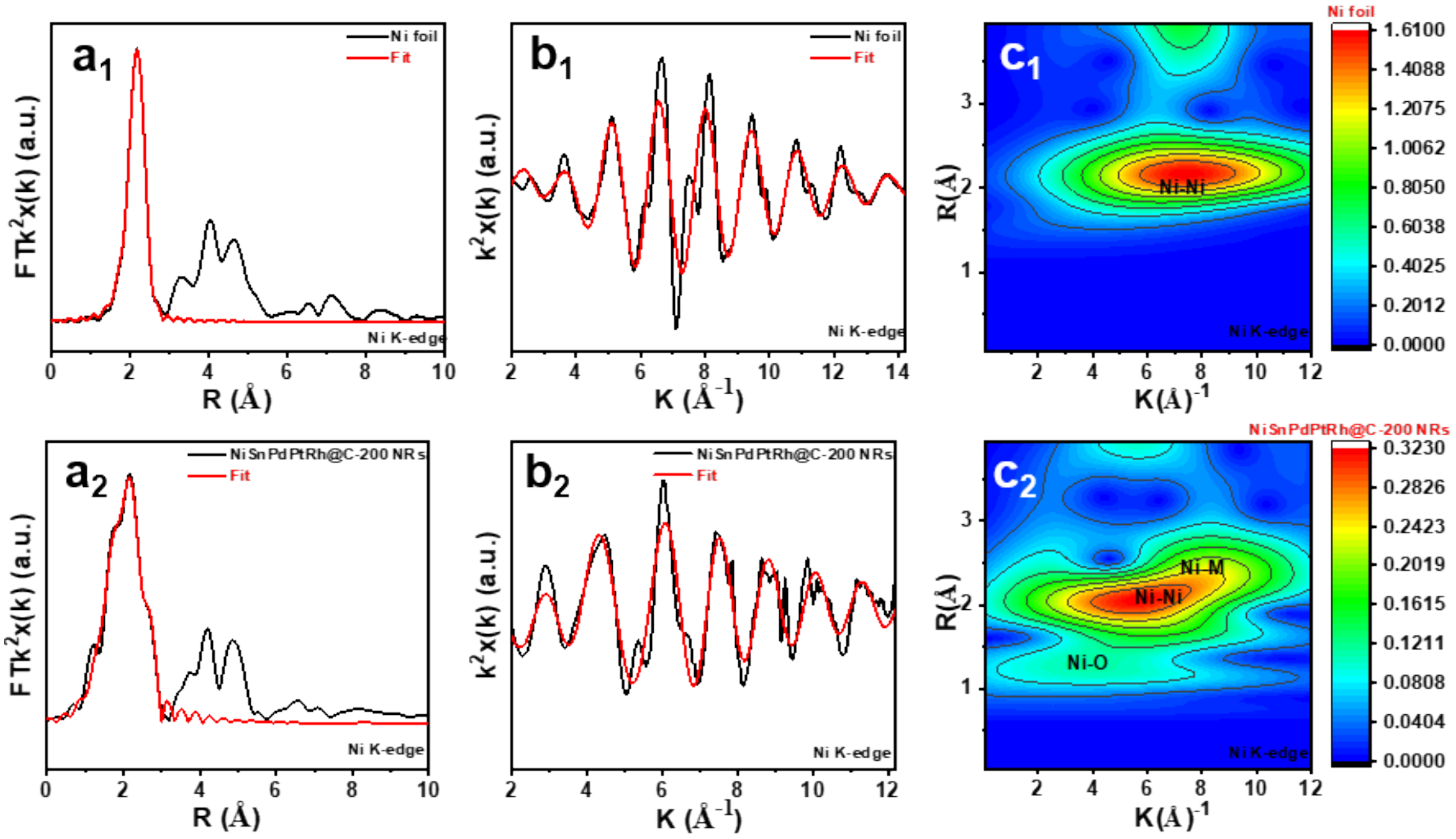

Fig. S24. Fourier-transformed EXAFS of Ni K-edge in R space (a_1 and a_2), k space (b_1 and b_2), and the corresponding wavelet transform (WT) (c_1 and c_2) of Ni foil and NiSnPdPtRh@c-200 NRs.

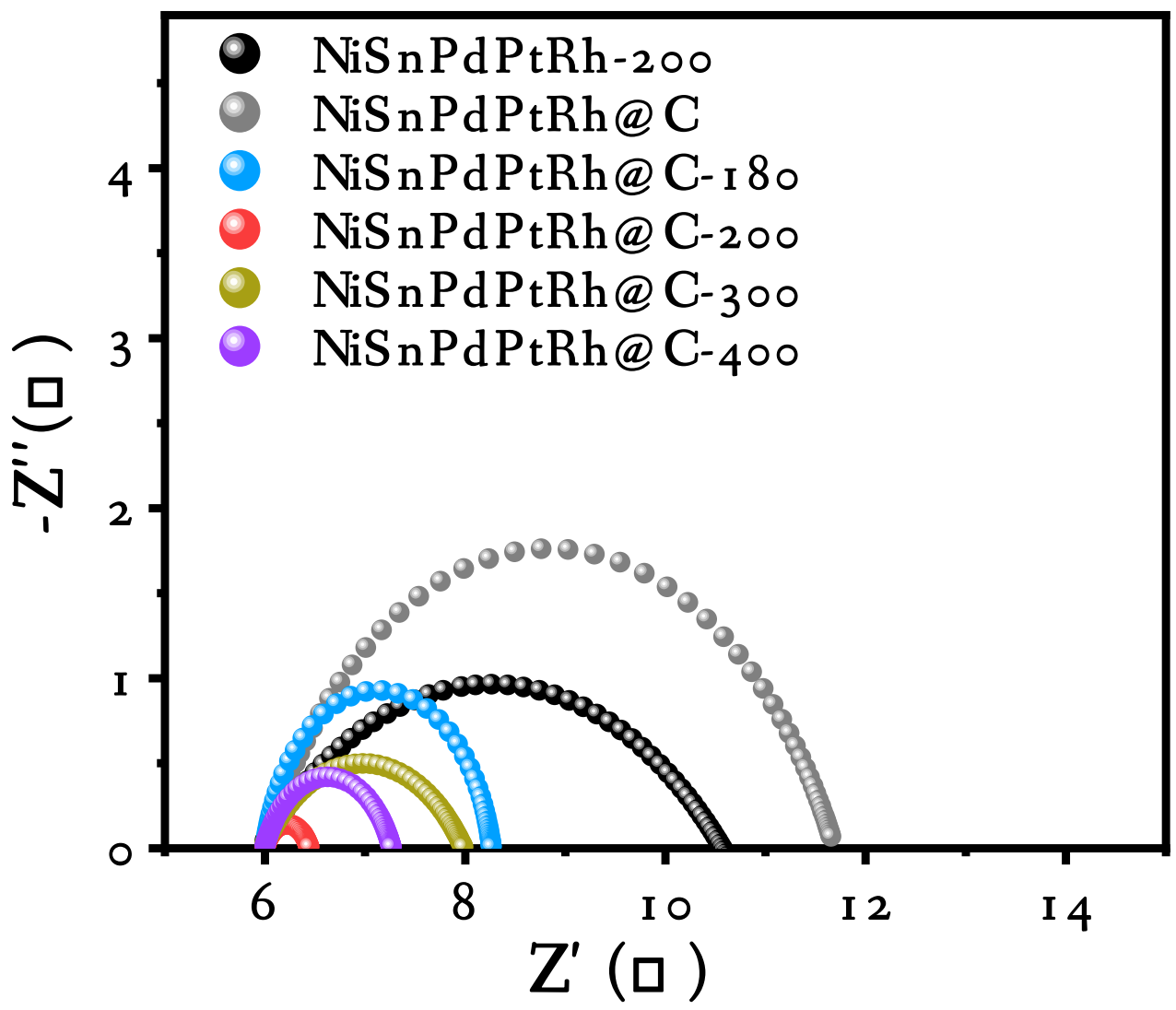

Fig. S25. EIS of NiSnPdPtRh NRs with different states for HER at 35 mV vs. RHE in 0.5M H_2SO_4 solution.

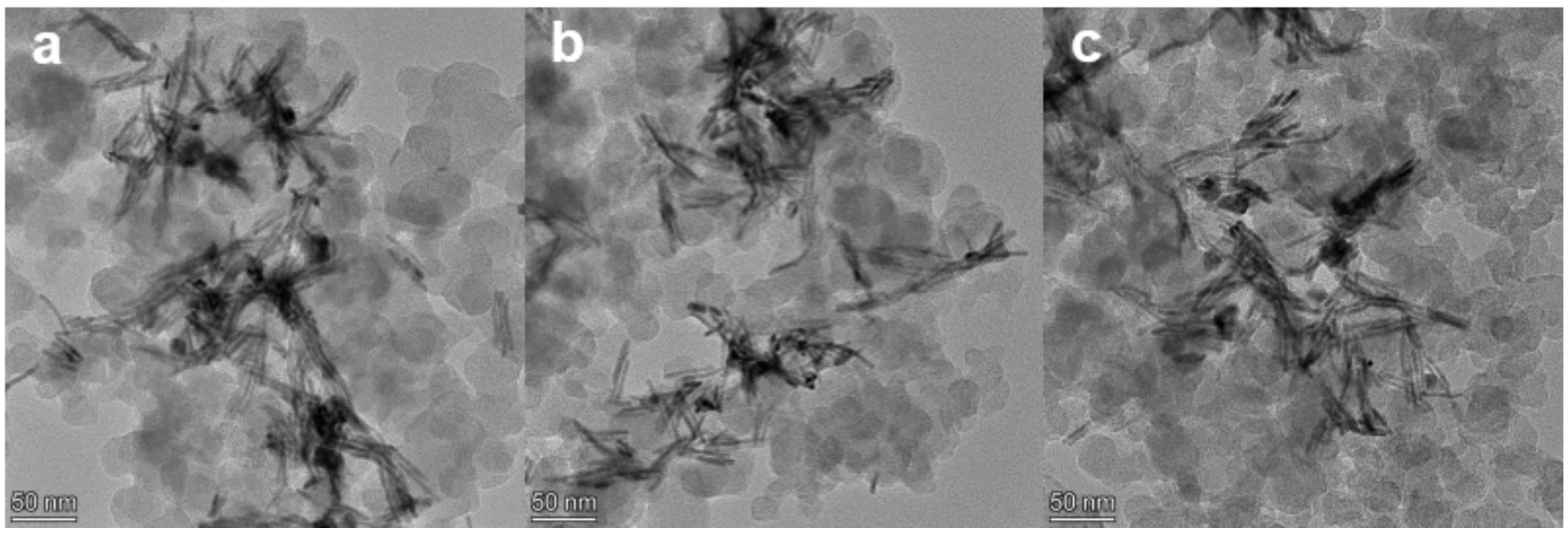

Fig. S26. TEM images of (a) SnPdRh@C-200 NRs, (b) NiSnPdRh@C-200 NRs and (c) NiSnPdPt@C-200 NRs prepared by using the same carbon-supported thermal treatment method.

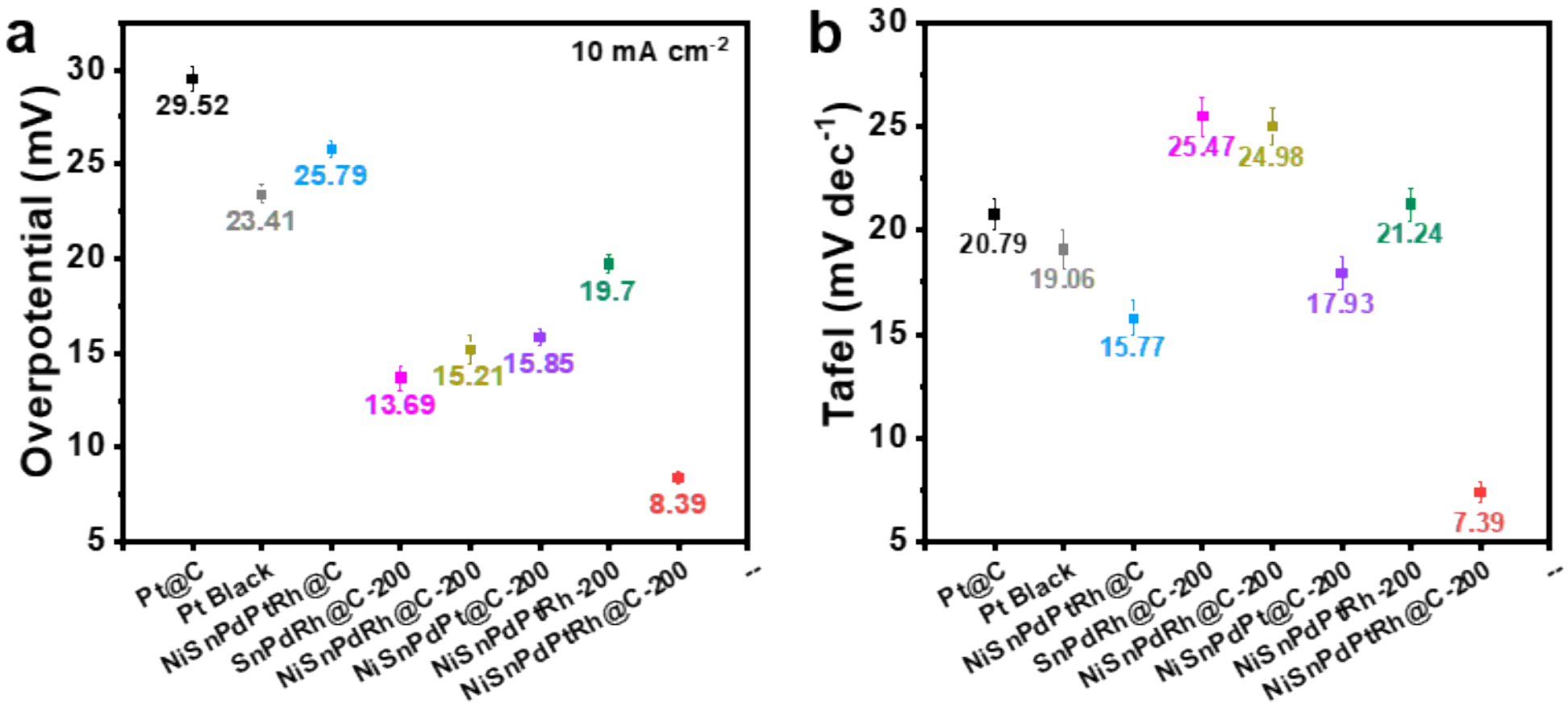

Fig. S27. Overpotential (ƞ₁₀) and Tafel slope of NiSnPdPtRh@C-200 NRs and the control samples.

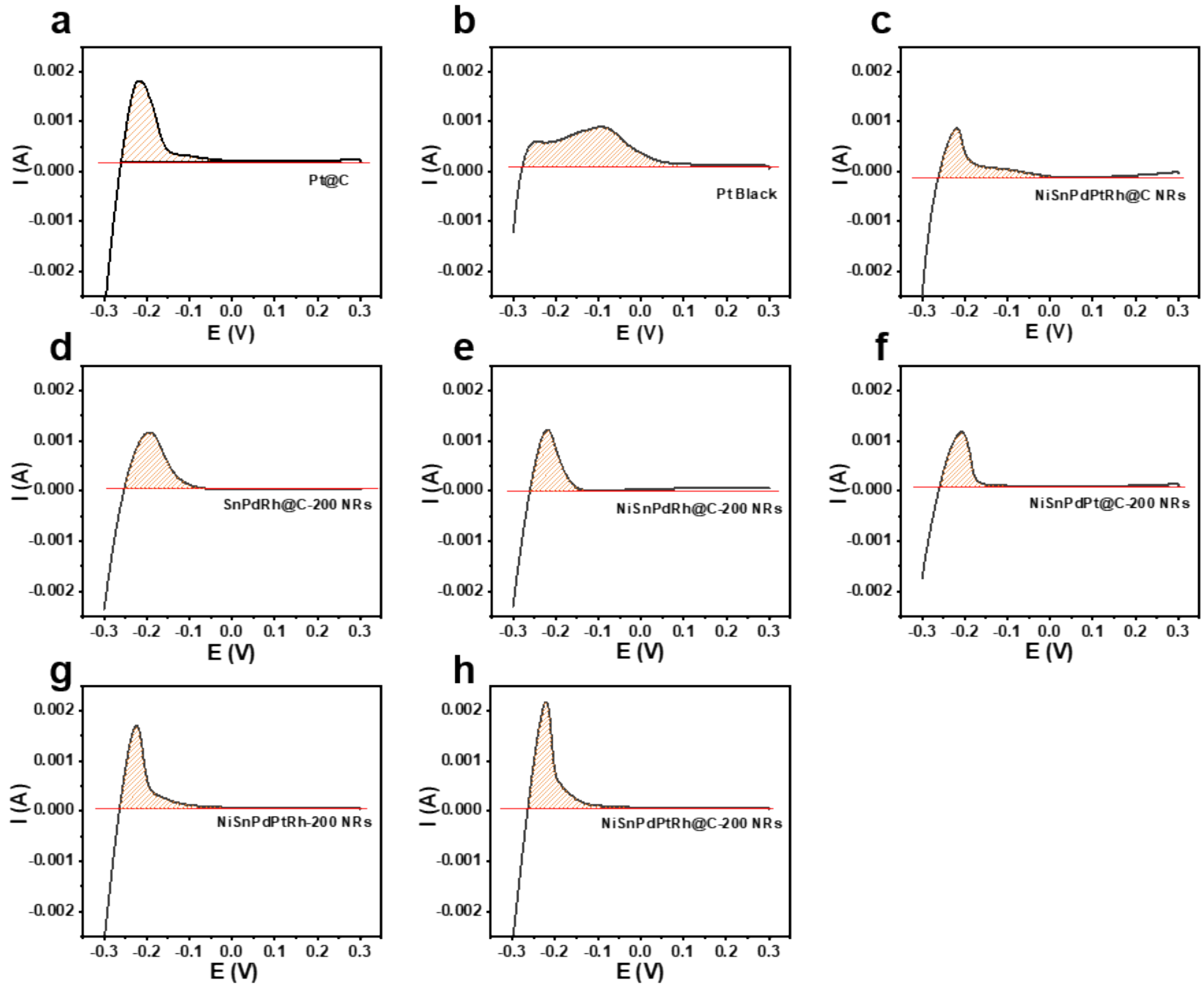

Fig. S28. CV curves at 0.1 V s^{-1} in 0.5 M H_2SO_4 solution. (a) Pt@C; (b) Pt Black; (c) NiSnPdPtRh-200 NRs; (d) SnPdRh@C-200 NRs; (e) NiSnPdRh@C-200 NRs; (f) NiSnPdPt@C-200 NRs; (g) NiSnPdPtRh@C NRs; (h) NiSnPdPtRh@C-200 NRs.

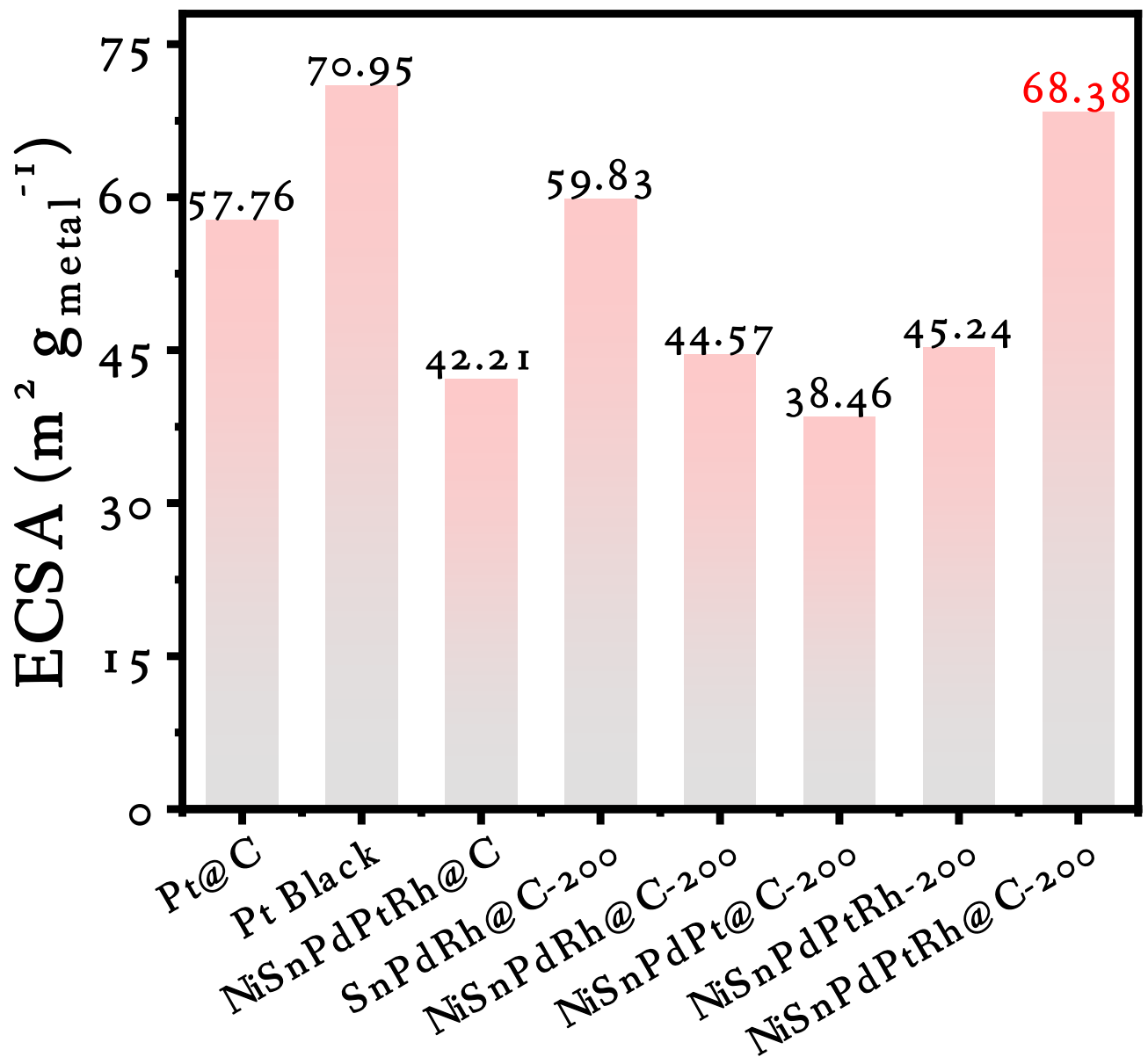

Fig. S29. ECSAs of NiSnPdPtRh@C-200 NRs and control samples.

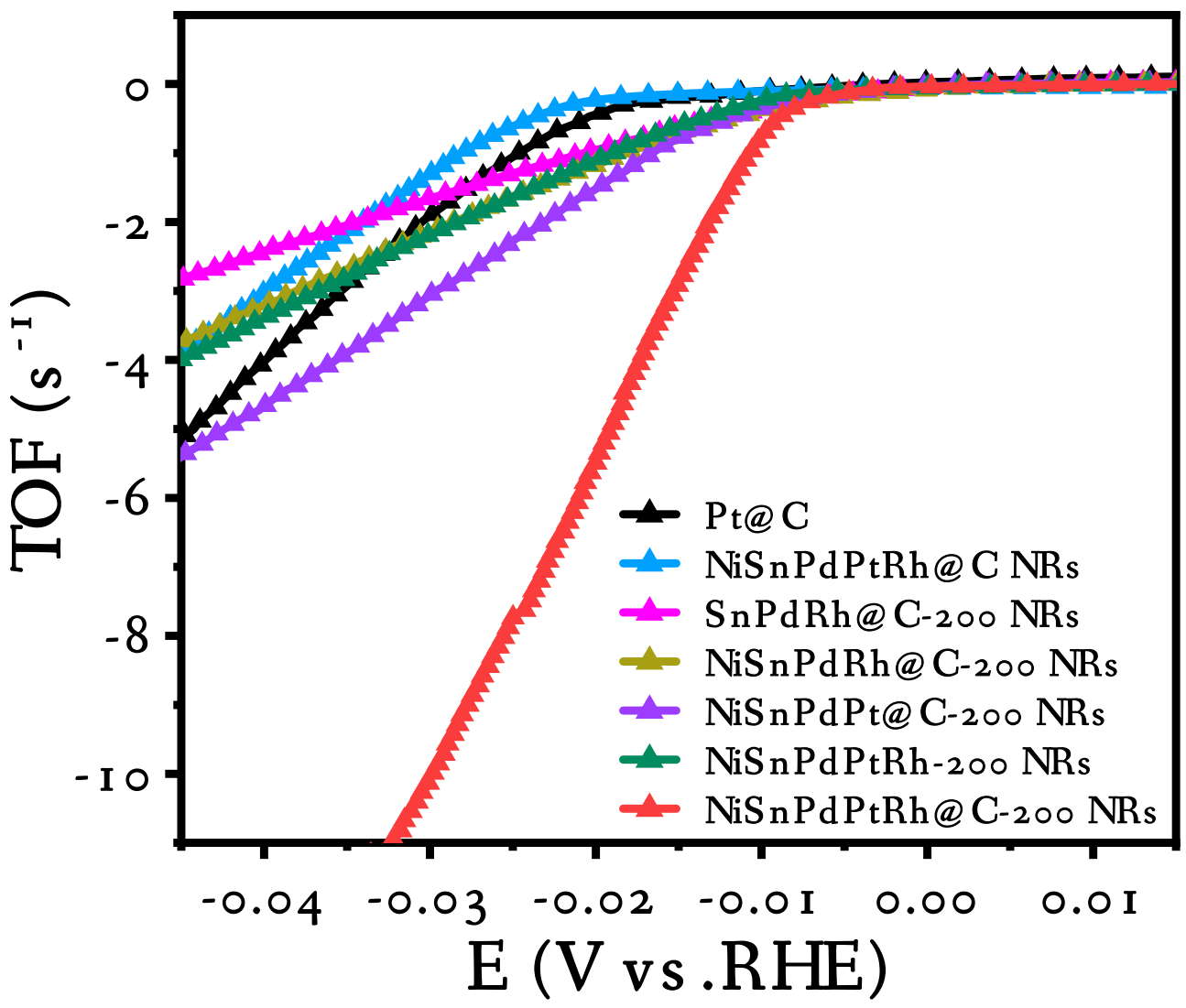

Fig. S30. The TOF curves of NiSnPdPtRh@C-200 NRs and the control catalysts.

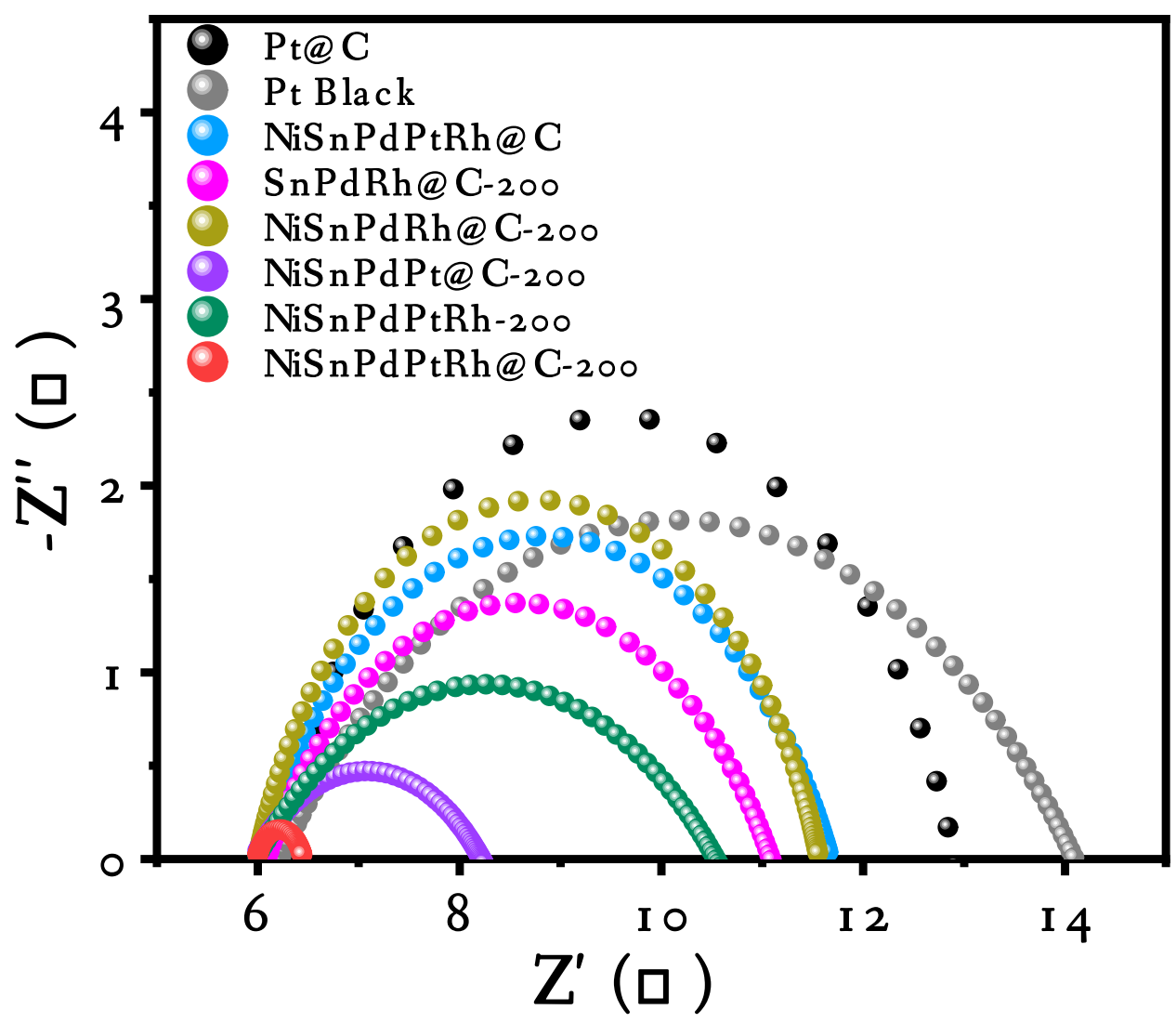

Fig. S31. EIS of NiSnPdPtRh@C-200 NRs and the control catalysts for HER at 35 mV vs. RHE in 0.5M H_2SO_4 solution.

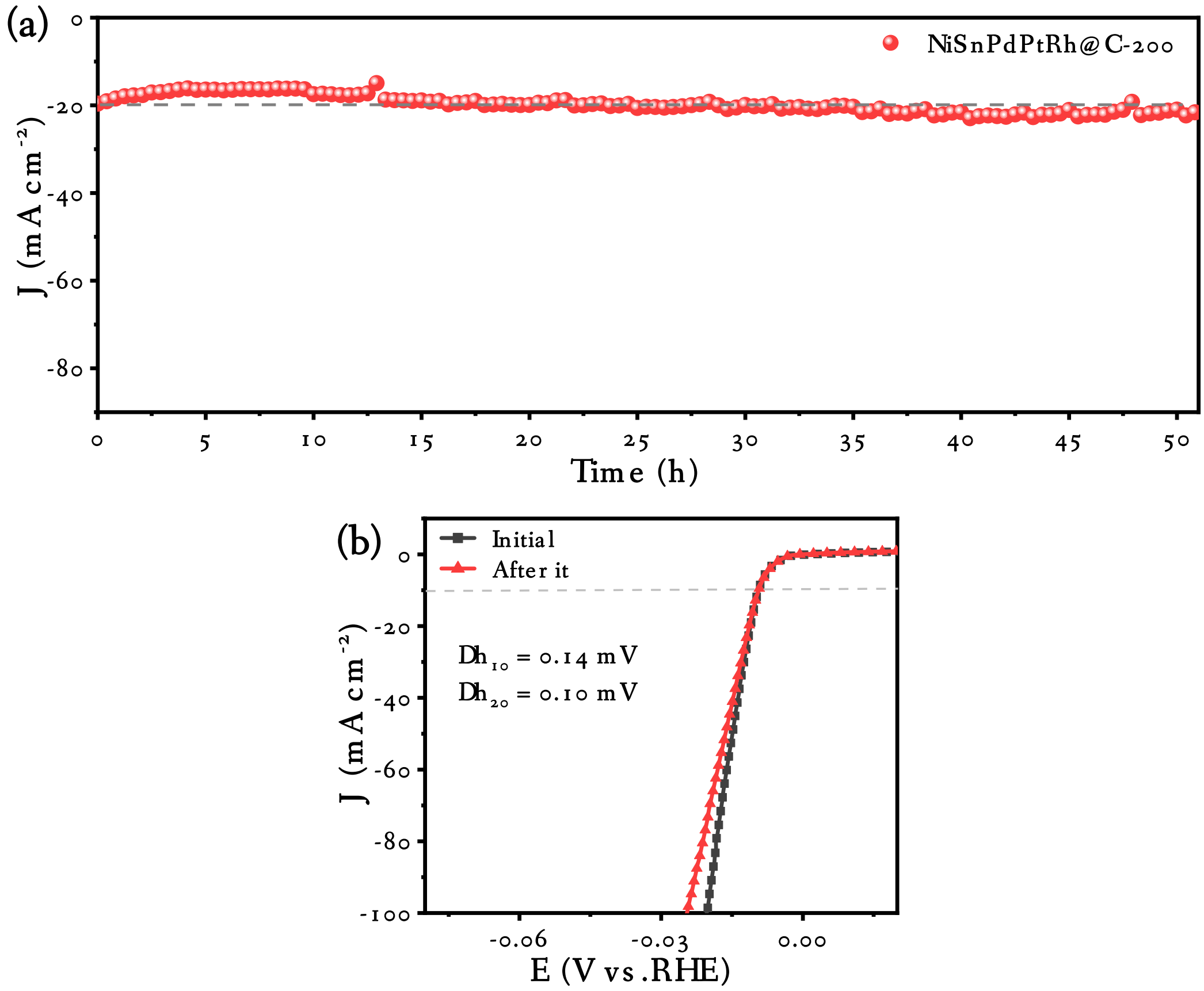

Fig. S32. Long-term stability and the accelerated durability test. (a) CV curve of NiSnPdPtRh@C-200 NRs at –0.012 V *vs.* RHE at 20 mA cm^{-2}. (b) The polarization curves for ADT before and after 50-hours CA test.

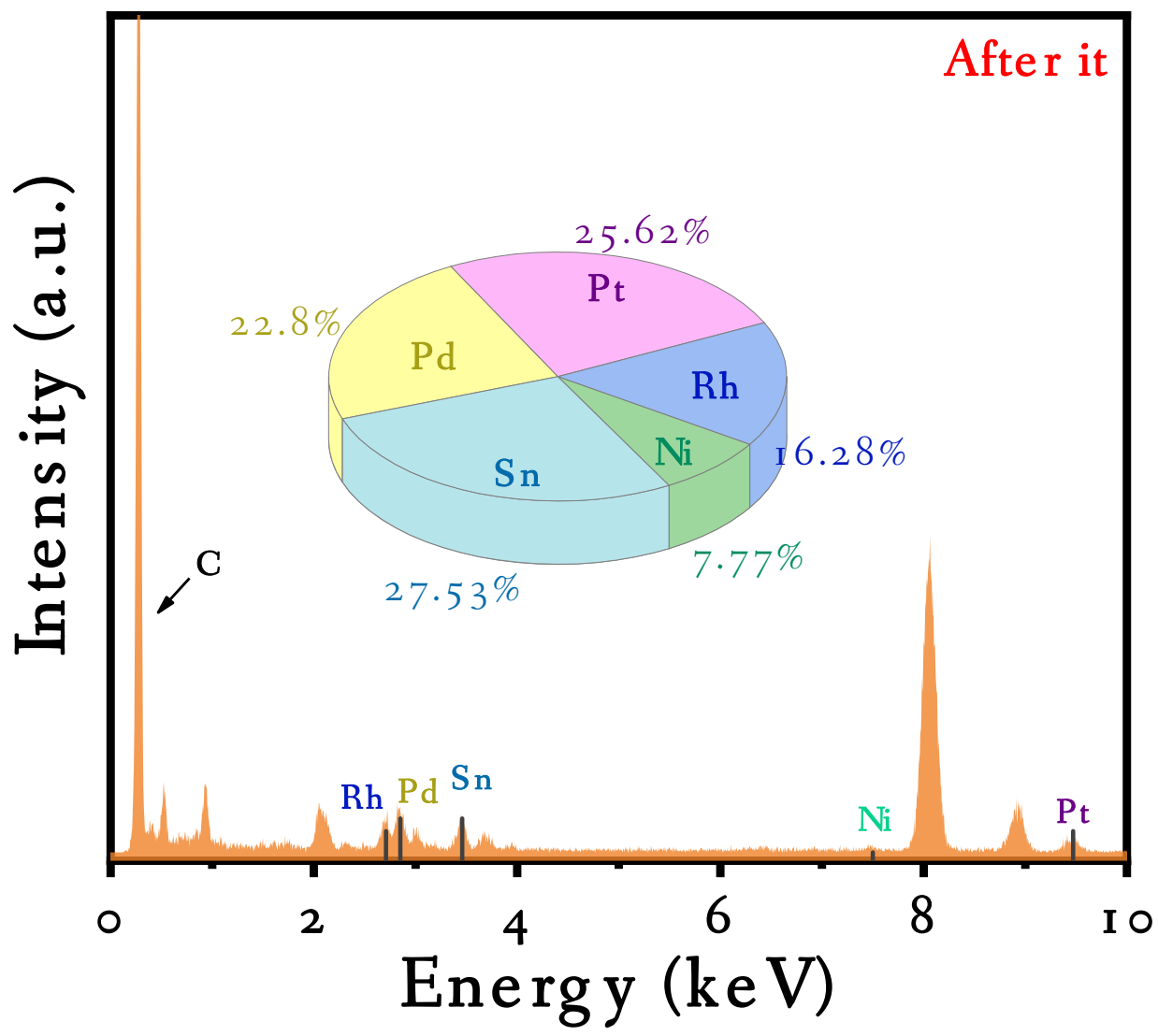

Fig. S33. The atomic percentage content of Ni, Sn, Pd, Pt, and Rh in the NiSnPdPtRh@C-200 nanorods, as quantified by EDS elemental mapping after 100 h CA test at –0.057 V vs. RHE.

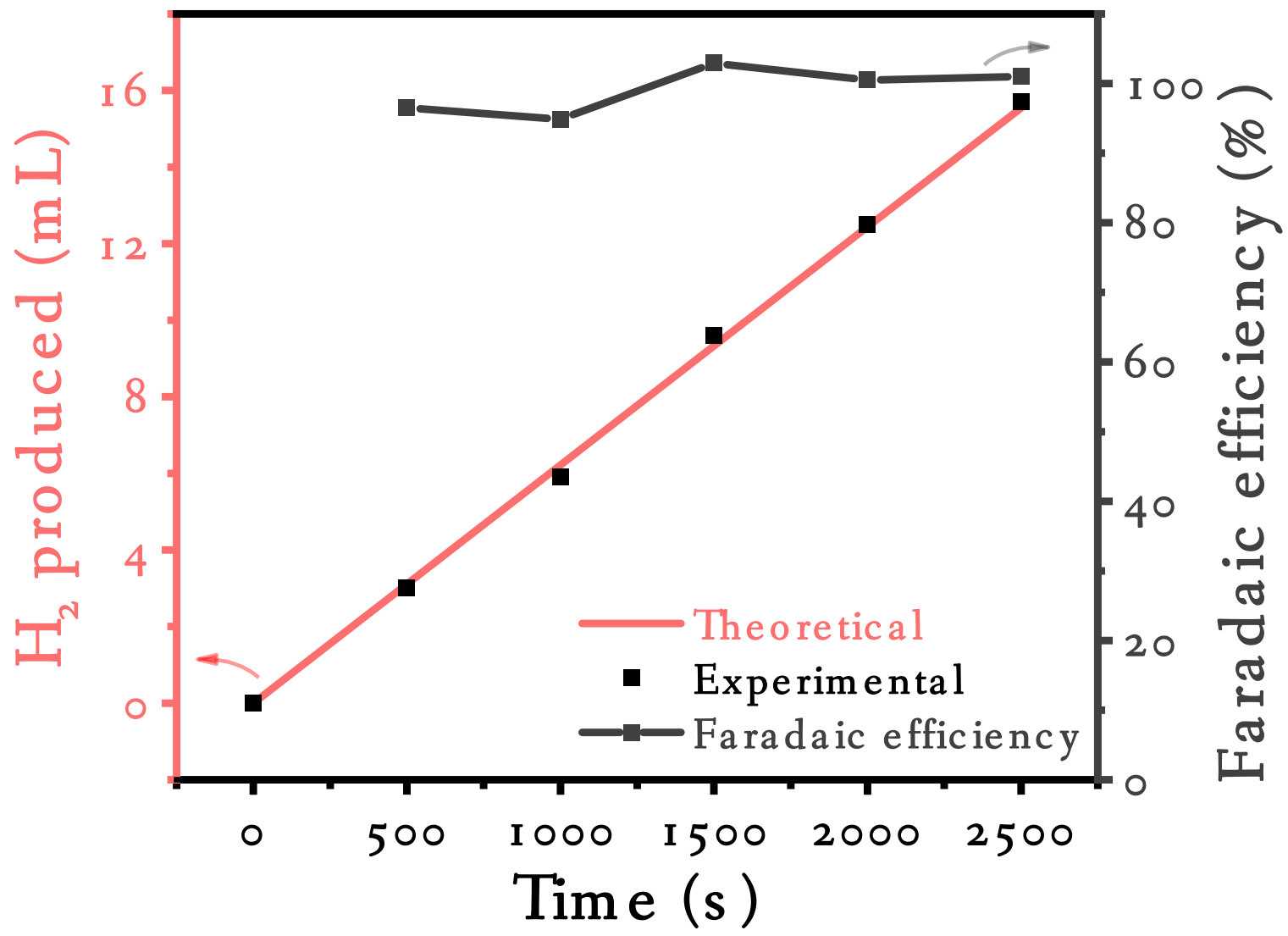

Fig. S34. The theoretically calculated and experimentally measured amount of H_2 versus time as well as its Faradaic efficiency for HER on the NiSnPdPtRh@C-200 NRs catalyst at a current density of 50 mA cm^{-2}.

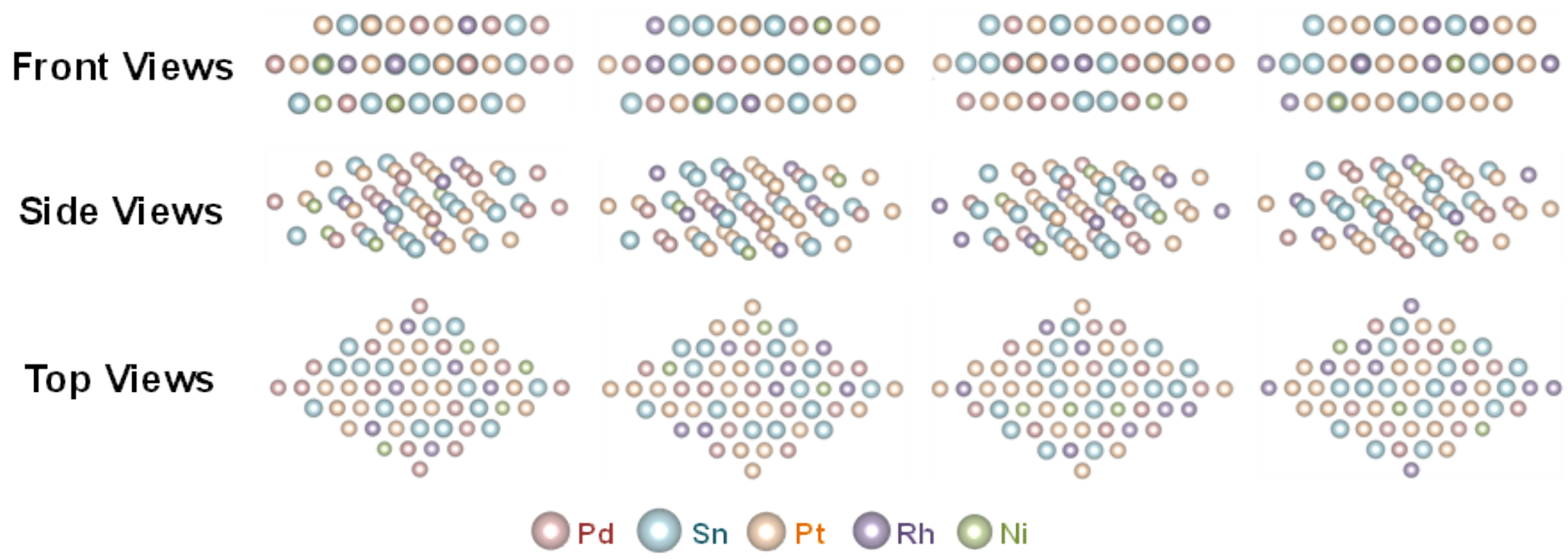

Fig. S35. The side, top, and front views of the structural model of NiSnPdPtRh HEA NRs with five different atomic distributions.

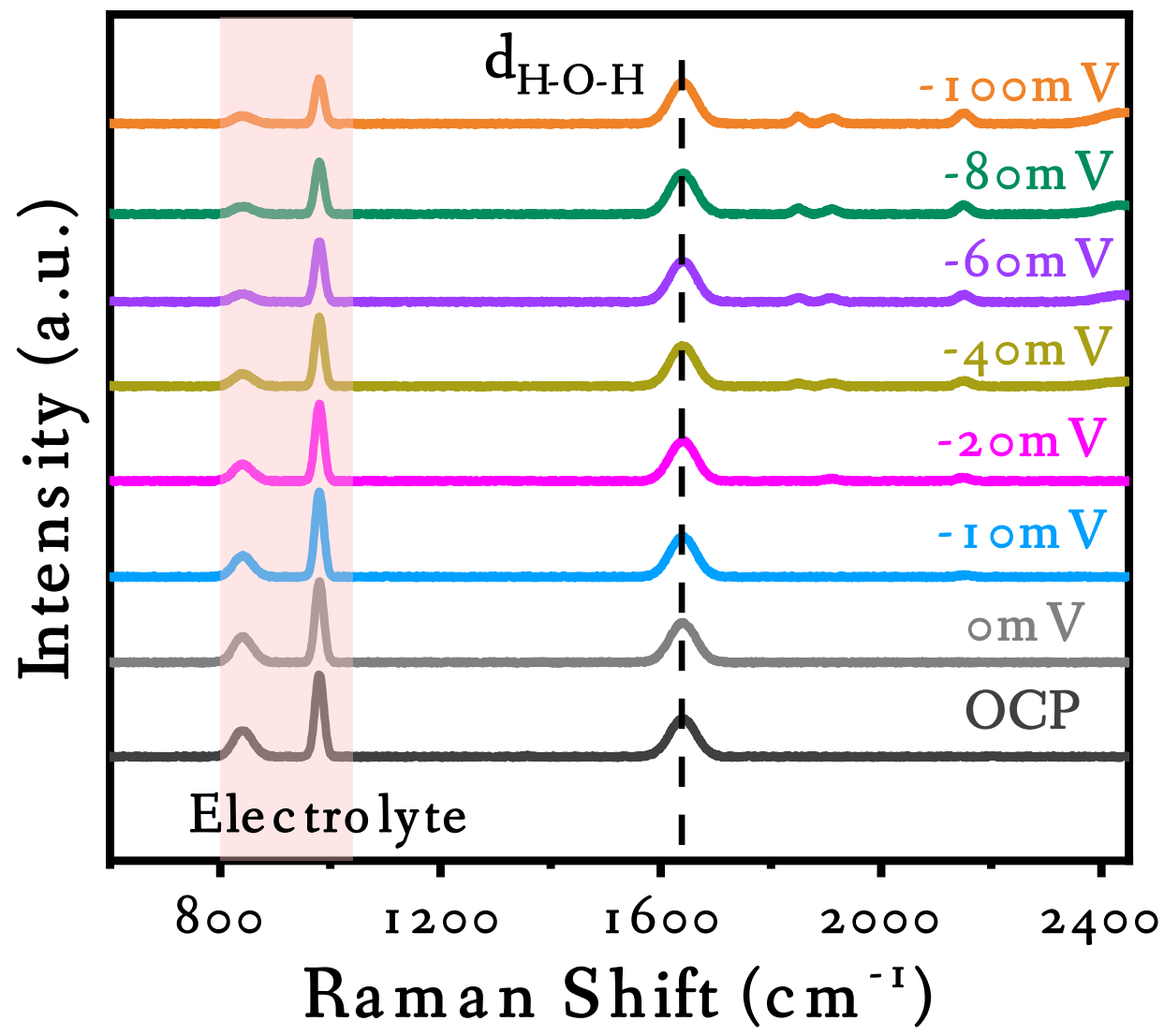

Fig. S36. In situ SERS with different applied potentials of NiSnPdPtRh@C-200 NRs.

Table S1. The atomic ratios of Ni, Sn, Pd, Pt, and Rh in NiSnPdPtRh HEA NRs measured by ICP-OES.

Elements	Ni	Sn	Pd	Pt	Rh
Atomic percentage (%)	6.8	31.34	24.34	23.6	13.92

Table S2. The reaction system temperatures under irradiation for 180 s with different microwave power and the yield of the as-prepared NiSnPdPtRh NRs.

Power	900 W	720 W	540 W
Temperature	∼262 ℃	∼214 ℃	∼202 ℃
Yield	80%	60%	37%

Table S3. The reaction system temperatures with different microwave irradiation time at 540 W and the yield of the as-prepared NiSnPdPtRh NRs.

Time	120 s	180 s	240 s	300 s	360 s
Temperature	∼182℃	∼202℃	∼226℃	∼242℃	∼262℃
Yield	13.5%	37.1%	54.3%	68.6%	77.1%

Table S4. The reaction system temperatures with different microwave irradiation time at 900 W and the yield of the as-prepared NiSnPdPtRh NRs.

Time	90 s	120 s	150 s	180 s	210 s
Temperature	∼192℃	∼204℃	∼224℃	∼252℃	∼262℃
Yield	23%	49%	67%	80%	83%

Table S5. The corresponding atomic percentage of the as-prepared NiSnPdPtRh NRs with different molar ratios of metal precursors.

Case	Precursor molar ratio	Atomic percentage (%) in products				
	Ni:Sn:Pd:Pt:Rh	6.80	31.34	24.34	23.60	13.92
Optimal case	1:2:1:1:1	9.54	26.61	23.17	23.65	17.03
Adjust Pt	1:2:1:0:1	9.62	25.11	39.88	0	25.40
	1:2:1:2:1	7.99	26.90	23.45	25.55	16.11
	1:2:1:3:1	7.56	25.13	15.59	39.87	11.85
Adjust Rh	1:2:1:1:0	10.47	30.65	32.17	26.71	0
	1:2:1:1:2	9.46	30.54	21.22	13.56	25.23
	1:2:1:1:3	5.79	26.78	21.98	14.41	31.05
Adjust Pd	1:2:0:1:1	17.71	26.01	0	30.46	25.82
	1:2:2:1:1	7.35	27.79	41.17	12.13	11.56
	1:2:3:1:1	4.74	22.94	62.99	3.80	5.52
Adjust Ni	0:2:1:1:1	0	28.08	32.63	20.40	18.89
	2:2:1:1:1	6.49	29.59	32.60	14.20	17.13
	3:2:1:1:1	5.83	28.12	14.09	38.42	13.54
Adjust Sn	1:0:1:1:1	4.41	0	41.40	27.99	26.21
	1:1:1:1:1	9.14	26.72	15.26	29.29	19.59
	1:3:1:1:1	9.86	29.04	9.53	31.41	20.16

Table S6. The mass contents and the corresponding atomic percentage of Ni, Sn, Pd, Pt, and Rh in NiSnPdPtRh NRs and NiSnPdPtRh@C-200 NRs measured by ICP-OES.

	NiSnPdPtRh NRs		NiSnPdPtRh@C-200 NRs	
	Mass loading (wt. %)	Atomic percentage (at. %)	Mass loading (wt. %)	Atomic percentage (at. %)
Ni	3.14	6.80	1.21	6.72
Sn	29.19	31.34	11.78	32.47
Pd	20.32	24.34	7.74	23.79
Pt	36.11	23.60	14.15	23.71
Rh	11.24	13.92	4.19	13.31
Total	100	100	39.07	100

Table S7. The binding energies of Ni^{0} $2p_{3/2}$, Pt^{0} $4f_{7/2}$, Rh^{0} $3d_{5/2}$, Pd^{0} $3d_{5/2}$ and Sn^{0} $3d_{5/2}$ of NiSnPdPtRh NRs and NiSnPdPtRh@C-200 NRs as well as the binding energy shifts of NiSnPdPtRh@C-200 NRs vs. NiSnPdPtRh NRs.

Element	Ni^{0} $2p_{3/2}$ (eV)	Pt^{0} $4f_{7/2}$ (eV)	Rh^{0} $3d_{5/2}$ (eV)	Pd^{0} $3d_{5/2}$ (eV)	Sn^{0} $3d_{5/2}$ (eV)
NiSnPdPtRh NRs	852.42	71.17	306.52	335.67	485.1
NiSnPdPtRh@C-200 NRs	853.03	71.67	307.26	336.17	485.75
ΔBE (Shift)	+0.61	+0.50	+0.74	+0.50	+0.65

Table S8. Structural parameters of coordination number (C.N.), bond distance (R), Debye-Waller factors (σ^2) and R factor from EXAFS fitting of Pt foil and NiSnPdPtRh@C-200 NRs. The data is k2-weighted and not phase-corrected, and the fitting range of NiSnPdPtRh@C-200 NRs is $3 < k < 11$ Å^{-1}, $1 < R < 3$ Å.

Sample	Bond type	C.N.	R (Å)	σ^2 (Å^2)	ΔE (eV)	R factor
Pt foil	Pt-Pt	12	2.76±0.01	0.0048	6.7±0.5	0.0029
NiSnPdPtRh@C-200	Pt-O	0.6±0.2	1.96±0.03	0.0011	5.5±1.8	0.0069
	Pt-Ni	3.1±0.5	2.60±0.01	0.0075		
	Pt-Pt	5.8±0.9	2.73±0.01	0.0062		

Table S9. Structural parameters of coordination number (C.N.), bond distance (R), Debye-Waller factors (σ^2) and R factor from EXAFS fitting of Ni foil and NiSnPdPtRh@C-200 NRs. The data is k2-weighted and not phase-corrected, and the fitting range of NiSnPdPtRh@C-200 NRs is $3 < k < 11.5$ Å^{-1}, $1 < R < 3$ Å.

Sample	Bond type	C.N.	R (Å)	σ^2 (Å^2)	ΔE (eV)	R factor
Ni foil	Ni-Ni	12	2.48±0.01	0.0060	6.5±0.4	0.0017
NiSnPdPtRh@C-200	Ni-O	1.8±0.6	2.05±0.03	0.0137	7.5±2.8	0.0125
	Ni-M(Pd/Rh)	4.3±0.8	2.59±0.03	0.0171		
	Ni-Pt	4.3±0.6	2.64±0.01	0.0081		

Table S10. Comparison of the overpotential at 10 mA cm^{-2} and Tafel slope of HEAs catalysts reported in the recent literatures for HER in 0.5 M H_2SO_4 solution.

Catalysts	ƞ$_{10}$ (mV vs.RHE)	Tafel slope (mV dec^{-1})	Ref.
**NiSnPdPtRh@C-200**	**8.39**	**7.92**	**This Work**
**Pt@C**	**29.94**	**28.13**	**This Work**
**Pt Blacck**	**23.75**	**26.29**	**This Work**
np-AlNiCoIrMo	18.5	33.2	[1]
PdPtCuNiP	62	73	[2]
PdMoGaInNi	13	179.8	[3]
$Pt_{34}Fe_5Ni_{20}Cu_{31}Mo_9Ru$	9	24	[4]
PtPdRhRuCu	13	87	[5]
IrPdRhMoW/C	15	35	[6]
PtFeCoNiCu-400	10.8	28.1	[7]
d-PtRhPdIrRu	25	22.6	[8]
Ru-PtFeNiCuW/CNTs	9	19.2	[9]
PdPtRuRhAu-3.14	70.07	30.3	[10]
$(FeCoNiCrZnCu)_3O_4$	133	116	[11]
$Fe_2Co_2Ni_2Ru_2Pt_{0.5}$	40.8	30.4	[12]
$(Fe_{41.08}Co_{25.31}Ni_{20.75}Nb_{12.86})_{97}Pt_3$	11	39.3	[13]
AuPtRuRhIr	45	52.4	[14]
NiCoFePtRh/C	27	30.1	[15]
$Pd_{0.2}Pt_{0.2}Ir_{0.2}Ru_{0.2}Rh_{0.2}$	42.2	36.7	[16]
AuPdFeNiCo	45	32	[17]
FeNiCuWRu	49	32.4	[18]
CoCrFeNiAl	73	39.7	[19]
CoPtPdAuCu	51	30	[20]
RhRuPtPdIr	58	42	[21]
CoNiFe(Cr/V)	45	35	[22]
$Al_{80}Ni_6Co_3Mn_3Y_5Au_3$	62	44	[23]
$Cr_{15}Fe_{20}Co_{35}Ni_{20}Mo_{10}$	107	41	[24]

Table S11. ΔG_{H^*} on the surface of NiSnPdPtRh@C-200 NRs.

Adsorption Site	ΔG_{H^*} (eV)	Adsorption Site	ΔG_{H^*} (eV)
Pt-Rh	−0.57	**Pt-Pt-Rh**	**0.01**
Pt-Rh	−0.56	Ni-Pd-Rh	−0.57
Pt-Pd	−0.31	Pt-Pd-Pd	−0.45
Pt	−0.28	Pt	−0.26
Pt	−0.21	Ni-Pd-Rh	−0.58
Pt-Pt-Pd	−0.41	Sn	0.78
Sn	0.58	**Ni-Pt-Pd**	**−0.03**
Sn	0.65	Ni-Pd	−0.26
Pt-Pd-Rh	−0.09	Pt-Pd-Pd	−0.33
Ni	0.17	Pt	−0.11
Pt-Pd-Rh	−0.14	Rh	−0.40
Pt	−0.46	Sn	0.72
Rh	−0.38	Pt-Pd	0.09
Sn	0.54		

References

[1] Jin, Z. , Lv, J. , Jia, H. , Liu, W. , Li, H. , Chen, Z. , Lin, X. , Xie, G. , Liu, X. , Sun, S. , and Qiu, H. Nanoporous Al-Ni-Co-Ir-Mo High-Entropy Alloy for Record-High Water Splitting Activity in Acidic Environments. *Small* **2019**, 15, 1904180.

[2] Jia, Z. , Nomoto, K. , Wang, Q. , Kong, C. , Sun, L. , Zhang, L. , Liang, S. , Lu, J. , and Kruzic, J. J. A Self-Supported High-Entropy Metallic Glass with a Nanosponge Architecture for Efficient Hydrogen Evolution under Alkaline and Acidic Conditions. *Advanced Functional Materials* **2021**, 31, 2101586.

[3] Fu, X. , Zhang, J. , Zhan, S. , Xia, F. , Wang, C. , Ma, D. , Yue, Q. , Wu, J. , and Kang, Y. High-Entropy Alloy Nanosheets for Fine-Tuning Hydrogen Evolution. *ACS Catalysis* **2022**, 12, 11955–11959.

[4] Chen, Z. , Wen, J. , Wang, C. , and Kang, X. Convex Cube-Shaped $Pt_{34}Fe_5Ni_{20}Cu_{31}Mo_9Ru$ High Entropy Alloy Catalysts toward High-Performance Multifunctional Electrocatalysis. *Small* **2022**, 18, 2204255.

[5] Kang, Y. , Cretu, O. , Kikkawa, J. , Kimoto, K. , Nara, H. , Nugraha, A. S. , Kawamoto, H. , Eguchi, M. , Liao, T. , Sun, Z. , Asahi, T. , and Yamauchi, Y. Mesoporous multimetallic nanospheres with exposed highly entropic alloy sites. *Nature Communications* **2023**, 14, 4182.

[6] Zhang, D. , Shi, Y. , Chen, X. , Lai, J. , Huang, B. , and Wang, L. High-entropy alloy metallene for highly efficient overall water splitting in acidic media. *Chinese Journal of Catalysis* **2023**, 45, 174–183.

[7] Chen, Z. W. , Li, J. , Ou, P. , Huang, J. E. , Wen, Z. , Chen, L. , Yao, X. , Cai, G. , Yang, C. C. , Singh, C. V. , and Jiang, Q. Unusual Sabatier principle on high entropy alloy catalysts for hydrogen evolution reactions. *Nature Communications* **2024**, 15, 359.

[8] Huang, X. , Wu, Z. , Zhang, B. , Yang, G. , Wang, H. , Wang, H. , Cao, Y. , Peng, F. , Li, S. , and Yu, H. Formation of Disordered High-Entropy-Alloy Nanoparticles for Highly Efficient Hydrogen Electrocatalysis. *Small* **2024**, 20, 2311631.

[9] Wang, C. , Zhang, J. , Miao, K. , Long, M. , Lai, S. , Zhao, S. , and Kang, X. Octahedral Nanocrystals of Ru-Doped PtFeNiCuW/CNTs High-Entropy Alloy: High Performance Toward pH-Universal Hydrogen Evolution Reaction. *Advanced Materials* **2024**, 36, 2400433.

[10] Wang, Q. , Qin, Y. , Xie, J. , Kong, Y. , Sun, Q. , Wei, Z. , and Zhao, S. Size-Controllable High-Entropy Alloys Toward Stable Hydrogen Production at Industrial-Scale Current Densities. *Advanced Materials* **2025**, 37, 2420173.

[11] Li, M. , Ye, X. , Guo, S. , Hou, M. , Yang, L. , Chen, K. , Gao, L. , Li, Y. , and Briois, P. High entropy design endows spinel compounds with excellent bifunctional catalysis for hydrogen production from water electrolysis. *International Journal of Hydrogen Energy* **2025**, 130, 644–653.

[12] Wei, Y. , Hu, Z. , Wang, K. , Wu, D. , Cheng, F. , Cheng, N. , Wu, C. , Ge, B. , and Song, L. Rapid synthesis of a highly dispersed FeCoNiRuPt high-entropy alloy bifunctional electrocatalyst and exploration of the catalytic mechanism. *Journal of Materials Chemistry A* **2025**, 13, 22483–22491.

[13] Li, Z. , Zhong, H. , Liu, X. , Chiang, F. , Li, R. , Chen, H. , Wang, X. , Wan, C. , Wu, Y. , Wang, H. , Jiang, S. , Zhang, X. , and Lu, Z. Dynamic Dissolution-Deposition Equilibrium Enables Unprecedented HER Stability in Acidic PEMWE. *Advanced Materials* **2025**, 37, e10703.

[14] Zhang, C. , Wang, Z. , Liu, C. , Bai, Y. , Liang, C. , Low, J. , and Xiong, Y. Rapid synthesis of subnanoscale high-entropy alloys with ultrahigh durability. *Nature Materials* **2026**, 25, 26–34.

[15] Feng, G. , Ning, F. , Song, J. , Shang, H. , Zhang, K. , Ding, Z. , Gao, P. , Chu, W. , and Xia, D. Sub-2 nm Ultrasmall High-Entropy Alloy Nanoparticles for Extremely Superior Electrocatalytic Hydrogen Evolution. *Journal of the American Chemical Society* **2021**, 143, 17117–17127.

[16] Lin, C. , He, Z. Y. , Lin, J. , Chang, C. , Hsiao, Y. , Lin, S. , Chen, Y. , Huang, Y. , Lee, S. , Pao, C. , Lin, K. , Cowan, A. J. , and Yang, T. Atomically Mixed High-Entropy-Alloy Nanoframes with 3D Subnanometer-Thick Electrocatalytic Surfaces. *Advanced Functional Materials* **2025**, 35, 2505927.

[17] Jeong, S. , Branco, A. J. , Bollen, S. W. , Sullivan, C. S. , and Ross, M. B. Universal pH electrocatalytic hydrogen evolution with Au-based high entropy alloys. *Nanoscale* **2024**, 16, 11530–11537.

[18] Lu, Z. , Sun, W. , Cai, P. , Fan, L. , Chen, K. , Gao, J. , Zhang, H. , Chen, J. , and Wen, Z. High-entropy alloy catalysts for advanced hydrogen-production zinc-based batteries. *Energy & Environmental Science* **2025**, 18, 2918–2930.

[19] Ma, P. , Zhao, M. , Zhang, L. , Wang, H. , Gu, J. , Sun, Y. , Ji, W. , and Fu, Z. Self- supported high-entropy alloy electrocatalyst for highly efficient H_2 evolution in acid condition. *Journal of Materiomics* **2020**, 6, 736–742.

[20] Chen, X. , Su, L. , Tan, Y. , Yuan, J. , Mou, Y. , Deng, W.-Q. , and Wu, H. Breaking the activity-durability trade-off in platinum electrocatalysts for hydrogen evolution through twin boundary engineering. *Journal of Colloid and Interface Science* **2025**, 691, 137385.

[21] Jing, L. , Zou, Y. , Goei, R. , Wang, L. , Ong, J. A. , Kurkin, A. , Li, Y. , Tan, K. W. , and Tok, A. I. Y. Conformal Noble Metal High-Entropy Alloy Nanofilms by Atomic Layer Deposition for an Enhanced Hydrogen Evolution Reaction. *Langmuir* **2023**, 39, 3142–3150.

[22] Nawaz, T. , Ahmad, S. , Abuzaid, W. , Mustafa, F. , Ahmad, W. , El-Khatib, S. , Kaddoura, M. J. , Alami, A. H. , Alawadhi, H. , and Egilmez, M. Enhanced electrocatalytic hydrogen evolution with CoNiFe(Cr/V) based high entropy alloy electrodes. *APL Materials* **2025**, 13, 051114.

[23] Ju, S. , Feng, J. , Zou, P. , Xu, W. , Wang, S. , Gao, W. , Qiu, H.-J. , Huo, J. , and Wang, J.-Q. A

robust self-stabilized electrode based on Al-based metallic glasses for a highly efficient hydrogen evolution reaction. *Journal of Materials Chemistry A* **2020**, 8, 3246–3251.

[24] Zhang, G. , Ming, K. , Kang, J. , Huang, Q. , Zhang, Z. , Zheng, X. , and Bi, X. High entropy alloy as a highly active and stable electrocatalyst for hydrogen evolution reaction. *Electrochimica Acta* **2018**, 279, 19–23.
